# Translation and psychometric properties of the Persian version of the Audit of Diabetes Dependent Quality of Life (IR-ADDQoL)

**DOI:** 10.1186/s12955-022-02071-0

**Published:** 2022-11-28

**Authors:** Zeinab Ghazanfari, Mohammad Mehdi Naghizadeh, Marzieh Hadavi, Fatemeh Naghizadeh Moghari, Ali Montazeri

**Affiliations:** 1 Public Health Department, School of Health, Ilam University of Medical Sciences, Ilam, Iran; 2 Health and Environmental Research Center, Ilam University of Medical Sciences, Ilam, Iran; 3 Non-Communicable Diseases Research Center, Fasa University of Medical Sciences, Fasa, Iran; 4 Internal Medicine Department, School of Medicine, Ilam University of Medical Sciences, Ilam, Iran; 5 Population Health Research Group, Health Metrics Research Center, Iranian Institute for Health Sciences Research, ACECR, Tehran, Iran; 6 Faculty of Humanity Sciences, University of Culture and Science, Tehran, Iran

**Keywords:** Quality of life, Psychometrics, Diabetes impact, ADDQoL, Iran

## Abstract

**Background:**

This study aimed to undertake linguistic validation and assess the psychometric properties of the Persian version of the Audit of Diabetes-Dependent Quality of Life (IR-ADDQoL) questionnaire in Iranian patients with type 1 and type 2 diabetes.

**Methods:**

The gold-standard linguistic-validation procedure required by the developer of the ADDQoL (see https://www.healthpsychologyresearch.com) including cross-cultural adaptation was followed. Validity and reliability of the Persian ADDQoL were then evaluated in a cross-sectional study of a sample of 153 patients with diabetes. Exploratory and confirmatory factor analyses were applied to assess structural validity. Internal consistency reliability was assessed.

**Results:**

Both forced one-factor and unforced four-factor solutions were extracted from the exploratory factor analysis that jointly accounted for 48% and 66.53% of the variance observed, respectively. Confirmatory factor analysis indicated an acceptable model fit for the Persian ADDQoL. Cronbach’s alpha showed excellent internal consistency for the questionnaire (alpha = 0.931 for the single scale).

**Conclusion:**

The Persian ADDQoL (IR-ADDQoL) showed adequate structural validity and excellent internal consistency. Therefore, it could be efficiently used to evaluate the impact of diabetes on quality of life in outcome studies and research settings in Iran.

**Supplementary Information:**

The online version contains supplementary material available at 10.1186/s12955-022-02071-0.

## Background

Diabetes mellitus (DM) is a major health problem on a global scale. At present the latest statistics provided by the International Diabetes Federation (IDF) indicate that 537 million adults aged between 20 and 79 are living with diabetes. The same source reported that currently there are 206 million diabetic patients in Western Pacific, 90 million in South East Asia, 73 million in Middle East and North Africa, 61 million in Europe, 51 million in North America and Caribbean, 32 million South and Central America, 24 million in Africa. It is predicted that the number will rise to 643 million by 2030 and 783 million by 2045 [1].

Iran, as the second-largest country in the Middle East (West Asia), is amongst the countries with the highest prevalence of DM in the region. In 2009 there were estimated to be 3.78 million cases of DM (2.74 million diagnosed and 1.04 million undiagnosed) in Iran and it is predicted that it will rise to 9.24 million cases (6.73 million diagnosed and 2.50 million undiagnosed) by 2030. The total expected annual cost of DM management was $3.64 (2009 US$) billion (including US$1.71 billion direct and US$1.93 billion indirect costs) in 2009 and is predicted to increase to $9.0 billion (including US$4.2 billion direct and US$4.8 billion indirect costs) by 2030 [2].

For the last twenty years, increasing attention has been devoted to the physical, psychological, and social aspects of life of people with diabetes. Thus, the assessment of the quality of life in this population has increased. It has been reported that diabetes has a negative influence on the physical, psychological condition of the patient, and his/her social functioning, and these, in turn, lead to a deterioration in the level of quality of life of patients with both type 1 and type 2 diabetes [3]. In addition to diabetes-related complications, other factors such as lifestyle change, physical well-being, quantity and quality of social relationships, intensive treatment regimens and episodes of hypoglycemia may lead to reduced quality of life. Although clinical treatments mostly focus on medical outcomes, quality of life is recognized as an essential patient-reported health outcome in people with diabetes and is an important part of a holistic approach to patient care [4]. As such studies on quality of life among patients with diabetes were carried out from different perspectives. For instance, a study from Spain indicated that quality of life in diabetic population was moderate and depended on several factors including age, gender, and poor glycaemic control [5]. A review of twenty studies on the effectiveness of psychological interventions on mental health and quality of life in people living with type 1 diabetes reported that psychological interventions promoted glucose control and significantly improved quality of life in people with type 1 diabetes [6]. A systematic review of longitudinal studies on changes in quality of life following hypoglycaemia in adults with type 2 diabetes stated that there was not enough evidence to suggest that hypoglycaemia influenced overall diabetes-specific quality of life [7]. In contrast a qualitative systematic review reporting on the impact of hypoglycaemia on the quality of life of family members of adults with type 1 or type 2 diabetes found that quality of life not only among people with diabetes but also among their family members could be affected. The study found that family members of diabetic patients experience the impact of hypoglycaemia as a major recurrent challenge in their lives [8].

There are a number of specific instruments to measure quality of life in people with diabetes. A recent review identified 17 specific measures and reported that the Appraisal of Diabetes Scale (ADS), Audit of Diabetes-Dependent QOL measure (ADDQOL), Diabetes Health Profile (DHP), and Problem Areas in Diabetes (PAID) were more proper questionnaires for assessing one or more aspects of diabetes-specific quality of life [4]. Furthermore, a review on suitability of patient-reported outcome measures used to assess the impact of hypoglycaemia on quality of life in people with diabetes reported that none of the hypoglycaemia-specific patient-reported outcome measures demonstrated satisfactory validity, reliability and responsiveness [9]. However, among such instruments, the ADDQoL is the only instrument that allows patients to indicate which aspects of life apply to them and how important they are to their quality of life [10].

The Audit of Diabetes Dependent Quality of Life (ADDQoL) is an individualized questionnaire that measures the impact of diabetes on quality of life. The design of the ADDQoL was influenced by the philosophy underlying the Schedule for the Evaluation of Individual Quality of Life (SEIQoL) interview method. The ADDQoL allows the respondent to indicate aspects of life which are not applicable to them, rate the amount of impact of diabetes, maximum negative to positive, on the applicable aspects of life, and rate the perceived importance of each applicable aspect of life for their quality of life [11]. The ADDQoL was developed in the United Kingdom and has been linguistically and psychometrically validated in many countries including China [12], Japan [13], Italy [14], Portugal [15], Poland [16], Taiwan [17], Singapore [18], Slovenia [19], Lithuania [20], Australia [21], and Norway [22] just to name a few. Since the questionnaire was not validated in Iran, this study aimed to validate a Persian version of ADDQoL (IR-ADDQoL) linguistically and psychometrically.

## Methods

### Instrument

The Audit of Diabetes Dependent quality of life (ADDQoL) assesses diabetes impact on 19 life domains life such as working life, family life, freedom to eat as I wish, and self-confidence. There are two overview items to measure generic and diabetes-specific quality of life. Generic Quality of Life (GQoL) indicates how respondents feel about their present quality of life (score ranging from 3: excellent to -3: extremely bad), and the Diabetes-Dependent Quality of Life (DDQoL) asks patients to evaluate what their quality of life would be if they did not have diabetes (score ranging from -3: very much better to 1: worse). The 19 domain-specific items of the ADDQoL, consist of 2 parts. In part ‘a’, the individual rates the impact of diabetes on specific domains (score ranging from -3: e.g. very much greater to + 1: e.g. less). In part ‘b’ the individuals rate the importance of each specific domain (ranging from + 3: very important to 0: not at all important) [12, 13, 17].

### Scoring

Scoring for the ADDQoL is derived from two parameters: impact score and importance. The impact score is multiplied by the corresponding importance score to provide a weighted impact score for each domain (scores ranging from − 9: maximum negative impact to + 3: maximum positive impact). The lower the value of the weighted impact score, the greater the negative impact of diabetes on that aspect of life. Then, weighted impact scores for each individual are summed and divided by the number of applicable domains, to give an overall Average Weighted Impact score [AWI = ∑ (impact × importance scores) divided by number of applicable items]. Some domains (working life, holiday, family life, close personal relationship, and sex life) have a ‘not applicable’ (N/A) option. N/A responses are excluded from the individual’s AWI score [12, 13, 17].

### Translation and linguistic validation

The ADDQoL was linguistically validated into Person and use in the study under license from Professor Clare Bradley via her company Health Psychology Research (HPR) Ltd, High St Egham. www.healthpsychologyresearch.com (License for the IR-ADDQoL version: CB35). Once licensed, the ADDQoL was linguistically validated from the source English (UK) into Persian using a standardized methodology of forward and backward translation based on the guidelines of the MAPI Research Institute [23]. The forward translation (FT) was conducted independently by two Iranians, both fluent in English. The two forward translations were compared and reconciled into a single version by a third Persian speaker (FT-rec). Then, two other bilingual translators were recruited and, independently, back-translated (BT) the ADDQoL from Persian into English. The BTs were compared with the original English ADDQoL and a BT report was compiled and sent to the developer’s linguistic validation team at HPR. A process of discussion and revision between the project manager (AM) and the HPR’s linguistic validation team was repeated with further BTs as needed until a linguistically comparable and satisfactory version was achieved. The latest version was reviewed and evaluated by an endocrinologist to advise whether the wording of the Persian version might be improved to be more appropriate and/or understandable by patients. Finally, five volunteers with diabetes (of varying ages, gender, education and type of diabetes) were interviewed with a view to assessing and, where needed, improving the comprehensibility of the questionnaire for patients (cognitive debriefing step—CD). A CD report was completed and sent to HPR’s linguistic validation team for further review and discussion before the IR-ADDQoL was finalized. In doing so we asked participants to describe every single item in their own words to ensure that they have understood and comprehend the items correctly. The same procedure was applied for response categories, too.

### Psychometric evaluation

A cross-sectional study was conducted in a teaching hospital affiliated to of Ilam University of Medical Sciences, Illam, Iran. All patients attending internal and endocrinology outpatient clinics for routine care and patients attending the hospital for special medical care were approached by a research fellow and were asked to fill in the IR-ADDQoL questionnaire if they met inclusion criteria: age ≥ 15, ability to comprehend and speak Persian language, physician diagnosed type 1 or type 2. Patients with secondary diabetes, gestational diabetes, and patients who were unaware of their medical information were excluded from the study. A total number of 170 patients were recruited from February 2019 to June 2019 of whom 17 patients were excluded. Thus data from the remaining 153 patients were used for psychometric evaluation.

### Data analysis

The data were analyzed using SPSS for windows, version 26.0 and LISREL version 8.80. Descriptive statistics was computed to summarize the socio-demographic and clinical characteristics. The evaluation of scale structure was undertaken using one-factor forced and unforced exploratory factor analyses with varimax rotation. Kaiser–Meyer–Olkin measure of sampling adequacy and Bartlett’s test of sphericity also were examined. Factor loading equal or above 0.4 was considered acceptable. Confirmatory factor analyses were applied to examine one- and four-factor models using the likelihood estimation approach using the following fit indices: the root mean score error of approximation (RMSEA), the standardized root mean square residual (SRMR), the comparative fit index (CFI), and the non-normed fit index (the Tucker–Lewis index-NNFI). The acceptable cut-off values for RMSEA, SRMR, CFI and NFI were considered as follows: 0.06 or less, 0.08 or less, 0.95, and 0.95 respectively [24, 25]. Finally, internal consistency reliability was assessed by calculating Cronbach’s alpha coefficient for the scale formed by the domain-specific items.

## Results

### Linguistic validation

As indicated in the methods during the forward (FT) and backward translation (BT) process discussion and revision between the project manager (AM) and the HPR’s linguistic validation team was repeated until a linguistically comparable and satisfactory version was achieved. As example two detailed reports are supplemented (Additional file 1 and 2).

### Psychometric evaluation

A total of 153 patients with diabetes responded to the questionnaire (109 females, and 44 males). The mean age of participants was 47.40 ± 11.49 years and duration of diabetes since diagnosis was 8.79 ± 7.05 years. The majority of the respondents were married (83.7%) and less educated (78.4%). Of the study participants, 85.5% had type 2 diabetes, 51% were on oral ant-diabetic treatment while 30.7% were on insulin therapy. Participants who were less educated experienced a significantly greater negative impact of diabetes compared to well-educated patients (P < 0.021). No other significant differences were found in average weighted impact score by demographic and clinical data. The detailed socio-demographic and clinical characteristics of patients and their average weighted impact scores are presented in Table 1. Structural validity was examined by both exploratory and confirmatory factor analyses. These are described as follows:

**Table 1:** The characteristics of study sample and average weighted impact (AWI) scores

	No. (%)	Mean AWI (SD)	P − value
*Gender*			0.100
Male	44 (28.8)	− 3.85 (2.48)	
Female	109 (71.2)	− 3.18 (2.19)	
*Marriage*			0.934
Single	14 (9.2)	− 3.16 (2.44)	
Married	128 (83.7)	− 3.39 (2.34)	
Widowed/Divorced	11 (7.2)	− 3.35 (1.40)	
*Education*			0.021
Not or Less educated	120 (78.4)	− 3.59 (2.285)	
Well educated	33 (21.6)	− 2.56 (2.13)	
*Economic status*			0.208
Poor	21 (13.8)	− 3.69 (2.24)	
Intermediate	91 (59.9)	− 3.55 (2.30)	
Good	40 (26.3)	− 2.83 (2.28)	
*Diabetes type*			0.898
Type 1	21 (13.8)	− 3.43 (2.15)	
Type 2	130 (85.5)	− 3.36 (2.33)	
*Diabetes management method*			0.288
Tablet	78 (51)	− 3.13 (2.21)	
Insulin	47 (30.7)	− 3.34 (2.18)	
Both	18 (11.8)	− 3.97 (2.89)	
Diet only	10 (6.5)	− 4.31 (2.05)	
*Diabetes duration (year)*			0.549
< 10	90 (58.8)	− 3.28 (2.28)	
≥ 10	63 (41.2)	− 3.50 (2.31)	
*Complications*			0.937
No	50 (32.7)	− 3.35 (2.24)	
Yes	103 (67.3)	− 3.38 (2.32)	
*Having physical activity*			0.248
No	75 (49)	− 3.59 (1.99)	
Yes	78 (51)	− 3.16 (2.54)	

a. Exploratory factor analysis (EFA): This was performed by the principal axis factoring. Kaiser–Meyer–Olkin measure of sampling adequacy for ADDQoL was 0.914 and Bartlett’s test of sphericity was significant (X^2^ = 1742, df = 171, P < 0.001) that indicated the assumption was met for the factor analysis. When a one-factor solution was forced, item loadings ranged from 0.233 to 0.820, accounting for 48% of the variance observed. When an alternative unforced model was tested, a four-factor solution emerged. The four-factor solution accounted for 66.53% of the variance. The four factors could be described as social interactions, physical appearance and living conditions (8 items), recreation (4 items), eating (2 items) and independence and security (5 items). The results are presented in Table 2.

**Table 2:** The results obtained from exploratory factor analysis (unforced factor analysis)*

	Factor 1	Factor 2	Factor 3	Factor 4
Leisure activities	0.298	**0.736**	0.054	− 0.028
Working life	0.173	0.420	0.006	**0.656**
Local or long-distance journeys	0.192	**0.701**	0.174	0.388
Holidays	0.256	**0.583**	0.297	0.372
Physical health	0.316	**0.678**	0.310	0.044
Family life	**0.645**	0.341	0.253	0.199
Friendship and social life	**0.620**	0.453	0.247	0.246
Closest personal relationship	0.417	− 0.253	− 0.145	**0.611**
Sex life	0.275	0.439	0.325	**0.447**
Physical appearance	**0.542**	0.332	0.467	0.280
Self-confidence	**0.767**	0.276	0.098	0.227
Motivation	**0.625**	0.448	0.248	0.159
People’s reaction	**0.565**	− 0.051	0.497	0.107
Feelings about the future	**0.664**	0.269	0.336	0.086
Financial situation	0.433	0.217	0.182	**0.572**
Living conditions	**0.673**	0.345	0.166	0.281
Dependence on others	0.025	0.102	0.293	**0.665**
Freedom to eat	0.260	0.253	**0.854**	0.101
Freedom to drink	0.241	0.221	**0.828**	0.157
*Eigenvalue*	9.095	1.373	1.170	1.002
*Variance explained*	48.87	7.23	6.16	5.27

**Fig. 1:**
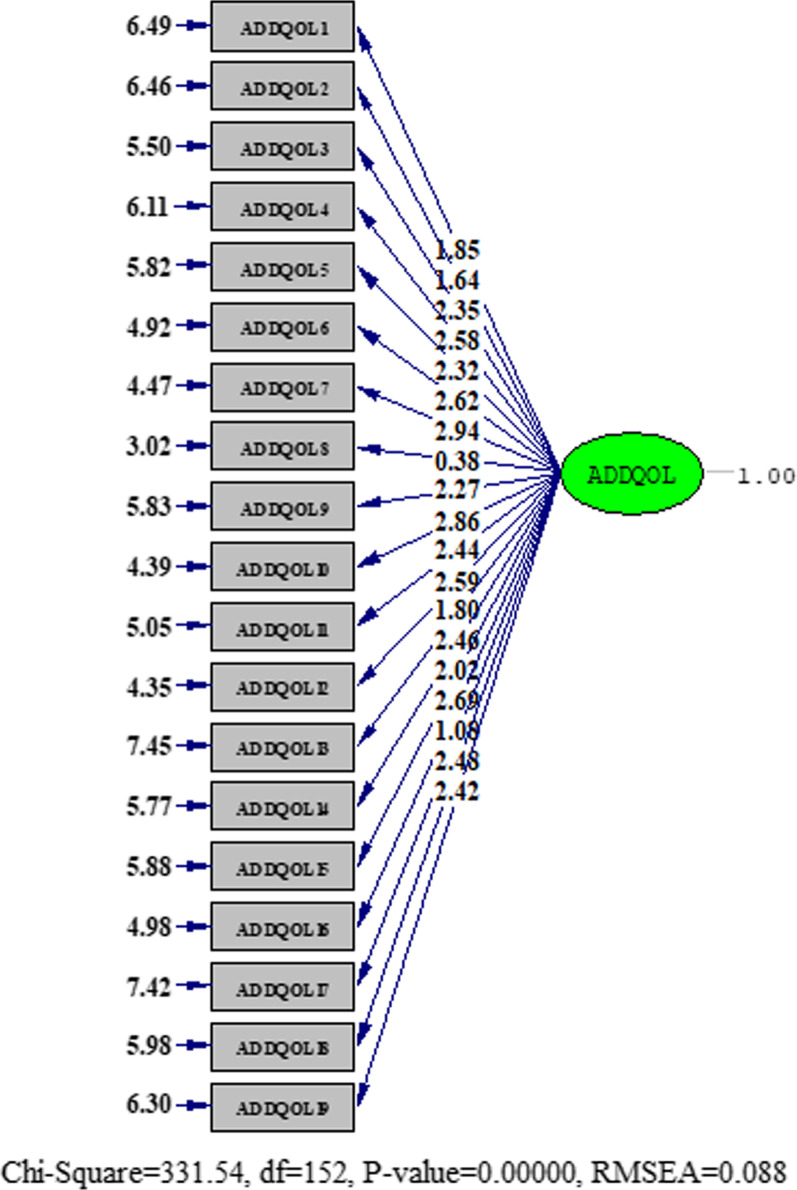
The results for one-factor model obtained from confirmatory factor analysis

**Fig. 2:**
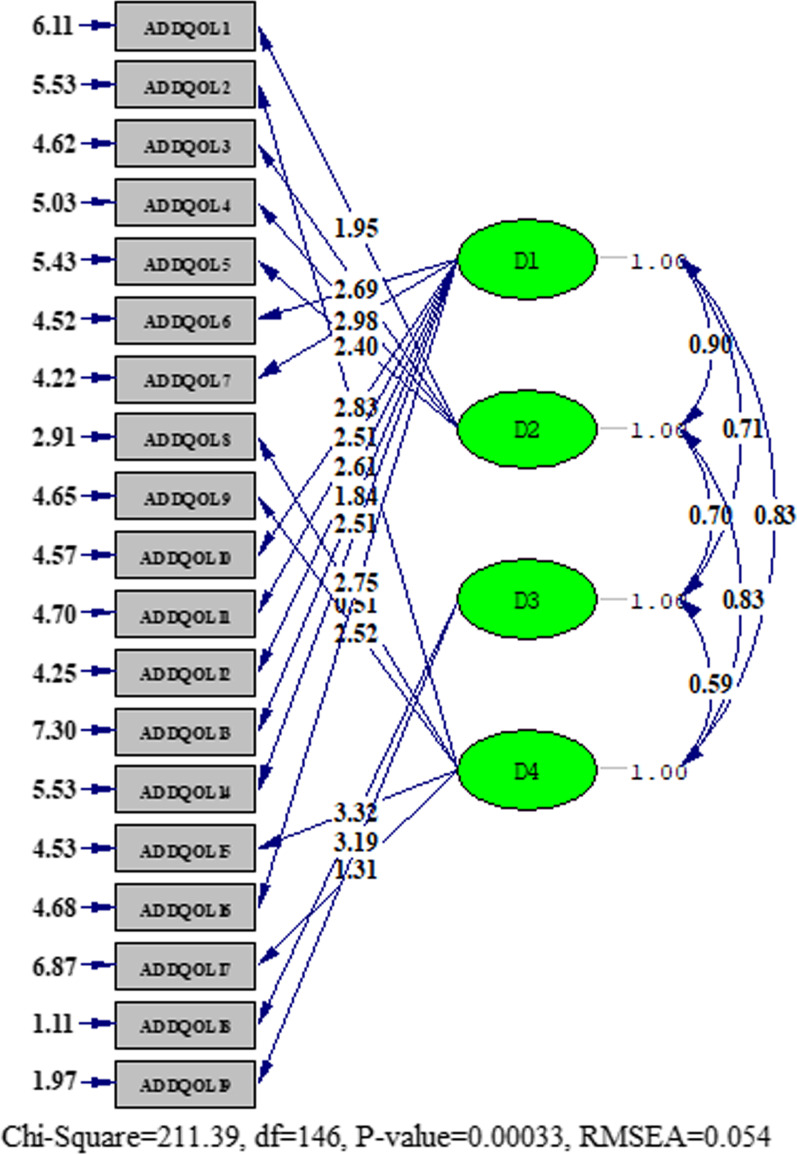
The results for four-factor model obtained from confirmatory factor analysis

*Bolds are items that belongs to a given factor

b. Confirmatory factor analysis (CFA): The results of confirmatory factor analysis for a one-factor structure showing the model fit are presented in Table 3. The results for one-factor confirmatory factor analysis is presented in Fig. 1. The four-factor structure was then tested with CFA. As shown in Table 3 the model fit indices were improved slightly. The results for the four-factor model are presented in Fig. 2.

**Table 3:** Findings from confirmatory factor analysis for the IR-ADDQoL

	x^2^/df	RMSEA	SRMR	CFI	NNFI
Model 1 (one-factor CFA)	2.18	0.088	0.066	0.97	0.96
Model 2 (four-factor CFA)	1.44	0.054	0.054 (0.08 or less)	0.99	0.99

*CFA*: Confirmatory factor analysis; *RMSEA*: Root mean score error of approximation, *SRMR*: Standardized root mean square residual, *CFI*: Comparative fit index, *NNFI*: Non-normed fit index (the Tucker–Lewis index)

Reliability: The Cronbach’s α coefficient was 0.939 for the 19-item ADDQoL (including the item on personal relationships) and 0.941 for 18-items (excluding the item on personal relationships) indicating excellent internal consistency. In a four-factor model, Cronbach’s alpha was 0.915 for factor 1, 0.819 for factor 2, 0.929 for factor 3 and 0.726 for factor 4. There was no improvement in α value when any item on the scale was eliminated.

### Overall generic and diabetes-dependent quality of life

**Fig. 3:**
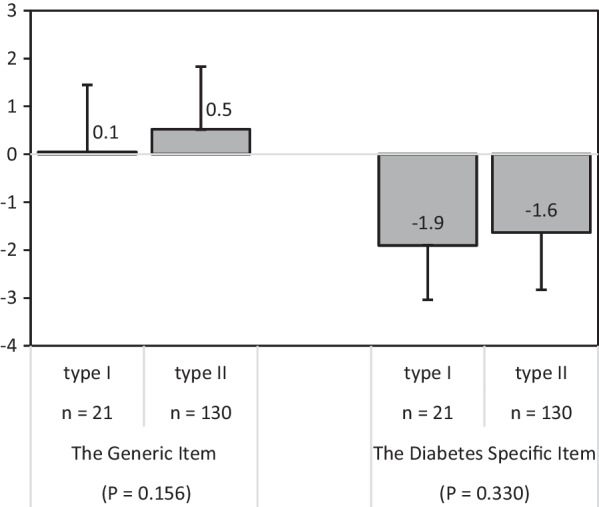
The findings for the generic and the diabetes-dependent overview items of IR-ADDQoL by type of diabetes (n = 151)

Figure 3 presents the mean response of diabetic patients to generic quality of life (GQOL) and diabetes-dependent quality of life (DDQOL) by type of diabetes. Compared to those with type 1 diabetes, those with type 2 diabetes appeared to report better quality of life (higher GQOL and less negative impact of diabetes on quality of life DDQOL): however, these were not significant differences.

### Distribution of domain scores

The distribution of responses for impact, and importance ratings; and average weighted impact scores for the 19 domains are presented in Table 4. Diabetes had the greatest negative impact on ‘freedom to eat’ ( − 1.81 ± 1.10) and the least negative impact on ‘closest personal relationship’ ( − 0.31 ± 0.79). The highest level of importance was for ‘family life’ (2.85 ± 0.43) and the lowest was for ‘people’s reaction’ (1.76 ± 1.27). The ADDQoL weighted impact scores ranged from − 0.77 to -4.57 ( − 3.37 ± 2.29); the most negative weighted impact scores appeared for the following items: ‘feelings about the future’ (mean = − 4.57), ‘freedom to eat’ (mean = − 4.44), and ‘motivation’ (mean = − 4.40). The least negative weighted impact score was for ‘closest personal relationship’ (mean = − 0.77). Of the five domain-specific items with a not applicable (NA) option, the most frequently not applicable item was ‘sex life’ (n = 8, 5.2%).

**Table 4:** Distribution of responses for impact rating, importance rating and weighted impact scores

Items	Impact rating	Importance rating	Weighted impact score
	Mean (SD)	Mean (SD)	Mean (SD)
Leisure activities	− 1.29 (1.10)	2.14 (0.97)	− 2.94 (3.08)
Working life*	− 0.80 (1.11)	2.29 (0.97)	− 2.03 (3.00)
Local or long-distance journeys	− 1.37 (1.15)	2.19 (1.01)	− 3.25 (3.31)
Holidays*	− 1.45 (1.20)	2.33 (0.99)	− 3.63 (3.58)
Physical health	− 1.54 (1.13)	2.59 (0.76)	− 4.09 (3.38)
Family life*	− 1.44 (1.16)	2.85 (0.43)	− 4.12 (3.45)
Friendship and social life	− 1.28 (1.21)	2.63 (0.70)	− 3.58 (3.61)
Closest personal relationship*	− 0.31 (0.79)	2.62 (0.73)	− 0.77 (2.17)
Sex life*	− 1.21 (1.16)	2.27 (1.08)	− 3.13 (3.32)
Physical appearance	− 1.63 (1.20)	2.54 (0.83)	− 4.32 (3.58)
Self-confidence	− 1.33 (1.15)	2.82 (0.51)	− 3.78 (3.41)
Motivation	− 1.56 (1.12)	2.80 (0.50)	− 4.40 (3.38)
People’s reaction	− 1.16 (1.18)	1.76 (1.27)	− 2.64 (3.29)
Feelings about the future	− 1.65 (1.13)	2.65 (0.74)	− 4.57 (3.47)
Financial situation	− 0.94 (1.13)	2.31 (0.92)	− 2.35 (3.20)
Living conditions	− 1.44 (1.19)	2.69 (0.62)	− 3.95 (3.52)
Dependence on others	− 0.73 (1.01)	2.63 (0.78)	− 1.95 (2.95)
Freedom to eat	− 1.81 (1.10)	2.07 (1.05)	− 4.44 (3.55)
Freedom to drink	− 1.69 (1.09)	1.96 (1.11)	− 4.07 (3.55)

*The item has ‘not applicable’ option for response category

### Quality of life by type of diabetes

**Fig. 4:**
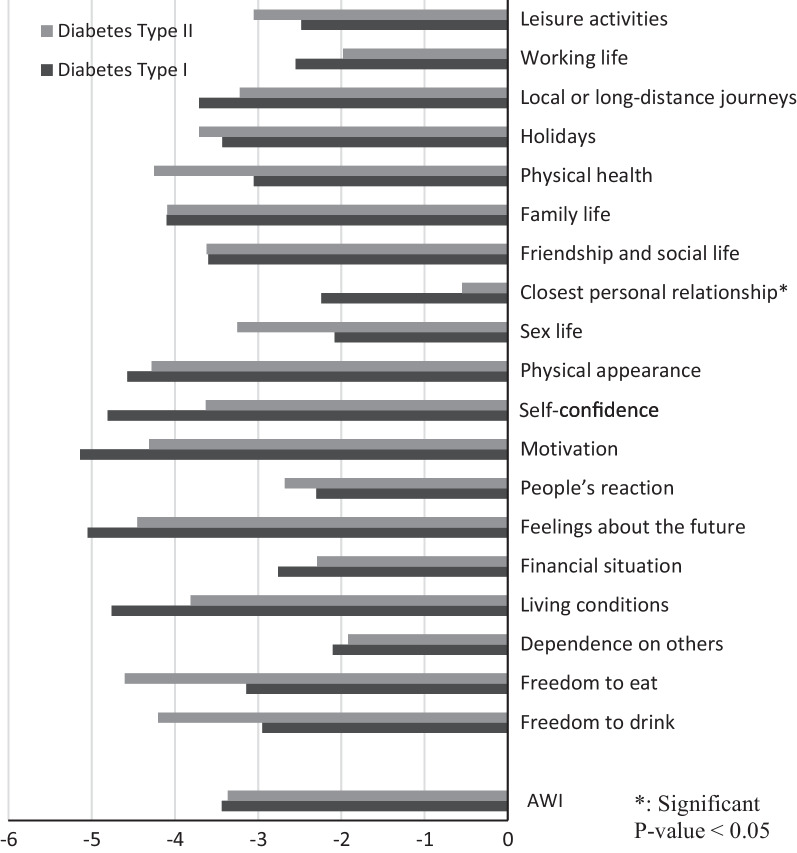
The findings of 19 domains of IR-ADDQOL and AWI by type of diabetes

Figure 4 presents the mean responses for the 19 specific domains of ADDQoL and AWI by type of diabetes. Compared to those with type 2 diabetes, those with type 1 diabetes reported worse QoL in 11 domains but the difference was statistically significant only for one item, closest personal relationship (P < 0.05).

## Discussion

The ADDQoL is a widely used diabetes-specific scale throughout the world. The present study was the first to develop and introduce the Persian version of ADDQoL in Iran. The exploratory and confirmatory factor analyses and Cronbach’s α all showed satisfactory results, implying that the scale was well translated and culturally adapted for Iranian patients.

On most domains, the impact of diabetes on quality of life was reported to be negative except for a very few patients who reported some positive effects. Similar observation was reported from studies conducted in the UK [26], and China [12]. The greatest negative unweighted impact was observed for ‘freedom to eat’ in our study which is very similar to studies conducted in Greece [27], Poland [28], Japan [13], Portugal [15], Taiwan [17], Slovenia [19], and Lithuania [20]. In this regard one might suggest that the appropriate education and support directed at good nutrition adjusted to the preferences of patients with diabetes might led to improved quality of life, and even to an improvement in their glycemic control [28]. For example, the Dose Adjustment for Normal Eating (DAFNE) study, with a flexible and intensive 5-day single course in the treatment of adults with type 1 diabetes with insulin, provided major long-term benefits for quality of life outcomes and treatment satisfaction. However, the authors of this study acknowledge that by structured follow-up and considering the factors involved in successful behavior change and maintenance, the short-term and long-term benefits of DAFNE for glycemic control may be maximized. Accordingly, health care providers can focus on the benefits of multiple daily injections and insulin pumps in treatment of patients with type 1 diabetes. Obviously, in this method, the active participation of the patient in the self-care process is essential [29, 30].

The highest importance score was attributed to ‘family life’. The same result of family having the highest importance rating was found in Singapore [31], China [12], Slovakia [32], and Greece [27]. This is logical because Iranian culture is very rich and the family is the main pillar of society in Iranian culture and is very valuable for them. Therefore, it is not surprising that the highest importance in the present study among the 19 domains of the questionnaire was assigned to family life.

The greatest diabetes weighted negative impact in the present study was indicated for ‘feelings about future’ which is consistent with the result obtained from Lithuania [20], Poland [28] and Taiwan [17]. In Iran, perhaps one reason for this could be attributed to the issue of access to medicine for glycemic control which originated from economic sanctions and the effects it has on access to medicine [33].

As shown in the results, The Persian ADDQoL had good psychometric properties. In the forced one-factor structure (which explained 48% of the total variance), the factor loadings were above 0.4 for all items except for personal relationship. This model also showed an acceptable internal consistency (Cronbach’s alpha = 0.94). This result was comparable to the one-factor solution explaining 44.3% of total variance in Japan [13], 48.9% in Italy [14] and 51.5% in Taiwan [17].

The unforced exploratory factor analysis revealed a four-factor solution which explained 66.53% of the total variance observed. All factor loadings exceeded 0.40 in the unforced factor solution indicating that all items had a satisfactory correlation with their corresponding factors. This result was comparable to the four-factor solution explaining 60.8% of total variance in Japan [13] and 53.35% in the Taiwan [17]. Also, the results obtained from exploratory factor analysis showed that 8 of 19 items loaded on the first factor, accounting for 48.87% of the variance. The second, third and fourth components with 4, 2 and 5 items, by contrast only accounted for 7.23%, 6.16% and 5.27% of the variance respectively.

Given the reliability and psychometric validity of the Persian version of ADDQoL, using this instrument in clinical settings specially ‘diabetes clinics’ would provide additional information for clinicians and might help to understand patients’ needs better. As the results, improvement of quality of life in diabetes patients would potentially be more achievable in a low cost and great benefits.

## Strengths and limitations

Despite the satisfactory results of the newly developed ADDQoL for Iranian adults with diabetes, we acknowledge several limitations of the current study: First, the respondents were recruited based on non-randomized sampling method. In addition, the proportion according to sex and between participants with diabetes 1 and 2 was not protected. Thus, due to this limitations, the results may not be generalizable to the whole population of adults with diabetes. Secondly, Iranian people are usually not so frank to talk about their sex life or private life because of their conservative culture. Thus, we think this might have led to bias in terms of importance and weighted impact score. Thirdly, the sample size was relatively small and it is suggested the scale be used with a larger sample in future studies. Finally, classical psychometrics rather than modern clinimetrics was used to evaluate the measurement properties of the Persian version of the Audit of Diabetes-Dependent Quality of Life questionnaire. This is a limitation of the present study implying that future research is needed to test the clinical validity of this evaluation method using clinimetric principles [34, 35].

## Conclusion

The Persian version of ADDQoL showed satisfactory reliability and acceptable validity. Therefore, it could be recommended for use and evaluation of quality of life in people with diabetes both in outcome studies and research settings in Iran.

## Supplementary Information

**Additional file 1**. ADDQoL Forward-translation Report

**Additional file 2**. ADDQoL Backward-translation Report

## Data Availability

The datasets used and/or analyzed during the current study are available from the corresponding author on reasonable request.

## Abbreviations

ADDQoL: Audit of Diabetes Dependent quality of life
GQoL: Generic quality of life
DDQoL: Diabetes-dependent quality of life
AWI: Average weighted impact
NA: Not applicable
HPR: Health Psychology Research
FT: Forward translation
BT: Back translation
CD: Cognitive debriefing
EFA: Exploratory factor analysis
CFA: Confirmatory factor analysis
DAFNE: Dose adjustment for normal eating

## References

[1] International Diabetes Federation (IDF) (2021). IDF Diabetes Atlas.

[2] Javanbakht M, Mashayekhi A, Baradaran HR, Haghdoost A, Afshin A (2015). Projection of diabetes population size and associated economic burden through 2030 in Iran: evidence from Micro-Simulation Markov Model and Bayesian meta-analysis. PLoS ONE.

[3] Krzemińska S, Bąk E, Šáteková L, Polanská A, Hašová K, Laurinc M (2020). Comparison of diabetes-dependent quality of life (ADDQoL) in patients with T2DM in Poland, The Czech Republic, and Slovakia. Diabetes Metab Syndr Obes.

[4] Oluchi SE, Manaf RA, Ismail S, Kadir Shahar H, Mahmud A, Udeani TK (2021). Health related quality of life measurements for diabetes: a systematic review. Int J Environ Res Public Health.

[5] Rodríguez-Almagro J, García-Manzanares Á, Lucendo AJ, Hernández-Martínez A (2018). Health-related quality of life in diabetes mellitus and its social, demographic and clinical determinants: a nationwide cross-sectional survey. J Clin Nurs.

[6] Efthymiadis A, Bourlaki M, Bastounis A (2022). The effectiveness of psychological interventions on mental health and quality of life in people living with type 1 diabetes: a systematic review and meta-analysis. Diabetol Int.

[7] Matlock KA, Broadley M, Hendrieckx C, Clowes M, Sutton A, Heller SR, de Galan BE, Pouwer F, Speight J, Hypo-RESOLVE consortium (2022). Changes in quality of life following hypoglycaemia in adults with type 2 diabetes: a systematic review of longitudinal studies. Diabet Med.

[8] Jensen MV, Broadley M, Speight J, Scope A, Preston L, Heller S, de Galan BE, Pouwer F, Hendrieckx C, Hypo-RESOLVE Consortium (2021). The impact of hypoglycaemia on the quality of life of family members of adults with type 1 or type 2 diabetes: a qualitative systematic review. Diabet Med.

[9] Carlton J, Leaviss J, Pouwer F, Hendrieckx C, Broadley MM, Clowes M, McCrimmon RJ, Heller SR, Speight J (2021). The suitability of patient-reported outcome measures used to assess the impact of hypoglycaemia on quality of life in people with diabetes: a systematic review using COSMIN methods. Diabetologia.

[10] Bradley C, Todd C, Gorton T, Symonds E, Martin A, Plowright R (1999). The development of an individualized questionnaire measure of perceived impact of diabetes on quality of life: the ADDQoL. Qual Life Res.

[11] Singh H, Bradley C (2006). Quality of life in diabetes. Int J Diab Dev Ctries.

[12] Kong D, Ding Y, Zuo X, Su W, Xiu L, Lin M (2011). Adaptation of the Audit of Diabetes-Dependent Quality of Life questionnaire to people with diabetes in China. Diabetes Res Clin Pract.

[13] Hirose AS, Fujihara K, Miyamasu F, Iwakabe S, Shimpo M, Heianza Y (2016). Development and evaluation of the Japanese version of the Audit of Diabetes-Dependent Quality of Life for patients with diabetes. Diabetol Int.

[14] Abbatecola AM, Spazzafumo L, Fabbietti P, Testa R, Rabini RA, Bonfigli AR (2015). Educational and psychological issues diabetes-related quality of life is enhanced by glycaemic improvement in older people. Diabetic Med.

[15] Costa FA, Guerreiro JP, Duggan C (2006). An Audit of Diabetes Dependent Quality of Life (ADDQoL) for Portugal: exploring validity and reliability. Pharm Pract (Granada).

[16] Bak E, Marcisz C, Nowak-Kapusta Z, Dobrzyn-Matusiak D, Marcisz E, Krzeminska S (2018). Psychometric properties of the Audit of Diabetes-Dependent Quality of Life (ADDQoL) in a population-based sample of Polish adults with type 1 and 2 diabetes. Health Qual Life Outcomes.

[17] Wang HF, Bradley C, Chang TJ, Chuang LM, Yeh MC (2017). Assessing the impact of diabetes on quality of life: validation of the Chinese version of the 19-item Audit of Diabetes-Dependent Quality of Life for Taiwan. Int J Qual Health Care.

[18] Soon SS, Goh SY, Bee YM, Poon JL, Li SC, Thumboo J, Wee HL (2010). Audit of Diabetes-Dependent Quality of Life (ADDQoL) [Chinese Version for Singapore] questionnaire: reliability and validity among Singaporeans with type 2 diabetes mellitus. Appl Health Econ Health Policy.

[19] Turk E, Prevolnik Rupel V, Tapajner A, Isola A (2014). Reliability and validity of the audit on diabetes-dependent quality of life (ADDQOL) and EQ-5D in elderly Slovenian diabetes mellitus type 2 patients. Health.

[20] Visockiene Z, Narkauskaite-Nedzinskiene L, Puronaite R, Mikaliukstiene A (2018). Validation of the Lithuanian version of the 19-item audit of diabetes dependent quality of life (ADDQoL-LI) questionnaire in patients with diabetes. Health Qual Life Outcomes.

[21] Ostini R, Dower J, Donald M (2012). The audit of diabetes-dependent quality of life 19 (ADDQoL): feasibility, reliability and validity in a population-based sample of Australian adults. Qual Life Res.

[22] Iversen MM, Espehaug B, Rokne B, Haugstvedt A, Graue M (2013). Psychometric properties of the Norwegian version of the audit of diabetes-dependent quality of life. Qual Life Res.

[23] Acquadro C, Conway K, Giroudet C, Mear I (2004). Lingustic manual for patient-reported outcomes (PRO) instruments.

[24] Hu L, Bentler PM (1999). Cutoff criteria for fit indexes in covariance structure analysis: conventional criteria versus new alternatives. Struct Equ Modeling.

[25] Brown TA (2015). Confirmatory factor analysis for applied research.

[26] Bradley C, Speight J (2002). Patient perceptions of diabetes and diabetes therapy: assessing quality of life. Diabetes Metab Res Rev.

[27] Papazafiropoulou AK, Bakomitrou F, Trikallinou A, Ganotopoulou A, Verras C, Christofilidis G (2015). Diabetes-dependent quality of life (ADDQOL) and affecting factors in patients with diabetes mellitus type 2 in Greece. BMC Res Notes.

[28] Bąk E, Nowak-Kapusta Z, Dobrzyn-Matusiak D, Marcisz-Dyla E, Marcisz C, Krzemińska SA (2019). An assessment of diabetes-dependent quality of life (ADDQoL) in women and men in Poland with type 1 and type 2 diabetes. Ann Agric Environ Med.

[29] DAFNE Study Group (2002). Training in flexible, intensive insulin management to enable dietary freedom in people with type 1 diabetes: dose adjustment for normal eating (DAFNE) randomised controlled trial. BMJ.

[30] Speight J, Amiel SA, Bradley C, Heller S, Oliver L, Roberts S, Rogers H, Taylor C, Thompson G (2010). Long-term biomedical and psychosocial outcomes following DAFNE (Dose Adjustment For Normal Eating) structured education to promote intensive insulin therapy in adults with sub-optimally controlled Type 1 diabetes. Diabetes Res Clin Pract.

[31] Shim YT, Lee J, Toh MP, Tang WE, Ko Y (2012). Health-related quality of life and glycaemic control in patients with type 2 diabetes mellitus in Singapore. Diabet Med.

[32] Holmanová E, Ziaková K (2009). Audit diabetes-dependent quality of life questionnaire: usefulness in diabetes self-management education in the Slovak population. J Clin Nurs.

[33] Salamati P, Chaufan C (2019). The harsh effects of sanctions on Iranian health. Lancet.

[34] Carrozzino D, Patierno C, Guidi J, Montiel CB, Cao J, Charlson ME, Fava GA (2021). Clinimetric criteria for patient-reported outcome measures. Psychother Psychosom.

[35] Fleck MP, Carrozzino D, Fava GA (2019). The challenge of measurement in psychiatry: the lifetime accomplishments of Per Bech (1942–2018). Braz J Psychiatr.

