# Supplementary material for: Translation and psychometric properties of the Persian version of the Audit of Diabetes Dependent Quality of Life (IR-ADDQoL)
Source: Health Qual Life Outcomes. 2022 Nov 28;20:156. doi: 10.1186/s12955-022-02071-0 (PMC9703709; doi:10.1186/s12955-022-02071-0)
Supplement: Supplementary file 1 — Additional file 1. ADDQoL Forward-translation Report [file 12955_2022_2071_MOESM1_ESM.doc]

ADDQoL Forward-translation Report

General Notes

These notes are addressed to the person managing the translation process, as that is the person who is usually the one compiling the report. Since we ask you to produce all your reports at the end of the process, it may facilitate your reporting if we provide you with this forward translation template. Having the original English, plus initial forward translations, your reconciled FT and reasons for the decisions made all together on the one form facilitates easier, faster and more effective review, should you or any subsequent users need to go back later to see why an item may not be working very well. We would not usually be involved at the forward translation stage, but please send us this with your back-translation report.

In the report template below please insert:

- the two initial, parallel forward translations (and please send us the fonts you have used if they are not standard European language fonts);
- the reconciled forward translation;
- your comments on the forward translations, together with any questions you have for us.

You will see that we have put each item into a separate section (starting each section on a new page), with an identifier in the header, to make it easier to identify and locate different parts of the report.

*Once the phrase *If I did not have diabetes* has been translated in overview item II, this doesn’t need to be repeated for each item here in the forward-translation report, so items 1-19 can commence from the phrase after the comma.

*Similarly, once the Yes / No format has been translated in Item 2, this does not need to be repeated for items 4, 6, 8 and 9 in the back-translation report.

However, when it comes to the repetition of the part (a) and (b) sets of response options, these are repeated each time, as some languages need to be able to reflect the relevant gender for each item.

Language

Please enter the language in the footer.

Key to abbreviations & report format:

OE = Original English

FT-1 = Parallel forward translation #1

FT-2 = Parallel forward translation #2

FT-Rec = Reconciled forward translation.

Comments = Please give initials and date for each contributor (use of different colour font for each person also helps us to track the discussion easily).

Following the results of the two parallel forward translations and reconciliation into the “FT‑rec”, please comment on the reasons for your selection of wording for the FT-rec.

Details of translators

Please give details of all the translators involved:

| **Translator** | **Name** | **Occupation / qualifications / experience** | **Native (first) language** |
| --- | --- | --- | --- |
| Translation co-ordinator | Ali Montazeri | Professor | Persian |
| 1^st^ forward translator | Zeinab Ghazanfari | PhD student | Persian |
| 2^nd^ forward translator | Sakineh Goljarean | Assistant professor | Persian |
| Person reconciling the 2 forward translations | Ali Montazeri | Professor | Persian |
| 1^st^ back translator | Zeinab Abdollahi | Master of Science | Persian |
| 2^nd^ back translator | Will be indicated by Professor Bradley |  | Persian |

General comments on translation

| *Please insert your name or initials and the date of your comments, e.g.* RHUL (RP) 20-Jan-04 *and put this before your comments for each item.*  RHUL (AW+RP) 28-Aug-09: As a general comment, we can see that there must have been some debate about how to structure your items with respect to the verbs and whether to include them in the item (as in the OE and almost all other 90+ languages) or put them in with the responses. This situation has arisen before in just one or two languages, e.g. German. The German versions have all gone with the style that you have in FT1 and FT2, i.e. kept the *would be* in the item and then left a gap … to imply or indicate where the answer would go (i.e. the words in the response options). You forward translators have seemingly been ambivalent about this and in some places both have gone for the German style, i.e. kept the verb in the statement, and in other places one FT has gone for the German solution and the other has moved the verb into the response options. The German was tested extensively during the development work for another QoL questionnaire (for diabetic retinopathy) and the solution of having the … space left in the item to indicate where the response option would go was accepted well by the patients we interviewed and the system has worked fine in subsequent use.  Can you tell us what has been done in other questionnaires in Persian? The fact that FT1 and FT2 have done it this way, even if not in all items, suggests that the forward translators have seen it done like this before. If there are other questionnaires that have used the format used by the German version, then we would definitely prefer to go with that if it is possible. May be it is possible for some items but not others; please investigate and advise us.  We have so far avoided having the verb *(would be)* in the response options. However, please let us know what is the common practice in Persian for this situation.  AM-30-Sep-09. A sentence in Persian could take for instance the following formats:  A simple sentence: subject + object + verb. (e.g. I + a letter + wrote).  An advanced: subject + adverb (time) + object + verb. (e.g. I + yesterday + a letter + wrote).  A more advanced: subject + adverb (time) + adverb (place) + preposition + complement + object + verb. (e.g. I + yesterday + at home + for + my father + a letter + wrote).  As you see in all instances the verb comes at the end of a sentence. |
| --- |

| Item | p.1, Instruction 1 |
| --- | --- |
| **OE** | This questionnaire asks about your quality of life – in other words how good or bad you feel your life to be. |
| **FT1** | این پرسشنامه درباره کیفیت زندگی شما سوال می کند. بعبارت دیگر مقدار احساس خوب یا بدی که درباره زندگی خود دارید |
| **FT2** | این پرسشنامه مسئله کیفیت زندگی را مطرح می کند. به بیانی دیگر احساس خوب یا بدی که درباره زندگی خود دارید |
| **FT-rec** | این پرسشنامه به بررسی کیفیت زندگی شما می پردازد. بعبارت دیگر این پرسشنامه در پی آن است تا احساس خوب یا بد شما را درباره زندگی بررسی نماید. |
| **Comments** | Both (FT1 and FT2) were very similar. However, the FT-rec was a combination of both. For instance ‘asks’ was translated ‘about’ by FT1; and ‘issue’ by FT2. This was changed to word ‘investigates’ which in meaning in Persian is very similar to ‘asks’.  RHUL (AW+RP) 28-Aug-09:🗸 |

| Item | p.1, Instruction 2 |
| --- | --- |
| **OE** | Please put an “X” in the box that best indicates your response for each item |
| **FT1** | لطفا در داخل مربع کنار پاسخی که بیشترین مطابقت را با پاسخ شما داردعلامت × بگذارید. |
| **FT2** | پاسخ خود را با علامت × مشخص کنید/ پاسخ هر سوال را با قرار دادن علامت × در مربع جلوی آن مشخص کنید. |
| **FT-rec** | لطفا پاسخ خود را با علامت × مشخص کنید. |
| **Comments** | Both (FT1 and FT2) translated the sentence word by word. However, the FT-rec was a simplified statement of both: Please indicate your response by ×.  RHUL (AW+RP) 28-Aug-09: Please could you retain the full sentence, in particular please include the phrase …*for each item*.  This was changed to:  AM-01-Sep-09. Please indicate your response for each item by ×. (translation of full sentence could cause confusion) |

| Item | p.1, Instruction 3 |
| --- | --- |
| **OE** | What we would like to know is how you feel about your life now. |
| **FT1** | ما دوست داریم چگونگی احساس شما را درباره زندگی فعلی تان بدانیم |
| **FT2** | دوست داریم احساس کنونی شما را درباره زندگی خود بدانیم |
| **FT-rec** | ما دوست داریم چگونگی احساس شما را درباره زندگی خود بدانیم. |
| **Comments** | No changes were made to the original statement.  RHUL (AW+RP) 28-Aug-09🗸 |

| Item | p.1, Overview item (I) |
| --- | --- |
| **OE** | In general, my present quality of life is: |
| **FT1** | در کل، کیفیت زندگی ام در حال حاضر ..... است. |
| **FT2** | در کل کیفیت زندگی من اکنون ..... است. |
| **FT-rec** | در مجموع، کیفیت زندگی من در حال حاضر ....... است. |
| **Comments** | No changes were made to the original statement.  RHUL (AW+RP) 28-Aug-09🗸 |

| Item | p.1, Overview item (I) responses |
| --- | --- |
| **OE** | excellent – very good – good – neither good nor bad – bad – very bad – extremely bad |
| **FT1** | عالی، بسیار خوب، خوب، نه خوب نه بد، بد، بسیار بد، بی نهایت بد |
| **FT2** | عالی، خیلی خوب، خوب، نه خوب نه بد، بد، خیلی بد، فوق العاده بد |
| **FT-rec** | عالی، بسیار خوب، خوب، نه خوب نه بد، بد، بسیار بد، بسیار بسیار بد |
| **Comments** | FT-rec now is reflecting the original response categories.  RHUL (AW+RP) 28-Aug-09: Please could you separate the responses as in the original English.  AM-28-Sep-09. Done. |

| Item | p.1, Instruction 4 (to overview Item II) |
| --- | --- |
| **OE** | Now we would like to know how your quality of life is affected by your diabetes, its management ****(including medication, visits to the doctor, and food)* and any complications you may have. |
| **FT1** | حالا ما می خواهیم بدانیم دیابت و مدیریت آن (شامل دارو، معاینه و پزشک) و هر عارضه ای که ممکن است داشته باشید، چه مقدار بر کیفیت زندگی شما تاثیر گذاشته است؟ |
| **FT2** | اکنون دوست داریم بدانیم دیابت، مدیریت دیابت (شامل دارو، معاینه و پزشک) و عوارض احتمالی آن چگونه بر کیفیت زندگی شما تاثیر گذاشته اند؟ |
| **FT-rec** | اکنون ما تمایل داریم بدانیم دیابت، درمان آن (شامل معاینات دوره ای، دستورهای داروئی و رژیم غذائی) و عوارض این بیماری چگونه بر کیفیت زندگی شما تاثیر گذاشته است. |
| **Comments** | The only problem with this item was misunderstanding of the translators about ‘management’. This was corrected. The word we used now is ‘treatment’.  RHUL (AW+RP) 28-Aug-09: Thank you – yes, ‘management’ can be difficult. But your solution of *treatment* + the extra bit in brackets should hopefully work well and your FT-rec for the examples in brackets looks rather more comprehensive than FT1 & 2.  AM-28-Sep-09. The Ft1 & 2 translations in brackets were incomplete. The Ft-rec is now exact translation of the original English. |

*** Please ensure that you have translated the section in brackets in case you encounter any problems later during the CDs. You may need to include this in your CDs (perhaps as an Alternative), in case you find the word you have had to use in your language for *management* doesn’t have a sufficiently broad meaning compared with the OE. If you haven’t done this right from the start, any wording needed for this won’t have been through the full process. Please also ensure that this is inserted where the phrase about *management* is repeated on the final page.

| Item | p.1, Overview Item (II) |
| --- | --- |
| **OE** | If I did not have diabetes, my quality of life would be: |
| **FT1** | اگر دیابت نداشتم، کیفیت زندگی من ..... بود. |
| **FT2** | اگر مبتلا به دیابت نبودم کیفیت زندگی ام ..... بود. |
| **FT-rec** | اگر دیابت نداشتم، کیفیت زندگی من .......... . |
| **Comments** | ‘Would be’ was included in response categories to make the item easier to be understood.  RHUL (AW+RP) 28-Aug-09: Is this because of the sentence construction in Persian? Please see our comments on this issue under General Comments at the beginning of the report.  AM-28-Sep-09. Yes. |

| Item | p.1, Overview Item (II) responses |
| --- | --- |
| **OE** | very much better – much better – a little better – the same – worse |
| **FT1** | خیلی بهتر، بهتر، کمی بهتر، فرقی نمی کرد، بدتر |
| **FT2** | بی نهایت بهتر،خیلی بهتر، کمی بهتر، یکسان، بدتر |
| **FT-rec** | خیلی بهتر می شد ، بهتر می شد، کمی بهتر می شد، فرقی نمی کرد، بدتر می شد |
| **Comments** | Please see the above comment.  RHUL (AW+RP) 28-Aug-09: Please could you separate the categories and show where ‘would be’ is placed, if it is finally decided that we need to do it this way, but see General Comments about this.  AM-28-Sep-09. Done. Would be is underlined:  خیلی بهتر می شد ، بهتر می شد، کمی بهتر می شد، فرقی نمی کرد، بدتر می شد |

| Item | p.2, Instruction 1, sentence 1 |
| --- | --- |
| **OE** | Please respond to the more specific statements on the following pages. |
| **FT1** | لطفا به جملات اختصاصی تری که در صفحات بعدی آمده است، پاسخ دهید |
| **FT2** | لطفا به جملات تخصصی تر در صفحات زیر پاسخ دهید |
| **FT-rec** | لطفا به سوالات مخصوصی که در زیر آمده است، پاسخ دهید. |
| **Comments** | No changes were made.  RHUL (AW+RP) 28-Aug-09: When you say ‘No changes were made’, we think you must mean that your FT-rec means the same as the OE. Please let us know.  AM-28-Sep-09. Yes it is true.  You seem to have made changes to both the FT1 & FT2 in order to arrive at the FT-rec. We wait to see from the BTs how this has worked out.  AM-28-Sep-09. The wording is a bit different but the meanings are the same. FT-rec. is much easier to read and understand. |

| Item | p.2, Instruction 1, sentence 2 |
| --- | --- |
| **OE** | For each aspect of life described, you will find two parts: |
| **FT1** | شما برای توصیف هر جنبه از زندگی دو قسمت مشاهده خواهید کرد |
| **FT2** | برای توصیف هر جنبه از زندگی دو بخش خواهید یافت |
| **FT-rec** | برای توصیف هر جنبه از زندگی دو قسمت مشاهده خواهید کرد. |
| **Comments** | No changes were made.  RHUL (AW+RP) 28-Aug-09: You seem to have made changes to both the FT1 & FT2 in order to arrive at the FT-rec. We wait to see from the BTs how this has worked out. |

| Item | p.2 instruction box, part (a) instruction |
| --- | --- |
| **OE** | For Part (a): put an “X” in one box to show how diabetes affects this aspect of your life; |
| **FT1** | قسمت الف: با علامت زدن در داخل یکی از مربع های جلوی هر سوال میزان تاثیر دیابت را بر آن جنبه از زندگی خود نشان دهید. |
| **FT2** | بخش الف: با گذاشتن علامت × در یکی از مربع ها چگونگی تاثیر دیابت را بر این جنبه از زندگی نشان دهید |
| **FT-rec** | بخش الف- با زدن علامت × در یکی از مربع ها نشان دهید دیابت چگونه بر این جنبه از زندگی شما تاثیر گذاشته است |
| **Comments** | Ft2 was selected. However, the word ‘put an × sign’ was changed to ‘put an ×’.  RHUL (AW+RP) 28-Aug-09: Thank you for explaining. We will see how this works in the CDs. |

| Item | p.2 instruction box, part (b) instruction |
| --- | --- |
| **OE** | For Part (b): put an “X” in one box to show how important this aspect of your life is to your quality of life. |
| **FT1** | قسمت ب: با علامت زدن در داخل یکی از مربع های جلوی هر سوال میزان اهمیت این جنبه از زندگی خود را نشان دهید. |
| **FT2** | بخش ب: با گذاشتن علامت × در یکی از مربع ها میزان اهمیت این جنبه از زندگی خود را نشان دهید. |
| **FT-rec** | بخش ب- با زدن علامت × در یکی از مربع ها اهمیت هر جنبه از زندگی خود را نشان دهید |
| **Comments** | Ft2 was selected. However, the word ‘put an × sign’ was changed to ‘put an ×’.  RHUL (AW+RP) 28-Aug-09:🗸 |

| Item | (1a) statement |
| --- | --- |
| **OE** | …I would enjoy my leisure activities |
| **FT1** | لذتی که از فعالیت های تفریحی ام می بردم، ..... بود. |
| **FT2** | از فعالیت های تفریحی ...... لذت می بردم |
| **FT-rec** | از فعالیت های تفریحی ......... . |
| **Comments** | ‘Would be’ and ‘enjoy’ was included in response categories to make the item easier to be understood.  RHUL (AW+RP) 28-Aug-09: Please see our earlier comments about the different ways of doing this. Whatever you find out, when you research what has been done for other questionnaires that already exist in Persian, it may be that you will need to test both this format and the other kind of format used by FT1 & FT2 when you do the CDs, to see which format the patients prefer.  AM-28-Sep-09. Perhaps we will assess both formats as suggested. |

| Item | (1a) responses |
| --- | --- |
| **OE** | very much more – much more – a little more – the same – less |
| **FT1** | خیلی بیشتر، بیشتر، کمی بیشتر، فرقی نمی کرد، کمتر |
| **FT2** | بی نهایت بیشتر، خیلی بیشتر، نه بیشتر نه کمتر، کمتر |
| **FT-rec** | خیلی بیشتر لذت می بردم ، بیشتر لذت می بردم ، کمی بیشتر لذت می بردم ، فرقی نمی کرد، کمتر لذت می بردم |
| **Comments** | Please see the above comment.  RHUL (AW+RP) 28-Aug-09: Is it possible to show the responses by breaking them up with hyphens. Unless it is very difficult to understand, we would prefer to keep to the original sentence construction. We assume that the much longer responses shown in your FT-rec reflect your suggested method of having the verb in the response options. Before this is finally agreed, you will need to look into this (as discussed above).  AM-28-Sep-09. In practice this would be happen. |

| Item | (1b) statement |
| --- | --- |
| **OE** | My leisure activities are |
| **FT1** | فعالیت های تفریحی ام ..... هستند |
| **FT2** | فعالیت های تفریحی برای من ...... . |
| **FT-rec** | فعالیت های تفریحی برای من ....... . |
| **Comments** | No changes were made.  RHUL (AW+RP) 28-Aug-09: It looks as if you decided to go with FT2? FT2 looks to have gone with the kind of sentence construction that removes the verb from the item and puts it in the responses. Please see our comments on this issue under General Comments at the beginning of the report.  AM-28-Sep-09. Yes, you are right. |

| Item | (1b) responses |
| --- | --- |
| **OE** | very important – important – somewhat important – not at all important |
| **FT1** | خیلی مهم، مهم، کمی مهم، کاملا بی اهمیت |
| **FT2** | خیلی مهم هستند، مهم هستند، تا حدی مهم هستند، اصلا مهم نیستند |
| **FT-rec** | خیلی مهم هستند، مهم هستند، تا حدی مهم هستند، اصلا مهم نیستند |
| **Comments** | No changes were made.  RHUL (AW+RP) 28-Aug-09: Please could you show the categories by inserting hyphens.  Your choice of FT2 again seems to confirm our interpretation above for 1b, i.e. that you decided to put the verb in the responses, thereby making them much longer (if it is finally decided that we need to do it this way, but see General Comments about this). Please confirm we have understood this correctly.  AM-28-Sep-09. Yes, it is correct. |

| Item | (2) preliminary question |
| --- | --- |
| **OE** | Are you currently working, looking for work or would you like to work? |
| **FT1** | آیا شما در حال حاضر کار می کنید یا در جستجوی کار هستید یا دوست دارید کار کنید؟ |
| **FT2** | آیا در حال حاضر مشغول به کار هستید؟ یا در جستجوی کار هستید؟ یا علاقمند به کار کردن هستید؟ |
| **FT-rec** | آیا در حال حاضر مشغول به کار هستید؟ یا در جستجوی کار هستید؟ یا علاقمند به کار کردن هستید؟ |
| **Comments** | No changes were made.  RHUL (AW+RP) 28-Aug-09: Please could you tell us what it is in the wording for FT2 that you think is better than FT1, causing you to choose FT2’s wording for the FT-Rec?  AM-28-Sep-09. FT1 used two different verbs for working while FT2 used similar verbs that I thought is a better translation. |

| Item | (2) Yes/No answer options |
| --- | --- |
| **OE** | Yes …… If *yes*, complete (a) and (b)  No …… If *no*, go straight to 3a |
| **FT1** | اگر پاسخ شما بلی است، به قسمت الف و ب و در صورتی که پاسخ شما خیر است به قسمت الف از سوال 3 بروید |
| **FT2** | اگر پاسخ شما مثبت است گزینه های الف و ب را تکمیل نمائید. در غیر اینصورت مستقیما به قسمت الف سوال 3 بروید. |
| **FT-rec** | اگر پاسخ شما مثبت است گزینه های الف و ب را تکمیل نمائید. در غیر اینصورت مستقیما به قسمت الف سوال 3 بروید. |
| **Comments** | No changes were made.  RHUL (AW+RP) 28-Aug-09: Please could you tell us why FT2 is better than FT1 (since you have chosen the FT2 wording for your FT-rec)?  AM-30-Sep-09. Respondents would understand it much better. |

| Item | (2a) statement |
| --- | --- |
| **OE** | …my working life would be: |
| **FT1** | بخش کاری زندگی من ..... بود. |
| **FT2** | زندگی کاری ام ..... بود. |
| **FT-rec** | وضعیت کاری من ........ . |
| **Comments** | ‘Would be’ was included in response categories to make the item easier to be understood.  RHUL (AW+RP) 28-Aug-09: Please see our comments on this issue under General Comments at the beginning of the report. |

| Item | (2a) responses |
| --- | --- |
| **OE** | very much better – much better – a little better – the same – worse |
| **FT1** | خیلی بهتر، بهتر، کمی بهتر، فرقی نمی کرد، بدتر |
| **FT2** | بی نهایت بهتر،خیلی بهتر، کمی بهتر، نه بهتر نه بدتر، بدتر |
| **FT-rec** | خیلی بهتر می شد، بهتر می شد، کمی بهتر می شد، فرقی نمی کرد، بدتر می شد |
| **Comments** | Please see the above comment.  RHUL (AW+RP) 28-Aug-09: Please see our comments for 1 (a) statement and responses. |

| Item | (2b) statement |
| --- | --- |
| **OE** | For me, having a working life is: |
| **FT1** | داشتن کار در زندگی برای من ..... است. |
| **FT2** | زندگی کاری برای من ..... است. |
| **FT-rec** | برای من داشتن کار ......... . |
| **Comments** | The verb ‘is’ was not included in the item.  RHUL (AW+RP) 28-Aug-09. Your FT-rec is shorter than both FT1 and FT2. Can you confirm that the *have a …* is included in the Persian? As you will see from the Concept Translation Guidelines (CTGs) the concept of *having* a working life is a key part of this item.  AM-30-Sep-09. Yes, it is included in response categories. |

| Item | (2b) responses |
| --- | --- |
| **OE** | very important – important – somewhat important – not at all important |
| **FT1** | خیلی مهم، مهم، کمی مهم، کاملا بی اهمیت |
| **FT2** | خیلی مهم، مهم، تا حدی مهم، کاملا بی اهمیت |
| **FT-rec** | خیلی مهم است، مهم است، تا حدی مهم است، اصلا مهم نیست |
| **Comments** | The verb ‘is’ was included in response categories.  RHUL (AW+RP) 28-Aug-09: See our previous comment above. Please could you separate the responses (probably best to do it as we have by using hyphens).  AM-30-Sep-09. In practice this would happen. |

| Item | (3a) statement |
| --- | --- |
| **OE** | … local or long distance journeys would be |
| **FT1** | طی کردن مسافت های کوتاه و بلند ..... می شد. |
| **FT2** | رفتن به جاهای دور و نزدیک ..... می شد. |
| **FT-rec** | رفتن به جاهای دور و نزدیک ..... . |
| **Comments** | ‘Would be’ war removed from the item.  RHUL (AW+RP) 28-Aug-09: Please see our earlier comments about the structure of the statements and responses. |

| Item | (3a) responses |
| --- | --- |
| **OE** | very much easier – much easier – a little easier – the same – more difficult |
| **FT1** | خیلی آسانتر، آسانتر، کمی آسانتر، فرقی نمی کرد، سخت تر |
| **FT2** | بی نهایت آسانتر، خیلی آسانتر، کمی آسانتر، نه آسانتر نه سخت تر، سخت تر |
| **FT-rec** | خیلی آسانتر می شد، آسانتر می شد، کمی آسانتر می شد، فرقی نمی کرد، سخت تر می شد |
| **Comments** | ‘Would be’ was added to response categories.  RHUL (AW+RP) 28-Aug-09: Please see our earlier comments about the structure of the statements and responses and please separate the responses so that we can identify the individual response options. |

| Item | (3b) statement |
| --- | --- |
| **OE** | For me, local or long distance journeys are |
| **FT1** | طی نمودن مسافت های کوتاه و بلند برای من ..... است. |
| **FT2** | برای من رفتن به جاهای دور و نزدیک .... است. |
| **FT-rec** | داشتن توانائی برای رفتن به جاهای دور و نزدیک ......... . |
| **Comments** | The verb ‘are’ was not included in the item  RHUL (AW+RP) 28-Aug-09: Please see our earlier comments about the structure of the statements and responses. |

| Item | (3b) responses |
| --- | --- |
| **OE** | very important – important – somewhat important – not at all important |
| **FT1** | خیلی مهم، مهم، کمی مهم، کاملا بی اهمیت |
| **FT2** | خیلی مهم، مهم، تا حدی مهم، کاملا بی اهمیت |
| **FT-rec** | خیلی مهم است، مهم است، تا حدی مهم است، اصلا مهم نیست |
| **Comments** | The verb ‘are’ was added to response categories.  RHUL (AW+RP) 28-Aug-09: Please see our earlier comments about the structure of the statements and responses. |

| Item | (4) preliminary question |
| --- | --- |
| **OE** | Do you ever go on holiday or want to go on holiday? |
| **FT1** | آیا اصلا به تعطیلات می روید یا دوست دارید که به تعطیلات بروید؟ |
| **FT2** | آیا تعطیلات جائی می روید یا دوست دارید جائی بروید؟ |
| **FT-rec** | آیا اصلا در تعطیلات به مسافرت می روید یا دوست دارید که به مسافرت بروید؟ |
| **Comments** | FT2 did not translate ‘ever’ but was better translated. However, FT1 did included ‘ever’ and the FT-rec was a combination of both.  RHUL (AW+RP) 28-Aug-09: 🗸 Thank you for explaining. |

| Item | (4a) statement |
| --- | --- |
| **OE** | … my holidays would be |
| **FT1** | گذراندن تعطیلاتم ..... بود |
| **FT2** | تعطیلاتم ..... سپری می شد. |
| **FT-rec** | مسافرتم ........ . |
| **Comments** | ‘Would be’ was not included in the item  RHUL (AW+RP) 28-Aug-09: Please see our earlier comments about the structure of the statements and responses. |

| Item | (4a) responses |
| --- | --- |
| **OE** | very much better – much better – a little better – the same – worse |
| **FT1** | خیلی بهتر، بهتر، کمی بهتر، فرقی نمی کرد، بدتر |
| **FT2** | بی نهایت بهتر،خیلی بهتر، کمی بهتر، نه بهتر نه بدتر، بدتر |
| **FT-rec** | خیلی بهتر می شد، بهتر می شد، کمی بهتر می شد، فرقی نمی کرد، بدتر می شد |
| **Comments** | ‘would be’ was added to response categories  RHUL (AW+RP) 28-Aug-09: Please see our earlier comments about the structure of the statements and responses. |

| Item | (4b) statement |
| --- | --- |
| **OE** | For me, holidays are |
| **FT1** | برای من، تعطیلات ..... است |
| **FT2** | تعطیلات برای من ..... است. |
| **FT-rec** | برای من رفتن به مسافرت در تعطیلات ........ . |
| **Comments** | The verb ‘are’ was not included in the item  RHUL (AW+RP) 28-Aug-09: Please see our earlier comments about the structure of the statements and responses. |

| Item | (4b) responses |
| --- | --- |
| **OE** | very important – important – somewhat important – not at all important |
| **FT1** | خیلی مهم، مهم، کمی مهم، کاملا بی اهمیت |
| **FT2** | خیلی مهم، مهم، تا حدی مهم، کاملا بی اهمیت |
| **FT-rec** | خیلی مهم است، مهم است، تا حدی مهم است، اصلا مهم نیست |
| **Comments** | The verb ‘are’ was added to response categories.  RHUL (AW+RP) 28-Aug-09: Please see our earlier comments about the structure of the statements and responses. |

l

| Item | (5a) statement |
| --- | --- |
| **OE** | … physically I could do |
| **FT1** | فعالیت هائی که می توانستم به لحاظ جسمی انجام دهم ..... بود. |
| **FT2** | می توانستم فعالیت های جسمی ..... را انجام دهم. |
| **FT-rec** | می توانستم به لحاظ جسمی فعالیت های ........ . |
| **Comments** | ‘Could do’ was removed from the item.  RHUL (AW+RP) 28-Aug-09: Please see our earlier comments about the structure of the statements and responses. |

| Item | (5a) responses |
| --- | --- |
| **OE** | very much more – much more – a little more – the same – less |
| **FT1** | خیلی بیشتر، بیشتر، کمی بیشتر، یکسان، کمتر |
| **FT2** | بی نهایت بیشتر، خیلی بیشتر، نه بیشتر نه کمتر، کمتر |
| **FT-rec** | خیلی بیشتری انجام دهم، بیشتری انجام دهم، کمی بیشتری انجام دهم، فرقی نمی کرد، کمتری انجام دهم |
| **Comments** | ‘Could do’ was added to response categories.  RHUL (AW+RP) 28-Aug-09: Please see our earlier comments about the structure of the statements and responses. |

| Item | (5b) statement |
| --- | --- |
| **OE** | For me, how much I can do physically is |
| **FT1** | مقدار کاری که بتوانم به لحاظ جسمی انجام دهم برایم ..... است. |
| **FT2** | مقدار فعالیتی که بتوانم به لحاظ جسمی انجام دهم برایم ..... است. |
| **FT-rec** | برای من مقدار کاری که به لحاظ جسمی انجام می دهم، ........ . |
| **Comments** | ‘Is’ was removed from the item.  RHUL (AW+RP) 28-Aug-09: Please see our earlier comments about the structure of the statements and responses. |

| Item | (5b) responses |
| --- | --- |
| **OE** | very important – important – somewhat important – not at all important |
| **FT1** | خیلی مهم، مهم، کمی مهم، کاملا بی اهمیت |
| **FT2** | خیلی مهم، مهم، تا حدی مهم، کاملا بی اهمیت |
| **FT-rec** | خیلی مهم است، مهم است، تا حدی مهم است، اصلا مهم نیست |
| **Comments** | ‘Is’ was added to response categories.  RHUL (AW+RP) 28-Aug-09: Please see our earlier comments about the structure of the statements and responses. |

| Item | (6) preliminary question |
| --- | --- |
| **OE** | Do you have any family / relatives? |
| **FT1** | آیا خانواده یا خویشاوندی دارید؟ |
| **FT2** | آیا خانواده یا خویشاوندی دارید؟ |
| **FT-rec** | آیا خانواده یا خویشاوندی دارید؟ |
| **Comments** | No changes were made.  RHUL (AW+RP) 28-Aug-09: 🗸 |

| Item | (6a) statement |
| --- | --- |
| **OE** | … my family life would be |
| **FT1** | زندگی خانوادگی من ..... بود. |
| **FT2** | زندگی خانوادگی من ....... می شد. |
| **FT-rec** | زندگی خانوادگی من ....... . |
| **Comments** | ‘Would be’ was deleted.  RHUL (AW+RP) 28-Aug-09: Please see our earlier comments about the structure of the statements and responses. |

| Item | (6a) responses |
| --- | --- |
| **OE** | very much better – much better – a little better – the same – worse |
| **FT1** | خیلی بهتر، بهتر، کمی بهتر، فرقی نمی کرد، بدتر |
| **FT2** | بی نهایت بهتر،خیلی بهتر، کمی بهتر، نه بهتر نه بدتر، بدتر |
| **FT-rec** | خیلی بهتر می شد، بهتر می شد، کمی بهتر می شد، فرقی نمی کرد، بدتر می شد |
| **Comments** | ‘Would be’ was added to response categories.  RHUL (AW+RP) 28-Aug-09: Please see our earlier comments about the structure of the statements and responses. |

| Item | (6b) statement |
| --- | --- |
| **OE** | My family life is |
| **FT1** | زندگی خانوادگی ام..... است. |
| **FT2** | زندگی خانوادگی برای من .... است. |
| **FT-rec** | زندگی خانوادگی برای من ........ . |
| **Comments** | ‘Is’ was deleted.  RHUL (AW+RP) 28-Aug-09: Please see our earlier comments about the structure of the statements and responses. |

| Item | (6b) responses |
| --- | --- |
| **OE** | very important – important – somewhat important – not at all important |
| **FT1** | خیلی مهم، مهم، کمی مهم، کاملا بی اهمیت |
| **FT2** | خیلی مهم، مهم، تا حدی مهم، کاملا بی اهمیت |
| **FT-rec** | خیلی مهم است، مهم است، تا حدی مهم است، اصلا مهم نیست |
| **Comments** | ‘Is’ was added to response categories.  RHUL (AW+RP) 28-Aug-09: Please see our earlier comments about the structure of the statements and responses. |

| Item | (7a) statement |
| --- | --- |
| **OE** | … my friendships and social life would be |
| **FT1** | روابط دوستانه و اجتماعی من ..... بود. |
| **FT2** | دوستی ها و روابط اجتماعی من ..... بود. |
| **FT-rec** | دوستی ها و روابط اجتماعی من ........ . |
| **Comments** | ‘Would be’ was deleted.  RHUL (AW+RP) 28-Aug-09: Please see our earlier comments about the structure of the statements and responses. |

| Item | (7a) responses |
| --- | --- |
| **OE** | very much better – much better – a little better – the same – worse |
| **FT1** | خیلی بهتر، بهتر، کمی بهتر، فرقی نمی کرد، بدتر |
| **FT2** | بی نهایت بهتر،خیلی بهتر، کمی بهتر، نه بهتر نه بدتر، بدتر |
| **FT-rec** | خیلی بهتر می شد، بهتر می شد، کمی بهتر می شد، فرقی نمی کرد، بدتر می شد |
| **Comments** | ‘Would be’ was added to response categories.  RHUL (AW+RP) 28-Aug-09: Please see our earlier comments about the structure of the statements and responses. |

| Item | (7b) statement |
| --- | --- |
| **OE** | My friendships and social life are |
| **FT1** | روابط دوستانه و اجتماعی برای من ..... است. |
| **FT2** | دوستی ها و روابط اجتماعی من ..... هستند. |
| **FT-rec** | دوستی ها و روابط اجتماعی برای من ....... . |
| **Comments** | ‘Are’ was deleted.  RHUL (AW+RP) 28-Aug-09: Please see our earlier comments about the structure of the statements and responses. |

| Item | (7b) responses |
| --- | --- |
| **OE** | very important – important – somewhat important – not at all important |
| **FT1** | خیلی مهم، مهم، کمی مهم، کاملا بی اهمیت |
| **FT2** | خیلی مهم، مهم، تا حدی مهم، کاملا بی اهمیت |
| **FT-rec** | خیلی مهم است، مهم است، تا حدی مهم است، اصلا مهم نیست |
| **Comments** | ‘Are’ was added to response categories.  RHUL (AW+RP) 28-Aug-09: Please see our earlier comments about the structure of the statements and responses. |

| Item | (8) preliminary question |
| --- | --- |
| **OE** | Do you have or would you like to have a close personal relationship (e.g. husband / wife, partner): |
| **FT1** | آیا رابطه شخصی نزدیکی (برای مثال با همسر یا رفیق تان) دارید؟ یا دوست دارید رابطه شخصی نزدیکی داشته باشید؟ |
| **FT2** | آیا دارای روابط شخصی صمیمانه (برای مثال با همسر یا رفیق تان) هستید یا متمایل به داشتن چنین رابطه ای هستید؟ |
| **FT-rec** | آیا دارای رابطه نزدیک با همسر خود هستید یا دوست دارید چنین رابطه ای داشته باشید؟ |
| **Comments** | Because of cultural issue ‘Partner’ was not included.  RHUL (AW+RP) 28-Aug-09: 🗸 Understood. Thank you for letting us know. Can you tell us whether you have kept the *(e.g. …)* in the brackets, or have you just said *(husband / wife)* in the brackets? Please confirm.  AM-30-Sep-09. We kept e.g. in the brackets. |

| Item | (8a) statement |
| --- | --- |
| **OE** | … my closest personal relationship would be |
| **FT1** | نزدیک ترین رابطه شخصی ام ..... می شد. |
| **FT2** | روابط شخصی صمیمانه ام ..... می شد. |
| **FT-rec** | این رابطه ....... . |
| **Comments** | ‘Would be’ was deleted and the item changed to ‘This relationship……  RHUL (AW+RP) 28-Aug-09: Please see my comments for 1 (a) statement and responses. Did either FT1 or FT2 maintain ‘closest personal relationship’? Keeping this phrase focuses the participant’s attention on ‘the close personal relationship’ mentioned in the preliminary question. However, given your need to omit *partner* from Q.8 for cultural reasons, we think that saying …*this relationship…* may work just as well in in your version.  AM-30-Sep-09. Thank you. Exactly true! |

| Item | (8a) responses |
| --- | --- |
| **OE** | very much better – much better – a little better – the same – worse |
| **FT1** | خیلی نزدیکتر می شد، نزدیکتر می شد، کمی نزدیکتر می شد، فرقی نمی کرد، کمی دورتر می شد |
| **FT2** | خیلی بهتر می شد، بهترمی شد، کمی بهتر می شد، فرقی نمی کرد، بدتر می شد |
| **FT-rec** | خیلی گرم تر می شد، گرم تر می شد، کمی گرم تر می شد، فرقی نمی کرد، کمی سردتر می شد |
| **Comments** | ‘Would be’ was added to response categories.  RHUL (AW+RP) 28-Aug-09: Please see our earlier comments about the structure of the statements and responses. |

| Item | (8b) statement |
| --- | --- |
| **OE** | For me, having a close personal relationship is |
| **FT1** | برای من داشتن یک رابطه فردی نزدیک ..... است. |
| **FT2** | برای من داشتن رابطه ای شخصی و صمیمانه ..... است. |
| **FT-rec** | برای من داشتن این رابطه ......... . |
| **Comments** | ‘Is’ was deleted and item was changed to ‘For me this relationship……..  RHUL (AW+RP) 28-Aug-09: Please see our earlier comments about the structure of the statements and responses.  An additional point: the new form of the statement does not mention ‘having’ a close relationship. We assume, therefore, that it has been excluded. It is important to include the *having a close personal relationship,* otherwise it could be interpreted as just the person’s opinion about that kind of relationship and not specifically what it means to the person to have such a relationship. If it is very difficult to say *having* with *a close personal…*, you could if necessary consider *…having this kind of relationship…* It might be worth doing it both ways and having both versions back-translated.  AM-30-Sep-09. We changed it to: having this kind of relationship… |

| Item | (8b) responses |
| --- | --- |
| **OE** | very important – important – somewhat important – not at all important |
| **FT1** | خیلی مهم، مهم، کمی مهم، کاملا بی اهمیت |
| **FT2** | خیلی مهم، مهم، تا حدی مهم، کاملا بی اهمیت |
| **FT-rec** | خیلی مهم است، مهم است، تا حدی مهم است، اصلا مهم نیست |
| **Comments** | ‘Is’ was added to response categories.  RHUL (AW+RP) 28-Aug-09: Please see our earlier comments about the structure of the statements and responses. |

| Item | (9) preliminary question |
| --- | --- |
| **OE** | Do you have or would you like to have a sex life? |
| **FT1** | آیا روابط زناشوئی دارید یا متمایل به داشتن رابطه زناشوئی هستید؟ |
| **FT2** | آیا رابطه جنسی دارید یا متمایل به برقراری رابطه جنسی هستید؟ |
| **FT-rec** | آیا شما روابط زناشوئی دارید و یا دوست دارید که چنین رابطه ای داشته باشید؟ |
| **Comments** | Because of cultural sensitivity the item was changed to: Do you have or would you like to have marital relationship?  RHUL (AW+RP) 28-Aug-09: Understood. However, can you please tell us if this means just the physical side of the relationship? If it applies to more, then it becomes a different item from all other languages and could potentially become just a duplicate of the previous item *(close personal relationship)*. This is about only the physical side of the relationship. Please review this item and advise.  AM-30-Sep-09. The translation exactly implies the physical side of the relationship. Please note that there two different words: relationship, marital relationship. The former refers to more general concept while the later implies sexual relationship. |

| Item | (9a) statement |
| --- | --- |
| **OE** | … my sex life would be |
| **FT1** | روابط زناشوئی ام ..... بود. |
| **FT2** | روابط جنسی ام ..... بود. |
| **FT-rec** | روابط زناشوئی من ....... . |
| **Comments** | ‘Would be’ was deleted and item was changed to My marital relationship……  RHUL (AW+RP) 28-Aug-09: Please see our comments immediately above for Item (9) and its potential similarity to Item 8. Also our earlier comments about the structure of the statements and responses.  AM-30-Sep-09. Please see the above explanation. |

| Item | (9a) responses |
| --- | --- |
| **OE** | very much better – much better – a little better – the same – worse |
| **FT1** | خیلی بهتر، بهتر، کمی بهتر، فرقی نمی کرد، بدتر |
| **FT2** | بی نهایت بهتر،خیلی بهتر، کمی بهتر، نه بهتر نه بدتر، بدتر |
| **FT-rec** | خیلی بهتر می شد، بهتر می شد، کمی بهتر می شد، فرقی نمی کرد، بدتر می شد |
| **Comments** | ‘Would be’ was added to response categories.  RHUL (AW+RP) 28-Aug-09: Please see our earlier comments about the structure of the statements and responses. |

| Item | (9b) statement |
| --- | --- |
| **OE** | For me, having a sex life is |
| **FT1** | داشتن رابطه زناشوئی برای من ..... است. |
| **FT2** | برای من داشتن رابطه جنسی..... است. |
| **FT-rec** | این رابطه برای من ....... . |
| **Comments** | ‘Is’ was deleted and the item was changed to: This relationship…..  RHUL (AW+RP) 28-Aug-09: Please see our earlier comments about the structure of the statements and responses. Please see our comments  ‘This relationship’ was used in 8 to refer to a ‘close personal relationship’. Therefore, if the meaning of the wording you have used for this item is too close to that of Item 8, we would now have a problem with using *“This relationship”*, as it would increase the risk of this item becoming a repetition of Item 8. You need to have wording here that will ensure that the patients are clear which kind (or part) of the relationship is being referred to.  AM-30-Sep-09- I think my above explanation could solve the problem! |

| Item | (9b) responses |
| --- | --- |
| **OE** | very important – important – somewhat important – not at all important |
| **FT1** | خیلی مهم، مهم، کمی مهم، کاملا بی اهمیت |
| **FT2** | خیلی مهم، مهم، تا حدی مهم، کاملا بی اهمیت |
| **FT-rec** | خیلی مهم است، مهم است، تا حدی مهم است، اصلا مهم نیست |
| **Comments** | ‘Is’ was added to response categories.  RHUL (AW+RP) 28-Aug-09: Please see our earlier comments about the structure of the statements and responses. |

| Item | (10a) statement |
| --- | --- |
| **OE** | …my physical appearance would be |
| **FT1** | ظاهر جسمی ام .... بود. |
| **FT2** | به لحاظ جسمی ظاهر ..... داشتم. |
| **FT-rec** | به لحاظ قیافه ....... . |
| **Comments** | ‘Would be’ was deleted. Physical was translated by both FT1 and FT2 very literally. A similar word was replaced to convey the actual meaning of the item.  RHUL (AW+RP) 28-Aug-09: Please see our earlier comments about the structure of the statements and responses.  Can you confirm that the way you have expressed *physical* does not suggest we are talking about a person’s “looks”, i.e. how handsome or unattractive they are? Please see the concept guidelines on this point.  AM-30-Sep-09. At present it means physical look! I think we should choose FT1’ or FT2’s translation! |

| Item | (10a) responses |
| --- | --- |
| **OE** | very much better – much better – a little better – the same – worse |
| **FT1** | خیلی بهتر می شدم، بهتر می شدم، کمی بهتر می شدم، فرقی نمی کردم، بدتر می شدم |
| **FT2** | بی نهایت بهتر می شدم، خیلی بهتر می شدم، کمی بهتر می شدم، فرقی نمی کردم، بدتر می شدم |
| **FT-rec** | خیلی بهتر به نظر می رسیدم، بهتر به نظر می رسیدم، کمی بهتر به نظر می رسیدم، فرقی نمی کردم، بدتر به نظر می رسیدم |
| **Comments** | ‘Would be’ was added to response categories.  RHUL (AW+RP) 28-Aug-09: Please see our earlier comments about the structure of the statements and responses. |

| Item | (10b) statement |
| --- | --- |
| **OE** | My physical appearance is |
| **FT1** | ظاهر جسمی برایم .... است. |
| **FT2** | به لحاظ جسمی ظاهرم ..... است. |
| **FT-rec** | قیافه و جلوه ظاهری برای من ....... . |
| **Comments** | ‘Is’ was deleted. Physical was translated by both FT1 and FT2 very literally. A similar word was replaced to convey the actual meaning of the item.  RHUL (AW+RP) 28-Aug-09: Please see our earlier comments about the structure of the statements and responses and our comments for 10a about the meaning of *physical appearance* in your translation.  AM-30-Sep-09. Similar explanation applies to this item! |

| Item | (10b) responses |
| --- | --- |
| **OE** | very important – important – somewhat important – not at all important |
| **FT1** | خیلی مهم، مهم، کمی مهم، کاملا بی اهمیت |
| **FT2** | خیلی مهم، مهم، تا حدی مهم، کاملا بی اهمیت |
| **FT-rec** | خیلی مهم است، مهم است، تا حدی مهم است، اصلا مهم نیست |
| **Comments** | ‘Is’ was added to response categories.  RHUL (AW+RP) 28-Aug-09: Please see our earlier comments about the structure of the statements and responses. |

| Item | (11a) statement |
| --- | --- |
| **OE** | … my self-confidence would be |
| **FT1** | اعتماد به نفس من ..... . |
| **FT2** | اعتماد به نفس ام ..... . |
| **FT-rec** | اعتماد به نفسم ..... |
| **Comments** | ‘Would be’ was deleted.  RHUL (AW+RP) 28-Aug-09: Please see our earlier comments about the structure of the statements and responses. |

| Item | (11a) responses |
| --- | --- |
| **OE** | very much better – much better – a little better – the same – worse |
| **FT1** | خیلی بهتر می شد، بهتر می شد، کمی بهتر می شد، فرقی نمی کرد، بدتر می شد |
| **FT2** | خیلی بیشتر می شد، بیشتر می شد، کمی بیشتر می شد، فرقی نمی کرد، کمتر می شد |
| **FT-rec** | خیلی بیشتر می شد، بیشتر می شد، کمی بیشتر می شد، فرقی نمی کرد، کمتر می شد |
| **Comments** | ‘Would be’ was added to response categories.  RHUL (AW+RP) 28-Aug-09: Please see our earlier comments about the structure of the statements and responses. |

| Item | (11b) statement |
| --- | --- |
| **OE** | My self-confidence is |
| **FT1** | داشتن اعتماد به نفس برای من .......... است. |
| **FT2** | اعتماد به نفس داشتن برایم .... است. |
| **FT-rec** | اعتماد به نفس برای من ....... . |
| **Comments** | ‘Is’ was deleted.  RHUL (AW+RP) 28-Aug-09: Please see our earlier comments about the structure of the statements and responses. |

| Item | (11b) responses |
| --- | --- |
| **OE** | very important – important – somewhat important – not at all important |
| **FT1** | خیلی مهم، مهم، کمی مهم، کاملا بی اهمیت |
| **FT2** | خیلی مهم، مهم، تا حدی مهم، کاملا بی اهمیت |
| **FT-rec** | خیلی مهم است، مهم است، تا حدی مهم است، اصلا مهم نیست |
| **Comments** | ‘Is’ was added to response categories.  RHUL (AW+RP) 28-Aug-09: Please see our earlier comments about the structure of the statements and responses. |

| Item | (12a) statement |
| --- | --- |
| **OE** | … my motivation would be |
| **FT1** | انگیزه ام ...... می شد. |
| **FT2** | انگیزه من ...... می شد. |
| **FT-rec** | انگیزه ام ..... . |
| **Comments** | ‘Would be’ was deleted  RHUL (AW+RP) 28-Aug-09: Please see our earlier comments about the structure of the statements and responses. Please see the CTGs. Was ‘motivation’ easy to translate? Some languages have a problem with this. They need to add something, e.g. *general motivation* or *motivation to do things*. Please review this and if you are in any doubt about whether this will be easily understood, please prepare an alternative and then we can add that for backtranslation and later you could test both with the patients. However, if this is fine as it is (i.e. as it is in the English).  AM-30-Sep-09. Motivation is to translate in one word! |

| Item | (12a) responses |
| --- | --- |
| **OE** | very much better – much better – a little better – the same – worse |
| **FT1** | خیلی بهتر، بهتر، کمی بهتر، نه بهتر نه بدتر، بدتر |
| **FT2** | خیلی بیشتر می شد، بیشتر می شد، کمی بیشتر می شد، فرقی نمی کرد، کمتر می شد |
| **FT-rec** | خیلی بیشتر می شد، بیشتر می شد، کمی بیشتر می شد، فرقی نمی کرد، کمتر می شد |
| **Comments** | ‘Would be’ was added to response categories.  RHUL (AW+RP) 28-Aug-09: Please see our earlier comments about the structure of the statements and responses. |

| Item | (12b) statement |
| --- | --- |
| **OE** | My motivation is |
| **FT1** | انگیزه برای من ....... است |
| **FT2** | انگیزه برایم ..... است. |
| **FT-rec** | داشتن انگیزه برایم ...... . |
| **Comments** | ‘Is’ was deleted.  RHUL (AW+RP) 28-Aug-09: Please see our earlier comments about the structure of the statements and responses (as well as the actual translation of *motivation*). |

| Item | (12b) responses |
| --- | --- |
| **OE** | very important – important – somewhat important – not at all important |
| **FT1** | خیلی مهم، مهم، کمی مهم، کاملا بی اهمیت |
| **FT2** | خیلی مهم، مهم، تا حدی مهم، کاملا بی اهمیت |
| **FT-rec** | خیلی مهم است، مهم است، تا حدی مهم است، اصلا مهم نیست |
| **Comments** | ‘Is’ was added to response categories.  RHUL (AW+RP) 28-Aug-09: Please see our earlier comments about the structure of the statements and responses. |

| Item | (13a) statement |
| --- | --- |
| **OE** | … the way people in general react to me would be |
| **FT1** | در کل روشی که مردم به من واکنش نشان می دادند، ..... می شد. |
| **FT2** | واکنش کلی افراد نسبت به من ..... می شد. |
| **FT-rec** | در کل، برخورد مردم نسبت به من ........ . |
| **Comments** | ‘Would be’ was deleted.  RHUL (AW+RP) 28-Aug-09: Please see our earlier comments about the structure of the statements and responses.  Can you please confirm to us that the *in general* qualifies the noun *people* and not the verb *react*? Please see the CTGs.  AM-30-Sep-30. Exactly. |

| Item | (13a) responses |
| --- | --- |
| **OE** | very much better – much better – a little better – the same – worse |
| **FT1** | خیلی بهتر، بهتر، کمی بهتر، فرقی نمی کرد، بدتر |
| **FT2** | بی نهایت بهتر،خیلی بهتر، کمی بهتر، نه بهتر نه بدتر، بدتر |
| **FT-rec** | خیلی بهتر می شد، بهتر می شد، کمی بهتر می شد، فرقی نمی کرد، بدتر می شد |
| **Comments** | ‘Would be’ was added to response categories.  RHUL (AW+RP) 28-Aug-09: Please see our earlier comments about the structure of the statements and responses. |

| Item | (13b) statement |
| --- | --- |
| **OE** | The way people in general react to me is |
| **FT1** | در کل روشی که مردم به من واکنش نشان می دهند، برایم ..... است. |
| **FT2** | چگونگی واکنش کلی افراد نسبت به من برایم ..... است. |
| **FT-rec** | چگونگی برخورد مردم نسبت به من برایم ....... . |
| **Comments** | ‘Is’ was deleted.  RHUL (AW+RP) 28-Aug-09: Please see our earlier comments about the structure of the statements and responses, and also our query about *in general* for 13a. |

| Item | (13b) responses |
| --- | --- |
| **OE** | very important – important – somewhat important – not at all important |
| **FT1** | خیلی مهم، مهم، کمی مهم، کاملا بی اهمیت |
| **FT2** | خیلی مهم، مهم، تا حدی مهم، کاملا بی اهمیت |
| **FT-rec** | خیلی مهم است، مهم است، تا حدی مهم است، اصلا مهم نیست |
| **Comments** | ‘Is’ was added to response categories.  RHUL (AW+RP) 28-Aug-09: Please see our earlier comments about the structure of the statements and responses. |

| Item | (14a) statement |
| --- | --- |
| **OE** | … my feelings about the future (e.g. worries, hopes) would be |
| **FT1** | احساساتم نسبت به آینده (مانند نگرانی ها و آرزوها) ..... بود. |
| **FT2** | احساساتم درباره آینده (مانند نگرانی ها و امیدها) ..... بود |
| **FT-rec** | احساساتم درباره آینده مثل احساس نگرانی یا امید به آینده ....... . |
| **Comments** | ‘Would be’ and parenthesis were deleted. However, examples were kept in the item.  RHUL (AW+RP) 28-Aug-09: Please see our earlier comments about the structure of the statements and responses. We notice that FT1 and FT2 kept the parenthesis. Could you please explain how removing the brackets improves the item? Do you feel it is easier to understand without them? Do you not use brackets in Persian the way that we do in European languages? We would prefer to keep them unless there is a strong reason for deleting them. Have you kept the *‘e.g….’* or have you deleted that too? It is important to retain the *e.g.*; *hopes and worries* may be the main feelings people will experience in relation to the future, but there may be other positive or negative feelings and the *e.g.* allows for this.  AM-30-Sep-09. e.g. and the words in the brackets already are included in the item. This reads much better than original English. |

| Item | (14a) responses |
| --- | --- |
| **OE** | very much better – much better – a little better – the same – worse |
| **FT1** | خیلی بهتر، بهتر، کمی بهتر، فرقی نمی کرد، بدتر |
| **FT2** | بی نهایت بهتر،خیلی بهتر، کمی بهتر، نه بهتر نه بدتر، بدتر |
| **FT-rec** | خیلی بهتر می شد، بهتر می شد، کمی بهتر می شد، فرقی نمی کرد، بدتر می شد |
| **Comments** | ‘Would be’ was added to response categories.  RHUL (AW+RP) 28-Aug-09: Please see our earlier comments about the structure of the statements and responses. |

| Item | (14b) statement |
| --- | --- |
| **OE** | My feelings about the future are |
| **FT1** | احساساتم نسبت به آینده برایم ..... هستند. |
| **FT2** | احساسی که نسبت به آینده دارم، برایم ..... است. |
| **FT-rec** | احساسی که نسبت به آینده دارم، برایم ....... . |
| **Comments** | ‘Are’ was deleted.  RHUL (AW+RP) 28-Aug-09: Please see our earlier comments about the structure of the statements and responses. |

| Item | (14b) responses |
| --- | --- |
| **OE** | very important – important – somewhat important – not at all important |
| **FT1** | خیلی مهم، مهم، کمی مهم، کاملا بی اهمیت |
| **FT2** | خیلی مهم، مهم، تا حدی مهم، کاملا بی اهمیت |
| **FT-rec** | خیلی مهم است، مهم است، تا حدی مهم است، اصلا مهم نیست |
| **Comments** | ‘Are’ was added to response categories.  RHUL (AW+RP) 28-Aug-09: Please see our earlier comments about the structure of the statements and responses. |

| Item | (15a) statement |
| --- | --- |
| **OE** | … my financial situation would be |
| **FT1** | وضع مالی من .... بود. |
| **FT2** | موقعیت اقتصادی ام ..... بود. |
| **FT-rec** | وضع مالی من ........ |
| **Comments** | ‘Would be’ was deleted.  RHUL (AW+RP) 28-Aug-09: Please see our earlier comments about the structure of the statements and responses. |

| Item | (15a) responses |
| --- | --- |
| **OE** | very much better – much better – a little better – the same – worse |
| **FT1** | خیلی بهتر، بهتر، کمی بهتر، فرقی نمی کرد، بدتر |
| **FT2** | بی نهایت بهتر،خیلی بهتر، کمی بهتر، نه بهتر نه بدتر، بدتر |
| **FT-rec** | خیلی بهتر می شد، بهتر می شد، کمی بهتر می شد، فرقی نمی کرد، بدتر می شد |
| **Comments** | ‘Would be’ was added to response categories.  RHUL (AW+RP) 28-Aug-09: Please see our earlier comments about the structure of the statements and responses. |

| Item | (15b) statement |
| --- | --- |
| **OE** | My financial situation is |
| **FT1** | وضع مالی برایم ..... است. |
| **FT2** | وضعیت اقتصادی برایم ..... است. |
| **FT-rec** | وضعیت مالی برایم ....... . |
| **Comments** | ‘Is’ was deleted.  RHUL (AW+RP) 28-Aug-09: Please see our earlier comments about the structure of the statements and responses. |

| Item | (15b) responses |
| --- | --- |
| **OE** | very important – important – somewhat important – not at all important |
| **FT1** | خیلی مهم، مهم، کمی مهم، کاملا بی اهمیت |
| **FT2** | خیلی مهم، مهم، تا حدی مهم، کاملا بی اهمیت |
| **FT-rec** | خیلی مهم است، مهم است، تا حدی مهم است، اصلا مهم نیست |
| **Comments** | ‘Is’ was added to response categories.  RHUL (AW+RP) 28-Aug-09: Please see our earlier comments about the structure of the statements and responses. |

| Item | (16a) statement |
| --- | --- |
| **OE** | … my living conditions would be |
| **FT1** | شرایط زندگی ام ..... می شد. |
| **FT2** | شرایط زندگی من ........ می شد. |
| **FT-rec** | شرایط زندگی من ....... . |
| **Comments** | ‘Would be’ was added to response categories.  RHUL (AW+RP) 28-Aug-09: Please see our earlier comments about the structure of the statements and responses. |

| Item | (16a) responses |
| --- | --- |
| **OE** | very much better – much better – a little better – the same – worse |
| **FT1** | خیلی بهتر، بهتر، کمی بهتر، فرقی نمی کرد، بدتر |
| **FT2** | بی نهایت بهتر،خیلی بهتر، کمی بهتر، نه بهتر نه بدتر، بدتر |
| **FT-rec** | خیلی بهتر می شد، بهتر می شد، کمی بهتر می شد، فرقی نمی کرد، بدتر می شد |
| **Comments** | ‘Would be’ was added to response categories.  RHUL (AW+RP) 28-Aug-09: Please see our earlier comments about the structure of the statements and responses. |

| Item | (16b) statement |
| --- | --- |
| **OE** | My living conditions are |
| **FT1** | شرایط زندگی برایم ..... است. |
| **FT2** | شرایط زندگی برای من ....... است. |
| **FT-rec** | شرایط زندگی برایم ....... . |
| **Comments** | ‘Are’ was added to response categories.  RHUL (AW+RP) 28-Aug-09: Please see our earlier comments about the structure of the statements and responses. |

| Item | (16b) responses |
| --- | --- |
| **OE** | very important – important – somewhat important – not at all important |
| **FT1** | خیلی مهم، مهم، کمی مهم، کاملا بی اهمیت |
| **FT2** | خیلی مهم، مهم، تا حدی مهم، کاملا بی اهمیت |
| **FT-rec** | خیلی مهم است، مهم است، تا حدی مهم است، اصلا مهم نیست |
| **Comments** | ‘Are’ was added to response categories.  RHUL (AW+RP) 28-Aug-09: Please see our earlier comments about the structure of the statements and responses. |

| Item | (17a) statement |
| --- | --- |
| **OE** | … I would have to depend on others when I do not want to |
| **FT1** | مجبور می شدم ..... به دیگران تکیه کنم، زمانی که تمایل نداشتم. |
| **FT2** | ......... زمانی که تمایل ندارم به دیگران تکیه کنم |
| **FT-rec** | وابستگی به دیگران زمانی که تمایل ندارم، ....... . |
| **Comments** | ‘Would have’ was removed from the item.  RHUL (AW+RP) 28-Aug-09: Please see our earlier comments about the structure of the statements and responses.  Can you please also confirm that the phrase *when I do not want to* is included in your FT-rec.  This item looks to have been more difficult than most to translate, in particular to decide where to allow for the ‘insertion’ of the verb.  AM-30-Sep-09. I confirm that at present FT-rec reads better and all words are there! |

| Item | (17a) responses |
| --- | --- |
| **OE** | very much less – much less – a little less – the same – more |
| **FT1** | بی نهایت کمتر، خیلی کمتر، کمی کمتر، یکسان (به یک اندازه)، بیشتر |
| **FT2** | خیلی کمتر مجبور می شدم، کمتر مجبور می شدم، کمی کمتر مجبور می شدم، فرقی نمی کرد، بیشتر مجبور می شدم |
| **FT-rec** | خیلی کمتر می شد، کمتر می شد، کمی کمتر می شد، فرقی نمی کرد، بیشتر می شد |
| **Comments** | ‘Would have’ was added to response categories.  RHUL (AW+RP) 28-Aug-09: Please see our earlier comments about the structure of the statements and responses. |

| Item | (17b) statement |
| --- | --- |
| **OE** | For me, not having to depend on others is |
| **FT1** | برای من ..... است که مجبور نباشم به دیگران وابسته باشم. |
| **FT2** | متکی نبودن به دیگران برایم ..... است. |
| **FT-rec** | مستقل بودن برای من ....... . |
| **Comments** | ‘Is’ was removed from the item.  RHUL (AW+RP) 28-Aug-09: Please see our earlier comments about the structure of the statements and responses. |

| Item | (17b) responses |
| --- | --- |
| **OE** | very important – important – somewhat important – not at all important |
| **FT1** | خیلی مهم، مهم، کمی مهم، کاملا بی اهمیت |
| **FT2** | خیلی مهم، مهم، تا حدی مهم، کاملا بی اهمیت |
| **FT-rec** | خیلی مهم است، مهم است، تا حدی مهم است، اصلا مهم نیست |
| **Comments** | ‘Is’ was added to response categories.  RHUL (AW+RP) 28-Aug-09: Please see our earlier comments about the structure of the statements and responses. |

| Item | (18a) statement |
| --- | --- |
| **OE** | … my freedom to eat as I wish would be |
| **FT1** | آزادی ام برای خوردن چیزهائی که دوست داشتم، ... می شد. |
| **FT2** | آزادی من در خوردن چیزهائی که دوست دارم، ..... می شد. |
| **FT-rec** | آزاد بودم هر آنچه را که می خواهم، بخورم. |
| **Comments** | ‘Would be’ was removed from the item.  RHUL (AW+RP) 28-Aug-09: Please see our earlier comments about the structure of the statements and responses.  Can you tell us if you have a direct translation for *as I wish*? If not, have you included (as indicated in the CTGs) *…to eat what I wish and when I wish would be*? These would both be needed in order to cover the full meaning of *as I wish*, which means both of these things.  AM-30-Sep-09. As wish was translated exactly and included in the translation. |

| Item | (18a) responses |
| --- | --- |
| **OE** | very much greater – much greater – a little greater – the same – less |
| **FT1** | بی نهایت بیشتر، خیلی بیشتر، کمی بیشتر، یکسان، کمتر |
| **FT2** | خیلی بیشتر، بیشتر، کمی بیشتر، فرقی نمی کرد، کمتر |
| **FT-rec** | خیلی آزاد بودم، آزاد بودم، کمی آزاد بودم، فرقی نمی کرد، آزاد نبودم |
| **Comments** | ‘Would be’ was added to response categories.  RHUL (AW+RP) 28-Aug-09: Please see our earlier comments about the structure of the statements and responses. |

| Item | (18b) statement |
| --- | --- |
| **OE** | My freedom to eat as I wish is |
| **FT1** | آزادی ام برای خوردن چیزهائی که دوست دارم، برایم ..... است. |
| **FT2** | آزاد بودن در خوردن چیزهائی که دوست دارم، برایم .... است. |
| **FT-rec** | آزاد بودن در خوردن چیزهائی که دوست دارم، برای من ..... |
| **Comments** | ‘Is’ was removed from the item.  RHUL (AW+RP) 28-Aug-09: Please see our earlier comments about the structure of the statements and responses, and also above for 18a (about the meaning of *as I wish*). |

| Item | (18b) responses |
| --- | --- |
| **OE** | very important – important – somewhat important – not at all important |
| **FT1** | خیلی مهم، مهم، کمی مهم، کاملا بی اهمیت |
| **FT2** | خیلی مهم، مهم، تا حدی مهم، کاملا بی اهمیت |
| **FT-rec** | خیلی مهم است، مهم است، تا حدی مهم است، اصلا مهم نیست |
| **Comments** | ‘Is’ was included in response categories.  RHUL (AW+RP) 28-Aug-09: Please see our earlier comments about the structure of the statements and responses. |

| Item | (19a) statement |
| --- | --- |
| **OE** | … my freedom to drink as I wish (e.g. fruit juice, alcohol, sweetened hot and cold drinks) would be |
| **FT1** | آزادی ام در نوشیدن چیزهائی که دوست دارم ( مثل آب میوه، نوشیدنی های شیرین سرد و گرم) ..... می شد. |
| **FT2** | آزادی من در نوشیدن چیزهائی که دوست دارم ( مثل آب میوه، نوشیدنی های شیرین سرد و گرم)، ..... می شد. |
| **FT-rec** | آزاد بودم هر آنچه را که می خواهم، بنوشم (مثل آب میوه و نوشیدنی های شیرین سرد و گرم) |
| **Comments** | ‘Would be’ was removed from the item.  RHUL (AW+RP) 28-Aug-09: Please see our earlier comments about the structure of the statements and responses.  Please also see our question in 18a about how you have translated *as I wish*. The same applies here in item 19a and b. |

| Item | (19a) responses |
| --- | --- |
| **OE** | very much greater – much greater – a little greater – the same – less |
| **FT1** | خیلی بیشتر، بیشتر، کمی بیشتر، نه بیشتر نه کمتر، کمتر |
| **FT2** | بی نهایت بیشتر، خیلی بیشتر، کمی بیشتر، نه بیشتر نه کمتر، کمتر |
| **FT-rec** | خیلی آزاد بودم، آزاد بودم، کمی آزاد بودم، فرقی نمی کرد، آزاد نبودم |
| **Comments** | ‘Would be’ was added to response categories.  RHUL (AW+RP) 28-Aug-09: Please see our earlier comments about the structure of the statements and responses. |

| Item | (19b) statement |
| --- | --- |
| **OE** | My freedom to drink as I wish is |
| **FT1** | آزادی در نوشیدن چیزهائی که دوست دارم برایم ..... است. |
| **FT2** | آزاد بودن در نوشیدن چیزهائی که دوست دارم برایم ........ . |
| **FT-rec** | آزاد بودن در نوشیدن چیزهائی که دوست دارم برای من ....... . |
| **Comments** | The word ‘is’ was removed from the item.  RHUL (AW+RP) 28-Aug-09: Please see our earlier comments about the structure of the statements and responses. And again the same applies here about the translation or *as I wish*. |

| Item | (19b) responses |
| --- | --- |
| **OE** | very important – important – somewhat important – not at all important |
| **FT1** | خیلی مهم، مهم، کمی مهم، کاملا بی اهمیت |
| **FT2** | خیلی مهم است، مهم است، تا حدی مهم است، اصلا مهم نیست |
| **FT-rec** | خیلی مهم است، مهم است، تا حدی مهم است، اصلا مهم نیست |
| **Comments** | ‘Is’ was added to response categories.  RHUL (AW+RP) 28-Aug-09: Please see my comments for 2 (b) statement and responses. |

| Item | Closing instruction |
| --- | --- |
| **OE** | If there are any other ways in which diabetes, its management*** and any complications affect your quality of life, please say what they are below: |
| **FT1** | اگر با مسائل دیگری در مدیریت بیماری دیابت خود و عوارض تاثیرگذار بر کیفیت زندگی خود مواجهید، در قسمت زیر لیست نمائید. |
| **FT2** | اگر با مسائل دیگری در درمان بیماری دیابت خود و عوارض تاثیرگذار بر کیفیت زندگی خود مواجهید، در قسمت زیر لیست نمائید. |
| **FT-rec** | اگر در خصوص دیابت، درمان و عوارض آن موضوعات دیگری وجود دارد که بر کیفیت زندگی شما اثر می گذارد، لطفا آنها را در قسمت زیر بنویسید. |
| **Comments** | Translators misunderstood ‘Management’. This was changed to treatment.  RHUL (AW+RP) 28-Aug-09: OK, thanks. Please see our comment earlier for Page 1 Instruction 4. However, we don’t see any brackets here. Have you left the brackets with examples out of the translation?  AM-30-Sep-09. No it is included in the item. |

Plus repetition of the additional explanatory phrase for *management*, as on p.1

| Item | Final “thank-you” |
| --- | --- |
| **OE** | Thank you for completing this questionnaire |
| **FT1** | از شما بخاطر تکمیل این پرسشنامه متشکریم |
| **FT2** | از تکمیل این پرسشنامه متشکریم. |
| **FT-rec** | از همکاری شما در تکمیل این پرسشنامه متشکریم |
| **Comments** | The word ‘your cooperation’ was added to the original statement.  RHUL (AW+RP) 28-Aug-09: 🗸 |

| Item | Page numbering |
| --- | --- |
| **OE** | Page x of y |
| **FT1** | صفحه x از y |
| **FT2** | صفحه x از y |
| **FT-rec** | صفحه x از y |
| **Comments** | No changes were made.  RHUL (AW+RP) 28-Aug-09: 🗸 Thanks. |
