# Supplementary material for: Translation and psychometric properties of the Persian version of the Audit of Diabetes Dependent Quality of Life (IR-ADDQoL)
Source: Health Qual Life Outcomes. 2022 Nov 28;20:156. doi: 10.1186/s12955-022-02071-0 (PMC9703709; doi:10.1186/s12955-022-02071-0)
Supplement: Supplementary file 2 — Additional file 2. ADDQoL Backward-translation Report [file 12955_2022_2071_MOESM2_ESM.doc]

ADDQoL Back-translation Report

General Notes

These notes are addressed to the person managing the translation process, as we would expect that person also to be the one compiling the report. Having the original English, plus forward and back-translations all together on the one form facilitates easier, faster and more effective review than having to refer to several different documents each time. When reporting on the back-translation and explaining choices and decisions, therefore, please include every part of every item, even if you think both forward and back-translations are fine and not in need of any comment (please note specific exceptions below*). Past experience has shown that problems can get missed if the forward and particularly both the back-translations aren’t available for reviewers to see. As the original authors, we can sometimes see problems that other people might not pick up and we may be able to propose alternative solutions.

In the report template below please insert:

• the reconciled forward translation (and please send us any special fonts we may not have);

• both the back-translations;

• your comments on the back-translations, together with any questions you have for us and comments on the reconciled forward translation; also any suggested revisions to the forward translation resulting from the back-translations (see sample format below).

You will see that we have put each item into a separate section (starting each section on a new page), with an identifier in the header, to make it easier to identify and locate different parts of the report.

*Once the phrase *If I did not have diabetes* has been translated in overview item II, this doesn’t need to be repeated for each item here in the back-translation report, so items 1-19 can commence from the phrase after the comma.

*Similarly, once the Yes / No format has been translated in Item 2, this does not need to be repeated for items 4, 6, 8 and 9 in the back-translation report.

However, when it comes to the repetition of the part (a) and (b) sets of response options, these are repeated each time, as some languages need to be able to reflect the relevant gender for each item.

Language

Please enter the language in the footer.

Key to abbreviations & report format:

OE = Original English

FT-Rec = Reconciled forward translation (where language script and fonts allow).

This would be the reconciled version from the two original forward translations. It is from this that the two back-translations would be carried out.

BT1 = One of two back-translations of the original FT-Rec

BT2 = Second of two back-translations of the original FT-Rec

Comments = Please give initials and date for each contributor (use of different colour font for each person also helps us to track the discussion easily).

FT rev1 = It is likely that, following the results of the back-translations, there will be some items in the FT-rec that you feel require changing before you send the report to us for review. You will find an additional section for this.

BT rev1 = Similarly, please provide a back translation of FT rev1.

Comments = You and we can then add comments and discussion on the revised FT.

Should you need subsequently to make further changes, simply add on a similar section for FT rev2, BT rev2 and Comments as shown here.

| **FT rev2** |  |
| --- | --- |
| **BT rev2** |  |
| **Comments** |  |

Details of translators

Please give details of all the translators involved:

| **Translator** | **Name** | **Occupation / qualifications / experience** | **Native (first) language** |
| --- | --- | --- | --- |
| Translation co-ordinator | Prof. Ali Montazeri | Professor | Persian |
| 1^st^ forward translator | Zeinab Ghazanfari | Assistant Professor | Persian |
| 2^nd^ forward translator | Sakineh Goljarean | Assistant Professor | Persian |
| Person reconciling the 2 forward translations | Prof. Ali Montazeri | Professor | Persian |
| 1^st^ back translator | Zeinab Abdollahi | Master of science | Persian |
| 2^nd^ back translator | Indicated by Prof. Bradley |  |  |

General comments on translation

| *Please insert your name or initials and the date of your comments, e.g.* RHUL (RP) 20-Jan-04 *and put this before your comments for each item.*  RHUL (AW+RP) 6-Oct-11: |
| --- |

RHUL (AW+RP) 13-Dec-10: There are some **‘global’ issues** that occur throughout the questionnaire which we are addressing here, relating to the verbs and their tenses (in the response options), and the intensity of the scales (very much… etc). The issues are linguistically and semantically complex

**1. Response options, location of the verbs and the BTs**

During the FTs we did ask if the format of the statements and response options would keep the verb in the statement and the Response Options separate. e.g اگر دیابت نداشتم، کیفیت زندگی من ..... بود. You mentioned that you would do this if you could -

“*RHUL (AW+RP) 9-Oct-09: We look forward to hearing from you how other Farsi questionnaires handle this problem. If it is possible to do it either way (i.e. both ways), we would like to you to test it both ways in the CD interviews. Given that the forward translators have done it both ways elsewhere in the questionnaire, presumably it must be possible. Please could you check this and let us know.*

*AM-03-Nov-09-This was explained. However, we might also do this if we could*

Unfortunately we didn’t hear any more from you about how other questionnaires have done this and we are therefore assuming that putting the verb into the responses options is the only option*.*

AM-30-May-2011. Although this is correct but we still hope when we do collect data could provide the other form as well in order to see what happens.

RHUL (AW+RP) 6-Oct-11: Thank you. The format we are asking for would need to be presented at CDs to ‘see what happens’ and ideally it should be the first format to be presented so that the interviewee’s reading of it is not ‘contaminated’ by a different process that might actually make this easier. Please see CTGs about CDs and the order in which alternatives are presented, i.e. testing a potentially more difficult version first. You do seem to suggest that it is possible to do it this way, or at least try it, i.e. with the verb remaining in the statement with the dotted line showing where the chosen response should be and we have found it works in other languages/countries where it is necessary. Patients may hesitate a bit the first time they come across it, but then see what it is all about and after that it goes more easily. So what we would therefore like to propose is the following:

1. **Preparation:**
   1. one copy of the questionnaire with it presented the way we think you prefer (with the verbs in the responses) and
   2. another copy with it presented the way we prefer (with the verb in the statement, with the dotted line showing where the chosen response should be).
2. **Test it in the CDs as follows**:
   1. present it our preferred way first, and only if and when people have a problem with it, even after they have tried a few questions,
   2. then present them with the other copy of the questionnaire, with it formatted with the verbs in the response options (your preferred way).

It will be important to do this exercise without the interviewees being aware of any particular preference on the part of the interviewer, so that we can all be confident with the result, whatever it is.

AM-22-Oct-2011. O.K. This will be done according to the instruction.

After checking the translations done by our back translator (“BT2”), we can see that BT2 has interpreted the FT and the structure of the questionnaire exactly as it would be presented in English (i.e. with the verb in the statement), thus providing a ‘fluent’ and professional BT, i.e. it is incorrect to say that it is ‘wrong’; it just isn’t ‘literal’. These English BTs, the sentences and response options etc, have been cut and pasted into the BT report exactly as they were done by BT1 & BT2, thus giving the misleading impression in some items that BT2 has not been done effectively when printed directly below the Farsi FT. It is necessary to look at the whole picture to understand it.

AM- 30-May-2011. Unfortunately we do not understand what you are referring to! Please be clear.

RHUL (AW+RP) 6-Oct-11: Please see explanation below.

From our long experience of this kind of work, we found the repeated statements about BT’s translations (that they were incorrect or careless), to be misleading. We think there has been a misunderstanding of what BT2 has done. There are ‘literal’ translations and ‘fluent’ translations and both have their uses and merits. BT2 has clearly recognised the issue of the structural differences between the two languages (about where the verb needs to be in the Farsi and in the English). S/he also appears to be familiar with questionnaires and has clearly worked out how it would need to be presented in English to give an equivalent representation in an English version. To us it seems, therefore, that s/he has done a professional job, producing a ‘fluent’ and faithful translation, and gives us hope that the Farsi is actually more correct than BT1 suggests (see point #2 below). It is, however, often useful to have a literal translation as well as a fluent one, so that each provides a cross-check on the other translation in relation to the meaning of the FT. So we are perhaps fortunate on this occasion to have a combination of both approaches. But dismissing BT2 is not helpful. So many of BT1’s back-translations give us cause for concern.

AM-30-May-2011. We still believe it is wrong when you back translate form Farsi into English adding something that is not exist in the statement! Of course in general BT2 was more English oriented than BT1. BT1 was more faithful to the statements that were back translated from Farsi into English.

RHUL (AW+RP) 6-Oct -11: By fluent translations we mean that the English reads **fluently**. If the English were presented to the reader as ‘I would very happy be’, this might be a **literal** translation of the original but not fluent/readable English to the reader. The reader would expect, ‘I would be very happy’. BT2 has done a readable (‘fluent’) translation.

You mention that BT1 was more faithful to the statements that were back translated. We think you mean this as more ‘literal’.

After looking at the BT questionnaire created by BT1, we have a problem on another point. We can see that it appears you have the conditional in Overview Item II, but not in any of the subsequent items, where you change to the simple past, i.e. we cannot see any ‘conditional’ form of the verb in the statements; instead, what we see is the past tense e.g. Item 7a “If I did not have diabetes Friendships and my social relationships ….. Improved so much or Became so much better.

AM-22-Oct-2011. We accept your challenge on changing conditional form of the verb to simple past by BT1.

**2. Conditional form of the verbs, “…would be”**

Can you please explain how you deal with the conditional form of a verb?

When looking at all the part (a) response options, there are two possibilities concerning the FT-rec:

**Either**: BT2 is wrong (by using the conditional), BT1 is right (using a past tense), in which case the Farsi FT-rec is quite wrong and needs correcting throughout the questionnaire.

**Or**: BT1 is wrong / misleading, BT2 is correct, and the Farsi is therefore probably OK on this particular point.

**We need to know which – please advise**.

Please see our additional comments for Overview Item II.

AM-30-May-2011. In Farsi conditional sentences could take many several forms depending on to the tense in if clause while in English (to our best knowledge) conditional sentences could take three forms. In English if we understand correctly the structures are:

1. If + simple present, will+ infinitive
2. If + simple past, conditional type I (would + infinitive)
3. If + past perfect, conditional type II (would + have + past participle)

We believe the OE is in the second form. Thus we translated as closest form as possible to the OE. The Farsi form throughout the questionnaire follows this format:

If + simple past, past progressive (past continuous) [past progressive implies that something happened in the past and continuous to the present].

RHUL (AW+RP) 6-Oct-11: There are several conditional forms in English. This conditional form is generally used in hypothetical situations – two examples:

*If I did* ***not*** *have any friends, I would be sad – (But I* ***do*** *have friends)* infinitive so one can’t say *But I did not have friends*.

*If I did* ***not*** *have diabetes, I would have to depend on others (But I* ***do*** *have diabetes)*

The second part of the statement should not contain the past tense, which is what you are showing (and in your case, you are showing it in the response options, whereas we would show it in the statement), i.e. you are saying:

Became so much better, became better, became a little better, did not change, became worse

What you should be saying (if you are putting the verb in the responses options) is *would be so much better,* and not *became...*.

Do you have a conditional which would convey this situation? You mention that Farsi can take several conditional forms, do you have one similar to this? There is no past continuous tense in the statements.

AM-22-Oct-2011. As said before, we agree with you on wrong translation of the conditional form of the verb from OE to Farsi by BT1, but FT-rec is correct.

**3. Strength / intensity of the part (a) response options**

Please read our concept guidelines about this and also our comments for Overview Item II responses. According to the translation of that and the part (a) response options for items 1-19, the FT-rec does not conform to the required degree of intensity needed to make it equivalent to the English and other languages. The result is that people in Iran are likely to score the items differently (probably more highly) and thus show different results from other languages, and data from Iran could not be pooled or compared with data from other language versions. Please see our concept guidelines for the alternatives that have been used in some other languages, where the translators were faced with the same kind of difficulty. As you will see, *much much more (*and: *much much better / easier etc)* can often be used in other languages, or perhaps the equivalent of *very very…* and either of these would make the intensity equivalent to the OE.

AM-30-May-2011. We changed the response categories to:

Very very better, very better, a little better, the same, worse

RHUL (AW+RP) 6-Oct-11: Thank you 🗸

We appreciate that these issues can be slightly challenging in some other languages where the structure of the language is different. However, we have found from past experience that there are ways round these differences and hope you and we will be able to find similar solutions for the Farsi. Please consult our guidelines about these issues. As you will see, we have come across such issues before.

| Item | p.1, Instruction 1 |
| --- | --- |
| **OE** | This questionnaire asks about your quality of life – in other words how good or bad you feel your life to be. |
| **FT-rec** | این پرسشنامه به بررسی کیفیت زندگی شما می پردازد. بعبارت دیگر این پرسشنامه در پی آن است تا احساس خوب یا بد شما را درباره زندگی بررسی نماید. |
| **BT1** | This questionnaire investigates your quality of life. In other words, it intends to find out whether you have a bad or good feeling about life. |
| **BT2** | This questionnaire investigates your quality of life. In other words, this questionnaire seeks to investigate your good or bad feelings about life |
| **Comments** | AM-08-Aug-2010. Instead of **‘asks’** we used **‘investigates’**. In Persian **‘investigate’** could have the same meaning as **‘ask’**. Thus there is no need for any change.  RHUL (AW+RP) 13-Dec-10: In English the two words are not synonymous and we would prefer not to use ***investigate***. It is not just that it has a generally higher reading age and is ‘heavier’, but it is less friendly than the simple expression ***ask about***. It implies you want to “dig” into their lives. It is too strong and could sound more intrusive than *ask about*. Is there some reason why you don’t want to use *ask about*? Just because this is a questionnaire, it isn’t necessary to use very formal wording. Importantly, since this is the very first thing the patients read, it can colour their whole impression of the questionnaire and we don’t want to frighten patients off! The Original English (OE) *ask about* is lighter and less threatening.  There is another slight change in meaning here from the OE: the BT talks about good/bad **feelings** about life. The OE asks if the patient feels their own **life** is good or bad. Is there a way of rephrasing the FT to match the meaning of the OE?  AM-31-May-2011. In most occasions 'ask' in Persian conveys a more materialistic message; that is you are asking for something tangible!  RHUL (AW+RP) 6-Oct-11: What word do you use when asking about someone’s health or for advice? Maybe this verb would be more suitable. |
| **FT rev1** |  |
| **BT rev1** |  |
| **Comments** |  |

| Item | p.1, Instruction 2 |
| --- | --- |
| **OE** | Please put an “X” in the box that best indicates your response for each item |
| **FT-rec** | لطفا پاسخ خود را با علامت × مشخص کنید. |
| **BT1** | Mark your answer using X  Identify your answer by marking X |
| **BT2** | Please indicate your answer with an ‘X’ |
| **Comments** | AM-10-Oct-2010. **‘For each item’** is missing. This was added to FT-rec.  RHUL (AW+RP) 13-Dec-10: Please could you show, in English, how the full statement in FT rev1 is phrased? We have highlighted the bit we think you have added. However, the other thing that is missing appears to be …*that best indicates your response…* Is this somehow included in the existing wording? If not, please add it.  AM-31-May-2011. This was added:  RHUL (AW+RP) 6-Oct-11: Many thanks🗸 |
| **FT rev1** | لطفا پاسخ خود را به هر سوال با علامت × مشخص کنید. |
| **BT rev1** | لطفا بهترین پاسخ مورد نظر خود را به هر سوال با علامت × مشخص کنید. |
| **Comments** |  |

| Item | p.1, Instruction 3 |
| --- | --- |
| **OE** | What we would like to know is how you feel about your life now. |
| **FT-rec** | ما دوست داریم چگونگی احساس شما را درباره زندگی خود بدانیم. |
| **BT1** | We would like to know how you feel about life.  We would like to know the way you will about life |
| **BT2** | We would like to know how you feel about your life. |
| **Comments** | AM-10-Oct-2010. O.K.  RHUL (AW+RP) 13-Dec-10: Although *your* (as in *your life)* is not in either of BT1’s versions, it is in BT2, so we think that should be OK. Could you please confirm it is in the Farsi FT-rec.  The word *now* is in none of the BTs, so we think it must be missing from the Farsi: please would you add it and show the revised version below.  AM-31-May-2011. It is already exists. It was highlighted with green (see above).  RHUL (AW+RP) 6-Oct-11: Thank you. 🗸 |
| **FT rev1** |  |
| **BT rev1** |  |
| **Comments** |  |

| Item | p.1, Overview item (I) |
| --- | --- |
| **OE** | In general, my present quality of life is: |
| **FT-rec** | در مجموع، کیفیت زندگی من در حال حاضر ....... است. |
| **BT1** | Totally, my quality of life is at present ….  In general, my quality of life is presently …..  At present, my quality of life is in general ….. |
| **BT2** | Overall, my quality of life is currently: |
| **Comments** | AM-10-Oct-2010. O.K.  RHUL (AW+RP) 13-Dec-10: 🗸 |
| **FT rev1** |  |
| **BT rev1** |  |
| **Comments** |  |

| Item | p.1, Overview item (I) responses |
| --- | --- |
| **OE** | excellent – very good – good – neither good nor bad – bad – very bad – extremely bad |
| **FT-rec** | عالی، بسیار خوب، خوب، نه خوب نه بد، بد، بسیار بد، بسیار بسیار بد |
| **BT1** | Excellent, very good, good, not good not bad, bad, very bad, terrible  Excellent, very good, good, so-so, bad, very bad, terrible |
| **BT2** | Excellent, very good, good, neither good nor bad, bad, very bad, very very bad |
| **Comments** | AM-10-Oct-2010. O.K.  RHUL (AW+RP) 13-Dec-10:🗸 |
| **FT rev1** |  |
| **BT rev1** |  |
| **Comments** |  |

| Item | p.1, Instruction 4 (to overview Item II) |
| --- | --- |
| **OE** | Now we would like to know how your quality of life is affected by your diabetes, its management ****(including medication, visits to the doctor, and food)* and any complications you may have. |
| **FT-rec** | اکنون ما تمایل داریم بدانیم دیابت، درمان آن (شامل معاینات دوره ای، دستورهای داروئی و رژیم غذائی) و عوارض این بیماری چگونه بر کیفیت زندگی شما تاثیر گذاشته است. |
| **BT1** | Now, we would like to know how diabetes, its therapy (including periodical examination, medical prescriptions, and nutritive regimens) *and complications influence your quality of life*. |
| **BT2** | Now we would like to know how diabetes, its treatment (including periodic examinations, prescription medicines, and diet), and its symptoms have affected your quality of life |
| **Comments** | AM-10-Oct-2010. It seems that the best meaning for management in this context is treatment.  RHUL (AW+RP) 13-Dec-10: The statement has been turned around with ‘quality of life’ at the end of the sentence. Is there a reason for this? Is this perhaps necessary for the sentence structure in Farsi? Or is this so that you can use an ‘active’ rather than ‘passive’ verb? We think it should be OK, but please clarify and confirm.  AM-31-May-2011. This is due to the structure of a sentence in Persian as described before. At present our translation is active. We could also change it to passive. It is now a very readable Persian sentence!  RHUL (AW+RP) 6-Oct-11: OK, thank you – no change then 🗸  BT1 mentions ‘complications’ which is in the OE but BT2 mentions ‘symptoms’ which is slightly different. BT2 is closer to the OE apart from this one word. It is important that any actual complications are considered and not just the symptoms of such complications. Which is closer to the Farsi on this point, BT1 or BT2?  AM-28 May-2011. BT1.  RHUL (AW+RP) 6-Oct-11: OK, thank you – no change. 🗸 |
| **FT rev1** |  |
| **BT rev1** |  |
| **Comments** |  |

*** Please ensure that you have translated the section in brackets in case you encounter any problems later during the CDs. You may need to include this in your CDs (perhaps as an Alternative), in case you find the word you have had to use in your language for *management* doesn’t have a sufficiently broad meaning compared with the OE.

| Item | p.1, Overview Item (II) |
| --- | --- |
| **OE** | If I did not have diabetes, my quality of life would be: |
| **FT-rec** | اگر دیابت نداشتم، کیفیت زندگی من .......... . |
| **BT1** | If I did not contract diabetes, my quality of life would be  If I did not have diabetes, my quality of life would be |
| **BT2** | If I did not have diabetes, my quality of life would be…. |
| **Comments** | AM-10-Oct-2010. **‘Would be’** does not exist in FT-rec while BT2 used **‘would be’**! In general FT-rec is acceptable.  RHUL (AW+RP) 13-Dec-10: Please see our ‘global’ comment #2 on Page 2.  We are confused by your statements. You say *would be* is not in the FT-rec. However, not just BT2 but both of BT1’s versions show it is. Do they mean that it is included in the statement of this item, but in practice the verb is in the responses? It seems nevertheless that both translators have seen something in the Farsi FT-rec that suggests the ‘conditional’ meaning? If that is so, could you consider how you have done the translation of the conditional here and see if you can use the same structure in other places, where we should have the conditional, but where BT1 implies that you have the past tense instead)?  AM-31-May-2011. Please see response to global comment #2.  RHUL (AW+RP) 6-Oct-11: Sorry, we can’t see a clear response there which answers our question.  What does the Farsi actually say, if **both** translators (including yours) say “If I did not have diabetes, my quality of life would be” and you say **‘Would be’** does not exist in FT-rec. We are just rather confused here. Please see our global comments for today’s date at the top of the report. |
| **FT rev1** |  |
| **BT rev1** |  |
| **Comments** |  |

| Item | p.1, Overview Item (II) responses |
| --- | --- |
| **OE** | very much better – much better – a little better – the same – worse |
| **FT-rec** | خیلی بهتر می شد ، بهتر می شد، کمی بهتر می شد، فرقی نمی کرد، بدتر می شد |
| **BT1** | Improved so much, improved, almost improved, did not differ, worsened  Became so much better, became better, became a little better, did not change, became worse  Became so much better, became better, became a little better, was not different, became worse |
| **BT2** | Much better, Better, Somewhat better, The same as before, Worse |
| **Comments** | AM-10-Oct-2010. Both translations by BT1 and BT2 are wrong and do not convey FT-rec. However, FT-rec is best to convey OE.  RHUL (AW+RP) 13-Dec-10: There are a few problems with the current scale in Farsi:   1. Please see ‘global’ issue #3 at the top. The OE has ***very*** *much better, much better, a little better*). We need to have something stronger for the two strongest. In some languages where it is not possible to say *much better* (or similarly for other items, *much more…*), the solution has been to use something like *much much better* or *very very better.* Even though the latter structure would not be use in English, the first could be, and one or other of these can be (and has been) used in some other languages. The problem is that if you leave this as it is, it is a weaker scale at the top end and is therefore likely to cause people to score this differently from other languages and it is likely that people would score more to the high end. This could also ….. Please advise.   AM-31-May-2011. We changed the two strongest response categories to very very better, very better  خیلی خیلی بهتر می شد، خیلی بهتر می شد  RHUL (AW+RP) 6-Oct-11: Thank you, that’s good news. 🗸  2. Please see global issue #2 at the top. We understand your need, for structural reasons, to have the verb in the scale. However, tense/voice of the verb needs to be absolutely clearly *would be*. The problem shows up particularly with the strongest *(improved so much…*or…*became so much better)* and the least strong *(almost improved…*or…*became a little better*), and also with the translation of the same *(did not differ / did not change* and (BT2) *the same as before*). BT2’s translation confirms the main feature of all the BT1 versions, namely that the verb seems to be in the past tense. Is there some reason why you can clearly have *would be* in Overview Item II (confirmed by all BTs) but not here in the scales? Unfortunately, it is confusing if the general interpretation is that this is in the past tense. One particular problem applies to the translation of *the same*. This should not be ‘in comparison with before’, but both BTs have translated it this way, suggesting that the problem lies in the Farsi and not in either of the BTs. The past tense and the comparison drawn as a result of its use make no sense in the context of this questionnaire.  As an example, some people may have had diabetes since early childhood and not be able to remember how they would have perceived their quality of life (QoL) before they had diabetes. It is not a comparison of ‘now’ with ‘before’ but a comparison of ‘life as it is with diabetes’ and an imagined situation of ‘how life would be [now] if I did not have diabetes’. When interviewing patients during the development of this and QoL questionnaires for other chronic diseases, we ask people to imagine that, by some miracle, their diabetes ‘goes away’ and they don’t have diabetes any more, and we ask them then to consider how different their QoL would be without diabetes.  We do appreciate that your language is structured differently from English, but we have come across similar differences with other languages and an equivalent to the English ‘conditional’ voice/tense of the verb has been possible in some form. We look forward to hearing from you how both these two issues can be made clearer and closer to the OE.  AM-31-May-2011. Please see response to global comment #2.  RHUL (AW+RP) 6-Oct-11: Please see our further explanation as to the conditional used. You cannot say (or certainly not in English), “If I did not have diabetes, my quality of life became so much better”. Please review our global comments on this. |
| **FT rev1** | خیلی خیلی بهتر می شد، خیلی بهتر می شد، کمی بهتر می شد، فرقی نمی کرد، بدتر می شد |
| **BT rev1** |  |
| **Comments** |  |

| Item | p.2, Instruction 1, sentence 1 |
| --- | --- |
| **OE** | Please respond to the more specific statements on the following pages. |
| **FT-rec** | لطفا به سوالات مخصوصی که در زیر آمده است، پاسخ دهید. |
| **BT1** | Please answer the following specified questions |
| **BT2** | Please answer the specific questions below. |
| **Comments** | AM-10-Oct-2010. O.K. (although **‘more’** is missing from more specific)  RHUL (AW+RP) 13-Dec-10: Are you saying ‘more’ is not in the FT-rec or just missing from the BT?  AM-31-May-2011. I meant from FT-rec. We revised it to convey that it is more specific. We did this by using another word from similar route in Farsi.  RHUL (AW+RP) 6-Oct-11:Many thanks🗸 |
| **FT rev1** | لطفا به سوالات اختصاصی که در زیر آمده است، پاسخ دهید. |
| **BT rev1** |  |
| **Comments** |  |

| Item | p.2, Instruction 1, sentence 2 |
| --- | --- |
| **OE** | For each aspect of life described, you will find two parts: |
| **FT-rec** | برای توصیف هر جنبه از زندگی دو قسمت مشاهده خواهید کرد. |
| **BT1** | You will observe two parts for the description of each life aspect. |
| **BT2** | You will find two sections covering each aspect of life: |
| **Comments** | AM-10-Oct-2010. O.K.  RHUL (AW+RP) 13-Dec-10:🗸 |
| **FT rev1** |  |
| **BT rev1** |  |
| **Comments** |  |

| Item | p.2 instruction box, part (a) instruction |
| --- | --- |
| **OE** | For Part (a): put an “X” in one box to show how diabetes affects this aspect of your life; |
| **FT-rec** | بخش الف- با زدن علامت × در یکی از مربع ها نشان دهید دیابت چگونه بر این جنبه از زندگی شما تاثیر گذاشته است |
| **BT1** | Part (a). Use X to show how diabetes affects this aspect of your life. |
| **BT2** | Part A- By placing an ‘X’ in one of the boxes, indicate how diabetes has affected this aspect of your life |
| **Comments** | AM-10-Oct-2010. O.K.  RHUL (AW+RP) 13-Dec-10:🗸 |
| **FT rev1** |  |
| **BT rev1** |  |
| **Comments** |  |

| Item | p.2 instruction box, part (b) instruction |
| --- | --- |
| **OE** | For Part (b): put an “X” in one box to show how important this aspect of your life is to your quality of life. |
| **FT-rec** | بخش ب- با زدن علامت × در یکی از مربع ها اهمیت هر جنبه از زندگی خود را نشان دهید |
| **BT1** | Part (b). use X to show the importance of each aspect of your life |
| **BT2** | Part B- By placing an ‘X’ in one of the boxes, indicate the importance of this aspect of your life |
| **Comments** | AM-10-Oct-2010. O.K.  RHUL (AW+RP) 13-Dec-10: That seems fine for part of the sentence, but you seem (according to both BTs) to have left out *to your quality of life*. If you have difficulty having both *this aspect of your life…* and …*to your quality of life,* you could shorten it and have just …*how important this aspect is to your quality of life*. That would be fine. The important thing is to link it to *quality of life.*  AM-31-May-2011. This was revised as suggested.  RHUL (AW+RP) 6-Oct-11: Thank you, could you please give us the English translation of the revision – a literal one – and insert it below. |
| **FT rev1** | بخش ب- با زدن علامت × در یکی از مربع ها اهمیت هر جنبه را بر روی کیفیت زندگی خود نشان دهید |
| **BT rev1** |  |
| **Comments** |  |

| Item | (1a) statement |
| --- | --- |
| **OE** | …I would enjoy my leisure activities |
| **FT-rec** | از فعالیت های تفریحی ......... . |
| **BT1** | From fun activities …..  From entertainments …. |
| **BT2** | I would enjoy leisure activities…. |
| **Comments** | AM-10-Oct-2010. BT2 is wrong back translation since it includes the verb. The statement does not contain the verb **‘would’**. As agreed **‘would’** is included in response categories.  RHUL (AW+RP) 13-Dec-10: We understand that; however, please see our ‘global’ comments #1 (concerning the location of the verb) & #2 concerning the conditional.  AM-31-May-2011-Please see responses to global comment #1 and #2.  RHUL (AW+RP) 6-Oct-11: I’m sorry, we cannot see ‘would’ included in BT1 response options.  BT2’s use of *leisure activities’* is closer to the OE. *Leisure activities* can be quiet and relaxing as well as active. BT1’s translations seem rather active and do not seem to consider quieter pastimes, e.g. reading. We chose ‘leisure activities’ in order to include both quiet and active things. Can you please confirm that BT2’s translation is the closest of the three BTs to the Farsi? It will also be interesting to see what examples this item makes the patients think about in the CDs. Please would you make a note to ask the patients to give examples of what it makes them think of.  AM-31-May-2011. In fact considering your explanations none of the back ward translations are correct. We changed the word (تفریحی) to (سرگرم کننده) to convey the OE and BT2  RHUL (AW+RP) 6-Oct-11: Thank you. Can you give us a (literal) translation, in particular, is “my” included? |
| **FT rev1** | از فعالیت های سرگرم کننده ..................... |
| **BT rev1** |  |
| **Comments** |  |

| Item | (1a) responses |
| --- | --- |
| **OE** | very much more – much more – a little more – the same – less |
| **FT-rec** | خیلی بیشتر لذت می بردم ، ییشتر لذت می بردم ، کمی بیشتر لذت می بردم ، فرقی نمی کرد، کمتر لذت می بردم |
| **BT1** | Enjoyed much more, enjoyed more, enjoyed a little more, did not differ, enjoyed less |
| **BT2** | Much more, More, Somewhat more, The same as before, Less |
| **Comments** | AM-10-Oct-2010. Both BT1 and BT2 are careless translations. However, FT-rec is a good reflection of the OE bearing to mind that as agreed the response categories are include the verb **‘would’**.  RHUL (AW+RP) 13-Dec-10: Please see our comment #2 on P2. Neither BTs have ‘**very**’. Is it possible to have ‘**very** much more’ or ‘much much more’ (or an equivalent to ‘very very more’)? Please see our global #2 comments and those for the Overview Item II responses. The scale needs increasing in intensity. BT1 has the wrong tense, ‘enjoyed’. Is this why you think BT2’s translation is careless? BT1 suggests the FT-rec is in the past tense instead of the conditional. Please advise.  AM-31-May-2011. Please see our responses to global comment #2. BT2 did not include verb and included (as before) to (The same) and BT1 could not understand past progressive and back translated it to simple past while the verb is in past progressive as explained before.  RHUL (AW+RP) 6-Oct-11: The verb should not be in the past progressive. Please see the construction of the statements in our comments above #2  However, we changed response categories to very very more and very more as you can see below.  RHUL (AW+RP) 6-Oct-11: Sorry, we don’t understand (and can’t read) Persian, so we can’t “see” this below but we accept your confirmation that this has been done – thank you. 🗸 |
| **FT rev1** | خیلی خیلی بیشتر لذت می بردم ، خیلی بیشتر لذت می بردم ، کمی بیشتر لذت می بردم ، فرقی نمی کرد، کمتر لذت می بردم |
| **BT rev1** |  |
| **Comments** |  |

| Item | (1b) statement |
| --- | --- |
| **OE** | My leisure activities are |
| **FT-rec** | فعالیت های تفریحی برای من ....... . |
| **BT1** | For me, fun activities ….  For me, entertaining activities ….. |
| **BT2** | For me, leisure activities are: |
| **Comments** | AM-10-Oct-2010. O.K.  RHUL (AW+RP) 13-Dec-10: Please see our comments for 1a.  AM-31-May-2011. Please see our response for 1a statement.  RHUL (AW+RP) 6-Oct-11: Thank you🗸 |
| **FT rev1** | فعالیت های سرگرم کننده برای من ................ |
| **BT rev1** |  |
| **Comments** |  |

| Item | (1b) responses |
| --- | --- |
| **OE** | very important – important – somewhat important – not at all important |
| **FT-rec** | خیلی مهم هستند، مهم هستند، تا حدی مهم هستند، اصلا مهم نیستند |
| **BT1** | Are very important, are important, are almost important, are not important at all.  Are very important, are important, are almost important, are unimportant. |
| **BT2** | Very important, Important, Somewhat important, Not important at all |
| **Comments** | AM-10-Oct-2010. BT2 is careless translation. However, BT1 is O.K. FT-rec is a good reflection of the OE bearing to mind that as agreed the response categories are include the verb **‘are’**.  RHUL (AW+RP) 13-Dec-10: Please see our ‘global’ comment #1 on P2. BT2’s translation is far from careless; it is just that it is fluent rather than ‘literal’.  AM-31-May-2011. Still believe BT2 is careless since translator did not included verb in his or her translation. Back translation should be an honest reflection of what one should translate from target language to the original. This is not fluent rather than literal! This is simply wrong translation or perhaps BT2 was familiar with original questionnaire and response categories beforehand!!  RHUL (AW+RP) 6-Oct-11: BT2 has put the sentence into readable English to create an English version of the Farsi FT rather than producing a literal translation. We seem to differ in our views of what is an honest reflection (and have just been reading about exactly this issue in the Chartered Institute of Linguists’ latest journal where they discuss these different types of translation and the need for both kinds). The error if any is perhaps on our part that we did not specifically ask the local back translator to give us a totally literal translation rather than a readable/fluent one. We usually leave it to their judgement as to which is more useful for our particular purposes; clearly their judgement differed from yours on this occasion. Our understanding was that s/he was trying to make clear to us, without having to annotate the text, that this was the English equivalent of the Farsi construction, as the Farsi construction is not one that would be used in an English questionnaire. And no, they certainly had no sight of the questionnaire; that is Rule #1 for back translations. However, the structure of the questionnaire is a commonly used kind of format, and the translator’s approach suggested they have previous experience in translating questionnaires like ours from Farsi.  It is more reassuring from our point of view than BT1’s translation for *somewhat important*. In English we would not say ‘almost important’. Your comment that BT1’s translation is a good reflection of the OE concerns us, because it reflects a misunderstanding of the difference between *almost* and *somewhat.* The reason for our concern is that, if you say something is *almost important*, it implies that in fact it is not important, i.e. it lacks importance (even if the implication is that it is nearly important, it still is not). Please read the guidelines about the part (b) scale. A response that implies this is not actually important would put the response on the wrong side of the (invisible) neutral point and would change the way people scored items on this scale, making it different from other languages. We are somewhat reassured by BT2’s translation as it reflects the original English exactly. However, we need to know which of these is a more precise reflection of the Farsi. If BT1 is, then we need to change it to something closer to the OE. Please advise.  AM-31-May-2011. Our concern was about not including verb ‘are’ in BT2’s translation and not about the word Somewhat. However, as you indicated here the BT1’s translation of Somewhat was wrong although the FT-rec is exactly reflecting the word somewhat.  RHUL (AW+RP) 6-Oct-11: Thank you for confirming that the FT-rec shows ‘somewhat’ and not ‘almost’. No change on this point 🗸 |
| **FT rev1** |  |
| **BT rev1** |  |
| **Comments** |  |

| Item | (2) preliminary question |
| --- | --- |
| **OE** | Are you currently working, looking for work or would you like to work? |
| **FT-rec** | آیا در حال حاضر مشغول به کار هستید؟ یا در جستجوی کار هستید؟ یا علاقمند به کار کردن هستید؟ |
| **BT1** | Are you working at present, Are you looking for a job, Or are you interested in working? |
| **BT2** | Are you currently working? Are you looking or interested in working? |
| **Comments** | AM-10-Oct-2010. O.K.  RHUL (AW+RP) 13-Dec-10:🗸 Fine. |
| **FT rev1** |  |
| **BT rev1** |  |
| **Comments** |  |

| Item | (2) Yes/No answer options |
| --- | --- |
| **OE** | Yes …… If *yes*, complete (a) and (b)  No …… If *no*, go straight to 3a |
| **FT-rec** | اگر پاسخ شما مثبت است گزینه های الف و ب را تکمیل نمائید. در غیر اینصورت مستقیما به قسمت الف سوال 3 بروید. |
| **BT1** | If yes, complete parts a and b, if no, go straight to question 3, part a. |
| **BT2** | If your answer is yes, complete sections A and B. Otherwise, go directly to question 3A. |
| **Comments** | AM-10-Oct-2010. O.K.  RHUL (AW+RP) 13-Dec-10: 🗸 |
| **FT rev1** |  |
| **BT rev1** |  |
| **Comments** |  |

| Item | (2a) statement |
| --- | --- |
| **OE** | …my working life would be: |
| **FT-rec** | وضعیت کاری من ........ . |
| **BT1** | My job status …  My job condition …. |
| **BT2** | my work situation would be… |
| **Comments** | AM-10-Oct-2010. BT1 is O.K. BT2 is wrong back translation since it includes the verb.  RHUL (AW+RP) 13-Dec-10:🗸 Please see our comment on P2 – BT2 is conceptually accurate.  AM-31-May-2011. This was explained before.  RHUL (AW+RP) 6-Oct-11: 🗸 |
| **FT rev1** |  |
| **BT rev1** |  |
| **Comments** |  |

| Item | (2a) responses |
| --- | --- |
| **OE** | very much better – much better – a little better – the same – worse |
| **FT-rec** | خیلی بهتر می شد، بهتر می شد، کمی بهتر می شد، فرقی نمی کرد، بدتر می شد |
| **BT1** | Improved so much, improved, almost improved, did not differ, worsened  Became so much better, became better, became a little better, did not change, became worse  Became so much better, became better, became a little better, was not different, became worse |
| **BT2** | Much better, Better, Somewhat better, The same as before, Worse |
| **Comments** | AM-10-Oct-2010. Both BT1 and BT2 are careless translations. However, FT-rec is a good reflection of the OE bearing to mind that as agreed the response categories are include the verb **‘would’**.  RHUL (AW+RP) 13-Dec-10: Please see our comment on P2. The tense in BT1 is wrong.  Again, the matter of the intensity of the response options. We need to find some way to get the equivalent of ‘**very** much better’?  AM-31-May-2011. The response categories were revised.  RHUL (AW+RP) 6-Oct-11: Thank you for changing the intensity (as mentioned earlier in the report).  ‘Would’ does not seem to appear in the BT1 response categories. Please could you highlight where it is. The verb tense shown here (improved, became) is not the same as OE. Are we to understand that you have included a verb (in whatever form) in the response options here also? |
| **FT rev1** | خیلی خیلی بهتر می شد، خیلی بهتر می شد، کمی بهتر می شد، فرقی نمی کرد، بدتر می شد |
| **BT rev1** |  |
| **Comments** |  |

| Item | (2b) statement |
| --- | --- |
| **OE** | For me, having a working life is: |
| **FT-rec** | برای من داشتن کار ......... . |
| **BT1** | For me, having a job ….. |
| **BT2** | For me, working is: |
| **Comments** | AM-10-Oct-2010. O.K.  RHUL (AW+RP) 13-Dec-10:🗸 |
| **FT rev1** |  |
| **BT rev1** |  |
| **Comments** |  |

| Item | (2b) responses |
| --- | --- |
| **OE** | very important – important – somewhat important – not at all important |
| **FT-rec** | خیلی مهم است، مهم است، تا حدی مهم است، اصلا مهم نیست |
| **BT1** | Is very important, is important, is almost important, is not important at all.  is very important, is important, is almost important, is unimportant. |
| **BT2** | Very important, Important, Somewhat important, Not important at all |
| **Comments** | AM-10-Oct-2010. BT2 is a careless translation. However, FT-rec is a good reflection of the OE bearing to mind that as agreed the response categories include the verb **‘is’**.  RHUL (AW+RP) 13-Dec-10: Please global issue #1; also our comment for item 1b responses.  AM-31-May-2011. Please see our previous explanations.  RHUL (AW+RP) 6-Oct-11: Thank you for confirming that the FT-rec shows ‘somewhat’ and not ‘almost’.🗸 |
| **FT rev1** |  |
| **BT rev1** |  |
| **Comments** |  |

| Item | (3a) statement |
| --- | --- |
| **OE** | … local or long distance journeys would be |
| **FT-rec** | رفتن به جاهای دور و نزدیک ..... . |
| **BT1** | Going to near and far places ….. |
| **BT2** | going to places near and far would be…. |
| **Comments** | AM-10-Oct-2010. Both BT1 and BT2 used **‘going’** for **‘journey’**. In addition BT2 included **‘would’** while it does not exist in FT-rec. I am not sure about using the word **‘going’** instead of **‘journey’**. However, FT-rec is a good reflection of the OE bearing to mind that as agreed the response categories include the verb **‘would’**.  RHUL (AW+RP) 13-Dec-10: The meaning is understood here, however, as going to a far place would entail a long journey, so we think this will be OK, but it needs to be checked carefully in the CD interviews. 🗸  AM-31-May-2011-. O.K.  RHUL (AW+RP) 6-Oct-11: 🗸 thanks. |
| **FT rev1** |  |
| **BT rev1** |  |
| **Comments** |  |

| Item | (3a) responses |
| --- | --- |
| **OE** | very much easier – much easier – a little easier – the same – more difficult |
| **FT-rec** | خیلی آسانتر می شد، آسانتر می شد، کمی آسانتر می شد، فرقی نمی کرد، سخت تر می شد |
| **BT1** | Became so easier, became easier, became almost easier, did not differ, became worse |
| **BT2** | Much easier, Easier, Somewhat easier, More difficult |
| **Comments** | AM-10-Oct-2010. BT2 is a careless translation. However, FT-rec is a good reflection of the OE bearing to mind that as agreed the response categories include the verb **‘would be’**.  RHUL (AW+RP) 13-Dec-10: Please see global issue #3 at the top – we need to get this closer to the OE. Also global issue #2: the verb appears to be the wrong tense/form of the verb according to BT1.  AM-31-May-2011. Please see our responses.  RHUL (AW+RP) 6-Oct-11: We assume from the FT-rev that the responses have been revised. Please could you show the English. Many thanks |
| **FT rev1** | خیلی خیلی آسانتر می شد، خیلی آسانتر می شد، کمی آسانتر می شد، فرقی نمی کرد، سخت تر می شد |
| **BT rev1** |  |
| **Comments** |  |

| Item | (3b) statement |
| --- | --- |
| **OE** | For me, local or long distance journeys are |
| **FT-rec** | داشتن توانائی برای رفتن به جاهای دور و نزدیک ......... . |
| **BT1** | Being able to go to near and far places ….. |
| **BT2** | Having the ability to go to places near and far is…. |
| **Comments** | AM-10-Oct-2010. **‘Being able’** or **‘having the ability’** does not exist in OE but it seems that the concept of FT-rec is very similar to OE except that it does not contain the verb **‘are’**.  RHUL (AW+RP) 13-Dec-10:🗸 We think this should be OK. |
| **FT rev1** |  |
| **BT rev1** |  |
| **Comments** |  |

| Item | (3b) responses |
| --- | --- |
| **OE** | very important – important – somewhat important – not at all important |
| **FT-rec** | خیلی مهم است، مهم است، تا حدی مهم است، اصلا مهم نیست |
| **BT1** | Are very important, are important, are almost important, are not important at all.  Are very important, are important, are almost important, are unimportant. |
| **BT2** | Very important, Important, Somewhat important, Not important at all |
| **Comments** | AM-10-Oct-2010. BT2 is a careless translation. In BT1, 'are' should be 'is'. However, FT-rec is a good reflection of the OE bearing to mind that as agreed the response categories include the verb **‘are’**.  RHUL (AW+RP) 13-Dec-10: Please see our comment for item 1b responses concerning *almost*.  AM-31-May-2011. Please see our response.  RHUL (AW+RP) 6-Oct-11:🗸 |
| **FT rev1** |  |
| **BT rev1** |  |
| **Comments** |  |

| Item | (4) preliminary question |
| --- | --- |
| **OE** | Do you ever go on holiday or want to go on holiday? |
| **FT-rec** | آیا اصلا در تعطیلات به مسافرت می روید یا دوست دارید که به مسافرت بروید؟ |
| **BT1** | Do you ever go on a vacation in holidays or do you like to do so? |
| **BT2** | Do you travel during the holidays or would you like to travel? |
| **Comments** | AM-16-Oct-2010. Both BT1 and BT2 back translated the FT-rec word by word. However, the concept of FT-rec and OE are the same.  RHUL (AW+RP) 13-Dec-10:🗸 This question is primarily about the ‘vacation’ and not the travelling. But we think this is probably fine. Could you just tell us which back translation is more precise: BT1 with ‘go’ or BT2 with ‘travel’? We are aware that in the concept guidelines we have said that this is about the kind of holidays where you go away from home, so if ‘travel’ is essential to making this clear (as it appears from your comment in part (a) below), we can accept that.  AM-31-May-2011. It seems that BT2 is more precise.  RHUL (AW+RP) 6-Oct-11:🗸 |
| **FT rev1** |  |
| **BT rev1** |  |
| **Comments** |  |

| Item | (4a) statement |
| --- | --- |
| **OE** | … my holidays would be |
| **FT-rec** | مسافرتم ........ . |
| **BT1** | My journey ….  My travel ….  My vacation …. |
| **BT2** | my travels would be… |
| **Comments** | AM-16-Oct-2010. Again the problem occurred. The word **‘holiday’** in OE means **‘going to a vacation’** or **‘travelling somewhere’** but in Persian **‘holiday’** necessarily does not mean **‘going to a vacation’**. Thus in Persian we should use both words (holiday and travelling or similar words). BT2 is wrong back translation since it includes the verb.  RHUL (AW+RP) 13-Dec-10: It seems you are saying that you do not have an expression ‘to go on holiday’ meaning to go away on holiday. So we understand the need to say ‘travel on holiday’ (or the American, ‘vacation’).  AM-31-May-2011. Exactly.  RHUL (AW+RP) 6-Oct-11:🗸 thanks. |
| **FT rev1** |  |
| **BT rev1** |  |
| **Comments** |  |

| Item | (4a) responses |
| --- | --- |
| **OE** | very much better – much better – a little better – the same – worse |
| **FT-rec** | خیلی بهتر می شد، بهتر می شد، کمی بهتر می شد، فرقی نمی کرد، بدتر می شد |
| **BT1** | Improved so much, improved, almost improved, did not differ, worsened  Became so much better, became better, became a little better, did not change, became worse  Became so much better, became better, became a little better, was not different, became worse |
| **BT2** | Much better, Better, Somewhat better, The same as before, Worse |
| **Comments** | AM-16-Oct-2010. Both translations by BT1 and BT2 are wrong and do not convey FT-rec. However, FT-rec is best to convey OE.  RHUL (AW+RP) 13-Dec-10: It is good to know that BT1 is not correct: both ‘almost’ and the past tense (‘became’, ‘did not’ etc) are cause for concern. Please see global issue #2: also global issue #3 about ‘very much’.  AM-31-May-2011. Please see our previous explanations on this.  RHUL (AW+RP) 6-Oct-11: Please could you give the English version of your revisions. Many thanks |
| **FT rev1** | خیلی خیلی بهتر می شد، خیلی بهتر می شد، کمی بهتر می شد، فرقی نمی کرد، بدتر می شد |
| **BT rev1** |  |
| **Comments** |  |

| Item | (4b) statement |
| --- | --- |
| **OE** | For me, holidays are |
| **FT-rec** | برای من رفتن به مسافرت در تعطیلات ........ . |
| **BT1** | For me, going on a vocation in holidays …. |
| **BT2** | For me, travelling during the holidays is… |
| **Comments** | AM-16-Oct-2010. O.K. As agreed **‘are’** not included in FT-rec.  RHUL (AW+RP) 13-Dec-10: See our comments for 4a.  AM-31-May-2011. Please see our previous response to this.  RHUL (AW+RP) 6-Oct-11: The concept is conveyed here. 🗸 Please see the discussions regarding the position of the verb and tense. |
| **FT rev1** |  |
| **BT rev1** |  |
| **Comments** |  |

| Item | (4b) responses |
| --- | --- |
| **OE** | very important – important – somewhat important – not at all important |
| **FT-rec** | خیلی مهم است، مهم است، تا حدی مهم است، اصلا مهم نیست |
| **BT1** | Are very important, are important, are almost important, are not important at all.  Are very important, are important, are almost important, are unimportant. |
| **BT2** | Very important, Important, Somewhat important, Not important at all |
| **Comments** | AM-16-Oct-2010. BT2 did not include ‘**is’** in response categories. BT1 also uses **‘are’** instead of **‘is’**. However, FT-rec is O.K.  RHUL (AW+RP) 13-Dec-10: Please see our comment for item 1b responses concerning *almost*, and global issue # 2 about the verb tense.  AM-31-May-2011. Please see our previous response to this.  RHUL (AW+RP) 6-Oct-11:Discussed at beginning of report. |
| **FT rev1** |  |
| **BT rev1** |  |
| **Comments** |  |

| Item | (5a) statement |
| --- | --- |
| **OE** | … physically I could do |
| **FT-rec** | می توانستم به لحاظ جسمی فعالیت های ........ . |
| **BT1** | I could …… activities physically. |
| **BT2** | my physical activities would… |
| **Comments** | AM-16-Oct-2010. BT2 is wrong back translation since it includes the verb. BT1 is relatively a better back translation.  RHUL (AW+RP) 13-Dec-10: The difficulty arises here (because you need to put the verb in the responses) that  AM-31-May-2011. Exactly.  RHUL (AW+RP) 6-Oct-11:BT1 does have the verb in the statement here – we await your responses to our comments of today’s date on this issue in the ‘global’ comments at the top of the report. |
| **FT rev1** |  |
| **BT rev1** |  |
| **Comments** |  |

| Item | (5a) responses |
| --- | --- |
| **OE** | very much more – much more – a little more – the same – less |
| **FT-rec** | خیلی بیشتری انجام دهم، بیشتری انجام دهم، کمی بیشتری انجام دهم، فرقی نمی کرد، کمتری انجام دهم |
| **BT1** | Do much more, do more, do almost more, did not differ, do less |
| **BT2** | Increase significantly, Increase, Increase somewhat, Stay the same as before, Decrease |
| **Comments** | AM-16-Oct-2010. Considering the statement (5a), FT-rec is O.k. However, both BT1 and BT2 are wrong back translation.  RHUL (AW+RP) 13-Dec-10: Could you please tell us what FT-rec does say and suggest why BT1 and BT2 are wrong? There appears to be the same problem as elsewhere about the tense of the verb. However, the verb itself is problematic. Both BT1 (‘do…’) and BT2 (‘increase’) suggest that activities would simply increase. However the OE says ‘I could do’, i.e. ‘I would be able to do’. We need to include the modal verb ‘can / be able to’ here, but again, in the conditional form. Please review and advise.  AM-31-May-2011. I would be able included in the statement (the first word) and to do was included in the response categories (the last word) to present it as closest possible as to OE.  RHUL (AW+RP) 6-Oct-11: Please could you show the English version of your revision and also the English translation of the full statement with response options. This does not appear clear. |
| **FT rev1** | خیلی خیلی بیشتری انجام دهم، خیلی بیشتری انجام دهم، کمی بیشتری انجام دهم، فرقی نمی کرد، کمتری انجام دهم |
| **BT rev1** |  |
| **Comments** |  |

| Item | (5b) statement |
| --- | --- |
| **OE** | For me, how much I can do physically is |
| **FT-rec** | برای من مقدار کاری که به لحاظ جسمی انجام می دهم، ........ . |
| **BT1** | For me, the amount of physical work I do ….  For me, the amount of work I do physically …. |
| **BT2** | For me, the amount of physical activity I carry out is …. |
| **Comments** | AM-16-Oct-2010. Since **‘can’** was missing in FT-rec, this was revised.  RHUL (AW+RP) 13-Dec-10: Thank you for adding ‘can’. As mentioned above, it needs to be added to part (a) as well and no equivalent change seems to have been made there.  We don’t want to make patients think only of physical ‘work’, but also of basic things like walking, and going up and downstairs. Please could you say what the FT-rec says now? If BT2 is accurate, this may be OK as that suggests the FT-rec is general enough to cover all those things. If BT1 is the more precise back translation, then the FT-rec needs to be revised. Please review and advise.  AM-31-May-2011. FT-rec now is saying For me the amount of work that I can do physically  RHUL (AW+RP) 6-Oct-11: Thank you🗸 |
| **FT rev1** | برای من مقدار کاری که می توانم به لحاظ جسمی انجام دهم، ....... . |
| **BT rev1** |  |
| **Comments** |  |

| Item | (5b) responses |
| --- | --- |
| **OE** | very important – important – somewhat important – not at all important |
| **FT-rec** | خیلی مهم است، مهم است، تا حدی مهم است، اصلا مهم نیست |
| **BT1** | Are very important, are important, are almost important, are not important at all.  Are very important, are important, are almost important, are unimportant. |
| **BT2** | Very important, Important, Somewhat important, Not important at all |
| **Comments** | AM-16-Oct-2010. BT2 is wrong. In BT1 **‘are’** should be **‘is’**. However, FT-rec is acceptable.  RHUL (AW+RP) 13-Dec-10: Please see our comment for item 1b responses concerning *almost*, and global issue # 2 about the verb tense.  AM-31-May-2011. Please our previous explanations.  RHUL (AW+RP) 6-Oct-11: This has been explained🗸 |
| **FT rev1** |  |
| **BT rev1** |  |
| **Comments** |  |

| Item | (6) preliminary question |
| --- | --- |
| **OE** | Do you have any family / relatives? |
| **FT-rec** | آیا خانواده یا خویشاوندی دارید؟ |
| **BT1** | Do you have a family or relatives? |
| **BT2** | Do you have family or relatives? |
| **Comments** | AM-16-Oct-2010. **‘Any’** is missing in BT2 and it was changed to **‘a’** in BT1. However, FT-rec is acceptable.  RHUL (AW+RP) 13-Dec-10: 🗸 |
| **FT rev1** |  |
| **BT rev1** |  |
| **Comments** |  |

| Item | (6a) statement |
| --- | --- |
| **OE** | … my family life would be |
| **FT-rec** | زندگی خانوادگی من ....... . |
| **BT1** | My family life …. |
| **BT2** | my family life would be …. |
| **Comments** | AM-16-Oct-2010. BT2 is wrong back translation since it includes the verb. FT-rec does not contain **‘would be’**.  RHUL (AW+RP) 13-Dec-10: Please see ‘global’ issue #1 at the top.  AM-31-May.2011. Please see our previous explanation to this.  RHUL (AW+RP) 6-Oct-11: See our comments of today’s date in the ‘global’ section at the top of the report. |
| **FT rev1** |  |
| **BT rev1** |  |
| **Comments** |  |

| Item | (6a) responses |
| --- | --- |
| **OE** | very much better – much better – a little better – the same – worse |
| **FT-rec** | خیلی بهتر می شد، بهتر می شد، کمی بهتر می شد، فرقی نمی کرد، بدتر می شد |
| **BT1** | Improved so much, improved, almost improved, did not differ, worsened  Became so much better, became better, became a little better, did not change, became worse  Became so much better, became better, became a little better, was not different, became worse |
| **BT2** | Much better, Better, Somewhat better, The same as before, Worse |
| **Comments** | AM-16-Oct-2010. Both BT1 and BT2 are wrong back translations. FT-rec is correct.  RHUL (AW+RP) 13-Dec-10: Please see all the global issues at the top. The scales need to be something that is equivalent ‘**very much better**, much better etc.  AM-31-May-2011. It was revised as suggested.  RHUL (AW+RP) 6-Oct-11: Thank you for the revisions🗸 |
| **FT rev1** | خیلی خیلی بهتر می شد، خیلی بهتر می شد، کمی بهتر می شد، فرقی نمی کرد، بدتر می شد |
| **BT rev1** |  |
| **Comments** |  |

| Item | (6b) statement |
| --- | --- |
| **OE** | My family life is |
| **FT-rec** | زندگی خانوادگی برای من ........ . |
| **BT1** | For me, family life …… |
| **BT2** | For me, family life is…. |
| **Comments** | AM-16-Oct-2010. **‘My’** is back translated as **‘for me’** by both BT1 and BT2. However, this does not differ from OE.  RHUL (AW+RP) 13-Dec-10: It may seem like that until one examines it a bit closer. The potential problem with this is that it now reads (in the BTs) like the person’s objective view of the importance of family life in general for people in general. It needs to be clear that this is subjective and about how important the person’s own family life is to him/herself. Can you please advise whether, in the Farsi, this is absolutely clear? If it isn’t, then you need to find some way of including ‘my’. There’s no problem with leaving in the ‘For me’ if you want to (see the concept guidelines), but the ‘my’ is very important here.  AM-31-May-2011. To include ‘my’ in the statement we suggest to revise the sentence to for instance:  ‘My family life is important for me in’ order to make it more clear in Farsi.  RHUL (AW+RP) 6-Oct-11: OK, thanks🗸 |
| **FT rev1** | زندگی خانوادگی ام برای من......... |
| **BT rev1** |  |
| **Comments** |  |

| Item | (6b) responses |
| --- | --- |
| **OE** | very important – important – somewhat important – not at all important |
| **FT-rec** | خیلی مهم است، مهم است، تا حدی مهم است، اصلا مهم نیست |
| **BT1** | Are very important, are important, are almost important, are not important at all.  Are very important, are important, are almost important, are unimportant. |
| **BT2** | Very important, Important, Somewhat important, Not important at all |
| **Comments** | AM-16-Oct-2010. BT2 is wrong. In BT1 **‘are’** should be **‘is’**. However, FT-rec is acceptable.  RHUL (AW+RP) 13-Dec-10: Please see our comment for item 1b responses concerning *almost*.  AM-12-Sept-2011. Please see our explanation for item 1b.  RHUL (AW+RP) 6-Oct-11: Thank you, the revisions have been explained and ‘somewhat’ is correct.🗸 |
| **FT rev1** |  |
| **BT rev1** |  |
| **Comments** |  |

| Item | (7a) statement |
| --- | --- |
| **OE** | … my friendships and social life would be |
| **FT-rec** | دوستی ها و روابط اجتماعی من ........ . |
| **BT1** | Friendships and my social relationships ….. |
| **BT2** | my friendships and social relationships would be… |
| **Comments** | AM-18-Oct-2010. BT2 is wrong back translation since include the verb **’would be’**. In General BT1 is O.K. However, **‘social life’** was back translated as **‘social relationships’** that is more appropriate in Persian.  RHUL (AW+RP) 13-Dec-10: Please see global issue #1 – otherwise this is OK. 🗸 |
| **FT rev1** |  |
| **BT rev1** |  |
| **Comments** |  |

| Item | (7a) responses |
| --- | --- |
| **OE** | very much better – much better – a little better – the same – worse |
| **FT-rec** | خیلی بهتر می شد، بهتر می شد، کمی بهتر می شد، فرقی نمی کرد، بدتر می شد |
| **BT1** | Improved so much, improved, almost improved, did not differ, worsened  Became so much better, became better, became a little better, did not change, became worse  Became so much better, became better, became a little better, was not different, became worse |
| **BT2** | Much better, Better, Somewhat better, The same as before, Worse |
| **Comments** | AM-16-Oct-2010. Both BT1 and BT2 are wrong back translations. FT-rec is correct.  RHUL (AW+RP) 13-Dec-10: Please see global issue #2: also global issue #3 about ‘very much’.  AM-12-Sept-2011. This was revised as suggested.  RHUL (AW+RP) 6-Oct-11: Thank you for the revision of the response options (relating to the use of *very very*).🗸 |
| **FT rev1** | خیلی خیلی بهتر می شد، خیلی بهتر می شد، کمی بهتر می شد، فرقی نمی کرد، بدتر می شد |
| **BT rev1** |  |
| **Comments** |  |

| Item | (7b) statement |
| --- | --- |
| **OE** | My friendships and social life are |
| **FT-rec** | دوستی ها و روابط اجتماعی برای من ....... . |
| **BT1** | For me, friendships and social relationships …. |
| **BT2** | For me, friendships and social relationships are… |
| **Comments** | AM-18-Oct-2010. **‘My’** is back translated as **‘for me’** by both BT1 and BT2. However, this does not differ from OE. In addition BT2 included **‘are’** in his or her back translation which is wrong since it does not exist in FT-rec.  RHUL (AW+RP) 13-Dec-10: Please see our comments about family life (6b). The same issue about ‘my’ applies here.  AM-12-Sept-2011. The statement was revised: ‘for me’ was removed and instead ‘my’ was replaced.  RHUL (AW+RP) 6-Oct-11: Thank you🗸 |
| **FT rev1** | دوستی ها و روابط اجتماعی ام ......... |
| **BT rev1** |  |
| **Comments** |  |

| Item | (7b) responses |
| --- | --- |
| **OE** | very important – important – somewhat important – not at all important |
| **FT-rec** | خیلی مهم است، مهم است، تا حدی مهم است، اصلا مهم نیست |
| **BT1** | Are very important, are important, are almost important, are not important at all.  Are very important, are important, are almost important, are unimportant. |
| **BT2** | Very important, Important, Somewhat important, Not important at all |
| **Comments** | AM-18-Oct-2010. BT1 is O.K. However, FT-rec should be revised since the verb in OE is **‘are’** but FT-rec uses **‘is’**.  RHUL (AW+RP) 13-Dec-10: Thank you for the correction. However, Please see our comment for item 1b responses concerning *almost*.  AM-12-Sept-2011. Somewhat is exactly exist in Ft-rec.  RHUL (AW+RP) 6-Oct-11:🗸 |
| **FT rev1** | خیلی مهم هستند، مهم هستند، تا حدی مهم هستند، اصلا مهم نیستند. |
| **BT rev1** |  |
| **Comments** |  |

| Item | (8) preliminary question |
| --- | --- |
| **OE** | Do you have or would you like to have a close personal relationship (e.g. husband / wife, partner): |
| **FT-rec** | آیا دارای رابطه نزدیک با همسر خود هستید یا دوست دارید چنین رابطه ای داشته باشید؟ |
| **BT1** | Do you have a close relationship with your husband/ wife or do you like to have such a relationship? |
| **BT2** | Do you have or would you like to have a close relationship with your partner? |
| **Comments** | AM-18-Oct-2010. BT1 is O.K. BT2 is wrong. In general FT-rec is O.K. considering the cultural issues discussed before.  RHUL (AW+RP) 13-Dec-10: We fully understand about the cultural issues. The OE wording has obviously been done to make it as all-embracing as possible in order to cover various cultures, and we recognise that for your culture some adaptation may be needed. However, there is evidently a problem with the wording as it is in the FT-rec. We are asking about whether the person would like to have such a relationship; we are not asking about the quality of an existing relationship, i.e. their relationship with their spouse (that comes in part (a) and is not an open question but a ‘diabetes-dependent’ issue. That is the first point. The second point is that not all respondents will necessarily be married. They might still be single or they might be widowed. The way this is worded at present in the Farsi assumes that everyone is married. As both BTs make this point clearly, the problem must lie in the FT-rec and not in the BTs. The Farsi translation therefore needs to be changed. It may be that for your culture you need to change the wording to something like, ‘Are you married or would you like to be married?’ Please review and advise – and please give us a back translation below of what you suggest.  AM-12-Sept-2011. I think there was a misunderstanding here. According to the comment the preliminary question (Ft-rec) was revised as suggested: Are you married or would you like to be married?  RHUL (AW+RP) 6-Oct-11:🗸 thank you. |
| **FT rev1** | آیا شما ازدواج کرده اید یا دوست دارید که ازدواج کنید؟ |
| **BT rev1** |  |
| **Comments** |  |

| Item | (8a) statement |
| --- | --- |
| **OE** | … my closest personal relationship would be |
| **FT-rec** | این رابطه ....... . |
| **BT1** | This relationship ….  The relationship …. |
| **BT2** | This relationship would be… |
| **Comments** | AM-18-Oct-2010. BT1 is O.K. BT2 is wrong back translation since it includes the verb.  RHUL (AW+RP) 13-Dec-10: If you make the kind of cultural adaptation that we suggest above, you would then need to make a similar one here and perhaps say, ‘… my marital relationship would be’. For anyone who isn’t married, they won’t be answering this question as they will have said No to Q.8 and moved on to item 9. Please review and advise and give us a precise translation of the revised wording.  AM-12-Sept-2011. Exactly! However we think ‘my marital status’ is much closer to what you are suggesting and even more meaningful in Farsi.  RHUL (AW+RP) 6-Oct-11:🗸 OK, if you feel that is better for Iran. |
| **FT rev1** | وضعیت ازدواج من ........ |
| **BT rev1** |  |
| **Comments** |  |

| Item | (8a) responses |
| --- | --- |
| **OE** | very much better – much better – a little better – the same – worse |
| **FT-rec** | خیلی گرم تر می شد، گرم تر می شد، کمی گرم تر می شد، فرقی نمی کرد، کمی سردتر می شد |
| **BT1** | Became much more intimate, became more intimate, became almost more intimate, did not differ, became less intimate |
| **BT2** | Much warmer, Warmer, Somewhat warmer, The same as before, colder |
| **Comments** | AM-18-Oct-2010. BT2 is O.K, except that it did not include the verb in back translation. FT-rec conveys the actual concept of OE.  RHUL (AW+RP) 13-Dec-10: We would prefer not to have ‘warmer’. It might go with the wording you had, but that needs changing anyway and once that is changed, then we should be able to keep ‘better’, as in the OE. We need to remember that this is not about how intimate or warm the relationship is but how it is overall, thinking about how diabetes can impact on a relationship, namely in many ways and not just on the intimate side. In addition, the physical side of marriage is dealt with in the next item as a separate issue (impotence being a potential problem for men with diabetes), so we don’t want to focus on that particular aspect here or we get duplication and possible loss of variability in the scoring across these two items.  AM-12-Sept-2011. The word ‘warmer’ was removed.  RHUL (AW+RP) 6-Oct-11: Could you please show a literal English translation of what the response options now say? |
| **FT rev1** | خیلی خیلی بهتر می شد، خیلی بهتر می شد، کمی بهتر می شد، فرقی نمی کرد، بدتر می شد |
| **BT rev1** |  |
| **Comments** |  |

| Item | (8b) statement |
| --- | --- |
| **OE** | For me, having a close personal relationship is |
| **FT-rec** | برای من داشتن این رابطه ......... . |
| **BT1** | For me, having this relationship …. |
| **BT2** | For me, having this relationship is… |
| **Comments** | AM-18-Oct-2010. In General both BT1 and BT2 are correct except that BT2 wrongly includes verb in his or her back translation.  RHUL (AW+RP) 13-Dec-10: Given the kind of changes we have suggested above, this would then need to read something like, ‘For me, being married is…”  AM-12-Sept-2011. This was revised to: For me, being married….  RHUL (AW+RP) 6-Oct-11:🗸 |
| **FT rev1** | ازواج کردن برای من .......... |
| **BT rev1** |  |
| **Comments** |  |

| Item | (8b) responses |
| --- | --- |
| **OE** | very important – important – somewhat important – not at all important |
| **FT-rec** | خیلی مهم است، مهم است، تا حدی مهم است، اصلا مهم نیست |
| **BT1** | Are very important, are important, are almost important, are not important at all.  Are very important, are important, are almost important, are unimportant. |
| **BT2** | Very important, Important, Somewhat important, Not important at all |
| **Comments** | AM-18-Oct-2010. BT2 is wrong. In BT1 **‘are’** should be **‘is’**. However, FT-rec is acceptable.  RHUL (AW+RP) 13-Dec-10: Please see our comment for item 1b responses concerning *almost* and the verb should be in the singular (and conditional).  AM-12-Sept-2011. Ft-rec satisfies your concern.  RHUL (AW+RP) 6-Oct-11:🗸 |
| **FT rev1** |  |
| **BT rev1** |  |
| **Comments** |  |

| Item | (9) preliminary question |
| --- | --- |
| **OE** | Do you have or would you like to have a sex life? |
| **FT-rec** | آیا شما روابط زناشوئی دارید و یا دوست دارید که چنین رابطه ای داشته باشید؟ |
| **BT1** | Do you have a sexual relationship or do you like to have such a relationship? |
| **BT2** | Do you have or would like to have sexual relations? |
| **Comments** | AM-18-Oct-2010. O.K.  RHUL (AW+RP) 13-Dec-10: 🗸 Thanks, that seems fine. |
| **FT rev1** |  |
| **BT rev1** |  |
| **Comments** |  |

| Item | (9a) statement |
| --- | --- |
| **OE** | … my sex life would be |
| **FT-rec** | روابط زناشوئی من ....... . |
| **BT1** | My sexual relationships …. |
| **BT2** | My sexual relations would be… |
| **Comments** | AM-18-Oct-2010. O.K. (BT2 again included verb in his or her back translation!)  RHUL (AW+RP) 13-Dec-10:🗸 (as commented on already) |
| **FT rev1** |  |
| **BT rev1** |  |
| **Comments** |  |

| Item | (9a) responses |
| --- | --- |
| **OE** | very much better – much better – a little better – the same – worse |
| **FT-rec** | خیلی بهتر می شد، بهتر می شد، کمی بهتر می شد، فرقی نمی کرد، بدتر می شد |
| **BT1** | Improved so much, improved, almost improved, did not differ, worsened  Became so much better, became better, became a little better, did not change, became worse  Became so much better, became better, became a little better, was not different, became worse |
| **BT2** | Much better, Better, Somewhat better, The same as before, Worse |
| **Comments** | AM-18-Oct-2010. Both BT1 and BT2 are wrong back translations. FT-rec is correct.  RHUL (AW+RP) 13-Dec-10: Please see global issue #2: also global issue #3 about ‘very much’.  AM-12-Sept-2011. This was revised as suggested.  RHUL (AW+RP) 6-Oct-11: Thank you for the revisions (relating to *very very*) 🗸 |
| **FT rev1** | خیلی خیلی بهتر می شد، خیلی بهتر می شد، کمی بهتر می شد، فرقی نمی کرد، بدتر می شد |
| **BT rev1** |  |
| **Comments** |  |

| Item | (9b) statement |
| --- | --- |
| **OE** | For me, having a sex life is |
| **FT-rec** | این رابطه برای من ....... . |
| **BT1** | For me, this relationship ….. |
| **BT2** | For me, these relations are…. |
| **Comments** | AM-18-Oct-2010. BT2 included **‘are’** in his or her back translation while it does not exist in FT-rec. FT-rec do not use **‘sex life’** and instead uses **‘this relationship’** as agreed.  RHUL (AW+RP) 13-Dec-10:POK thanks. Please see our comments on P2, global issue #1. |
| **FT rev1** |  |
| **BT rev1** |  |
| **Comments** |  |

| Item | (9b) responses |
| --- | --- |
| **OE** | very important – important – somewhat important – not at all important |
| **FT-rec** | خیلی مهم است، مهم است، تا حدی مهم است، اصلا مهم نیست |
| **BT1** | Are very important, are important, are almost important, are not important at all.  Are very important, are important, are almost important, are unimportant. |
| **BT2** | Very important, Important, Somewhat important, Not important at all |
| **Comments** | AM-18-Oct-2010. BT2 is wrong. In BT1 **‘are’** should be **‘is’**. However, FT-rec is acceptable.  RHUL (AW+RP) 13-Dec-10: Please see our comment for item 1b responses concerning *almost*.  AM-12-Sept-2011. Ft-rec satisfies your concern.  RHUL (AW+RP) 6-Oct-11: Thank you🗸 |
| **FT rev1** |  |
| **BT rev1** |  |
| **Comments** |  |

| Item | (10a) statement |
| --- | --- |
| **OE** | …my physical appearance would be |
| **FT-rec** | به لحاظ ظاهر جسمی ....... . |
| **BT1** | In appearance ….  In physical appearance …. |
| **BT2** | my physical appearance would be… |
| **Comments** | AM-18-Oct-2010. O.K. (BT2 included verb while it does not exist in FT-rec as agreed).  RHUL (AW+RP) 13-Dec-10:🗸 looks fine, with the usual caveat about the place and tense of the verb. |
| **FT rev1** |  |
| **BT rev1** |  |
| **Comments** |  |

| Item | (10a) responses |
| --- | --- |
| **OE** | very much better – much better – a little better – the same – worse |
| **FT-rec** | خیلی بهتر به نظر می رسیدم، بهتر به نظر می رسیدم، کمی بهتر به نظر می رسیدم، فرقی نمی کردم، بدتر به نظر می رسیدم |
| **BT1** | I looked much better, I looked better, I looked almost better, I didn’t differ, I looked |
| **BT2** | Much better, Better, Somewhat better, The same as before, Worse |
| **Comments** | AM-18-Oct-2010. Both back translations are wrong. FT-rec conveys what was agreed during forward translation process.  RHUL (AW+RP) 13-Dec-10: We would not have agreed a past tense for the verb. Same issue as elsewhere – please see global issue #2: also global issue #3 about ‘very much’.  AM-12-Sept-2011. The verb is not past but past progressive as explained before. The responses also were revised as suggested.  RHUL (AW+RP) 6-Oct-11: Where is the past progressive coming from? It is not in the OE and BT2 does not show it at all.  On a different point: please remember to check this item very carefully in the CDs to make sure that people are not reading this in terms of their personal ‘looks’ (i.e. the kind associated with presentation and attractiveness). |
| **FT rev1** | خیلی خیلی بهتر به نظر می رسیدم، خیلی بهتر به نظر می رسیدم، کمی بهتر به نظر می رسیدم، فرقی نمی کردم، بدتر به نظر می رسیدم |
| **BT rev1** |  |
| **Comments** |  |

| Item | (10b) statement |
| --- | --- |
| **OE** | My physical appearance is |
| **FT-rec** | ظاهر جسمی برای من ....... . |
| **BT1** | For me, physical appearance ….. |
| **BT2** | For me, physical appearance is… |
| **Comments** | AM-18-Oct-2010. O.K. (BT2!! Please see previous comments using verbs in response categories).  RHUL (AW+RP) 13-Dec-10: Again, please see item 6b: ‘For me’ is not the same as ‘My’. Please change as necessary.  AM-31-May-2011. To include ‘my’ in the statement we suggest to revise the sentence to for instance:  ‘My physical appearance is important for me in’ order to make it more clear in Farsi.  RHUL (AW+RP) 6-Oct-11:OK, thank you. So the full statement would be  ‘My physical appearance is (very important/important/somewhat important/not at all important) for me.  Is this correct? |
| **FT rev1** | ظاهر جسمی ام برای من ........... |
| **BT rev1** |  |
| **Comments** |  |

| Item | (10b) responses |
| --- | --- |
| **OE** | very important – important – somewhat important – not at all important |
| **FT-rec** | خیلی مهم است، مهم است، تا حدی مهم است، اصلا مهم نیست |
| **BT1** | Are very important, are important, are almost important, are not important at all.  Are very important, are important, are almost important, are unimportant. |
| **BT2** | Very important, Important, Somewhat important, Not important at all |
| **Comments** | AM-18-Oct-2010. BT2 is wrong. In BT1 **‘are’** should be **‘is’**. However, FT-rec is acceptable.  RHUL (AW+RP) 13-Dec-10: Please see our comment for item 1b responses concerning *almost*.  AM-12-Sept-2011. Ft-rec satisfies your concern.  RHUL (AW+RP) 6-Oct-11: Thank you🗸 |
| **FT rev1** |  |
| **BT rev1** |  |
| **Comments** |  |

| Item | (11a) statement |
| --- | --- |
| **OE** | … my self-confidence would be |
| **FT-rec** | اعتماد به نفسم ..... |
| **BT1** | My self-confidence ….. |
| **BT2** | my self confidence would… |
| **Comments** | AM-18-Oct-2010. O.K. (BT2!! Please see previous comments using verbs in response categories).  RHUL (AW+RP) 13-Dec-10:🗸 Re BT2, see global issue #1.  You seem to have ‘my’ here without problem. Please include it elsewhere where we have commented about the difference between ‘my’ and ‘for me’.  AM-12-Sept-2011-Done.  RHUL (AW+RP) 6-Oct-11: Thank you🗸 |
| **FT rev1** | اعتماد به نفسم برای من ............... |
| **BT rev1** |  |
| **Comments** |  |

| Item | (11a) responses |
| --- | --- |
| **OE** | very much better – much better – a little better – the same – worse |
| **FT-rec** | خیلی بیشتر می شد، بیشتر می شد، کمی بیشتر می شد، فرقی نمی کرد، کمتر می شد |
| **BT1** | Became much more, became more, became a bit more, did not differ, became less |
| **BT2** | Increase significantly, Increase, Increase somewhat, Be the same as before, Be less |
| **Comments** | AM-18-Oct.2010. Both back translations are wrong. FT-rec is O.K.  RHUL (AW+RP) 13-Dec-10: Please see global issue #2: also global issue #3 about ‘very much’. If ‘more’ collocates better with self-confidence than ‘better’, then that is OK. Please check the patients are happy with this in the CDs.  AM-12-Sept-2011. Done.  RHUL (AW+RP) 6-Oct-11: 🗸 |
| **FT rev1** | خیلی خیلی بیشتر می شد، خیلی بیشتر می شد، کمی بیشتر می شد، فرقی نمی کرد، کمتر می شد |
| **BT rev1** |  |
| **Comments** |  |

| Item | (11b) statement |
| --- | --- |
| **OE** | My self-confidence is |
| **FT-rec** | اعتماد به نفس برای من ....... . |
| **BT1** | For me, self-confidence ….. |
| **BT2** | For me, self confidence is… |
| **Comments** | AM-18-Oct-2010. O.K. (BT2!! Please see previous comments about using verbs in response categories).  RHUL (AW+RP) 13-Dec-10: Again, we need ‘my’ here, even if you keep ‘For me’ and have both (it has been done that way in some languages for some items).  AM-31-May-2011. To include ‘my’ in the statement we suggest to revise the sentence to for instance:  ‘My self-confidence is important for me in’ order to make it more clear in Farsi.  RHUL (AW+RP) 6-Oct-11: OK thanks🗸 |
| **FT rev1** | اعتماد به نفسم برای من .......... |
| **BT rev1** |  |
| **Comments** |  |

| Item | (11b) responses |
| --- | --- |
| **OE** | very important – important – somewhat important – not at all important |
| **FT-rec** | خیلی مهم است، مهم است، تا حدی مهم است، اصلا مهم نیست |
| **BT1** | Are very important, are important, are almost important, are not important at all.  Are very important, are important, are almost important, are unimportant. |
| **BT2** | Very important, Important, Somewhat important, Not important at all |
| **Comments** | AM-18-Oct-2010. BT2 is wrong. In BT1 **‘are’** should be **‘is’**. However, FT-rec is acceptable.  RHUL (AW+RP) 13-Dec-10: Please see our comment for item 1b responses concerning *almost*.  AM-12-Sept-2011. Ft-rec satisfies your concern.  RHUL (AW+RP) 6-Oct-11: Thank you🗸 |
| **FT rev1** |  |
| **BT rev1** |  |
| **Comments** |  |

| Item | (12a) statement |
| --- | --- |
| **OE** | … my motivation would be |
| **FT-rec** | انگیزه ام ..... . |
| **BT1** | My motivation ….. |
| **BT2** | my motivation would… |
| **Comments** | AM-18-Oct-2010. O.K. (BT2!! Please see previous comments about using verbs in response categories).  RHUL (AW+RP) 13-Dec-10: Please see global issue #1 – otherwise this is OK.  RHUL (AW+RP) 6-Oct-11:🗸 |
| **FT rev1** |  |
| **BT rev1** |  |
| **Comments** |  |

| Item | (12a) responses |
| --- | --- |
| **OE** | very much better – much better – a little better – the same – worse |
| **FT-rec** | خیلی بیشتر می شد، بیشتر می شد، کمی بیشتر می شد، فرقی نمی کرد، کمتر می شد |
| **BT1** | Became much more, became more, became a bit more, did not differ, became less |
| **BT2** | Increase significantly, Increase, Increase somewhat, Be the same as before, Be less |
| **Comments** | AM-18-Oct.2010. Both back translations are wrong. FT-rec is O.K.  RHUL (AW+RP) 13-Dec-10: Please see global issue #2: also global issue #3 about ‘very much’. If ‘more’ collocates better with motivation in your language, then that is OK. Please check the patients are comfortable with this combination.  AM-12-Sept-2011. Done.  RHUL (AW+RP) 6-Oct-11:🗸 |
| **FT rev1** | خیلی خیلی بیشتر می شد، خیلی بیشتر می شد، کمی بیشتر می شد، فرقی نمی کرد، کمتر می شد |
| **BT rev1** |  |
| **Comments** |  |

| Item | (12b) statement |
| --- | --- |
| **OE** | My motivation is |
| **FT-rec** | داشتن انگیزه برایم ...... . |
| **BT1** | For me, having motivation ….. |
| **BT2** | For me, having motivation is… |
| **Comments** | AM-18-Oct-2010. BT2 is wrong. In BT2 **‘is’** included in back translated statement and not in statement (12b). [BT2 wrongly included word **‘is’** in his/her translation]. BT1 is acceptable.  RHUL (AW+RP) 13-Dec-10: This is more complex than the OE now, but if that is how it has to be then OK. However, please be sure about how this will be interpreted. It is the same issue again, namely ‘For me’ and ‘my’ are not the same, even if you say ‘For me, having motivation…’ It may be that for this particular item it will work OK, but please check carefully to be sure (and perhaps get someone else to read it and tell you what it is saying to them).  AM-12-Sept-2011. Translation of motivation to Farsi is possible but very difficult to understand by ordinary people. When we use it with having then it is more likely to be understood by everyone. We checked the issue with other colleagues and they also confirmed that at present form it reads much better. In summary as you indicated in this particular item perhaps it will work OK.  RHUL (AW+RP) 6-Oct-11: OK, thank you for checking this and for your explanation. 🗸 |
| **FT rev1** |  |
| **BT rev1** |  |
| **Comments** |  |

| Item | (12b) responses |
| --- | --- |
| **OE** | very important – important – somewhat important – not at all important |
| **FT-rec** | خیلی مهم است، مهم است، تا حدی مهم است، اصلا مهم نیست |
| **BT1** | Are very important, are important, are almost important, are not important at all.  Are very important, are important, are almost important, are unimportant. |
| **BT2** | Very important, Important, Somewhat important, Not important at all |
| **Comments** | AM-18-Oct-2010. BT2 is wrong. In BT1 **‘are’** should be **‘is’**. However, FT-rec is acceptable.  RHUL (AW+RP) 13-Dec-10: Please see our comment for item 1b responses concerning *almost*.  AM-13-Sept-2011. Ft-rec satisfies your concern.  RHUL (AW+RP) 6-Oct-11:🗸 |
| **FT rev1** |  |
| **BT rev1** |  |
| **Comments** |  |

| Item | (13a) statement |
| --- | --- |
| **OE** | … the way people in general react to me would be |
| **FT-rec** | در کل، برخورد مردم نسبت به من ........ . |
| **BT1** | In general, peoples treatment toward me……. |
| **BT2** | the way people treat me would be… |
| **Comments** | AM-19-Oct-2010. BT2 is wrong back translation since it includes the verb. BT1 is a better translation of FT-rec  RHUL (AW+RP) 13-Dec-10: Please see global issue #1.  Both BT1 and 2 have used ‘treat/ment’ which is slightly different to ‘react’, suggesting the Farsi is not a correct translation (of ‘react’). Is it possible that the same Farsi word means both ‘react’ and ‘treat’ or ‘behave towards’? Please advise. If not, then could you find another, better word for ‘react’, without it being too strong? May be you can find a word more easily once you have read our suggestion immediately below about dropping ‘in general’. Would this make a difference? Please review and advise.  The way BT1 has translated this suggests that the FT-rec is wrong about ‘in general’, i.e. the implication is that the ‘In general’ is related to the treatment the person receives from ‘people’ (‘people’s treatment toward me’). It should qualify ‘people’, not ‘react’ (or as here, ‘treat’) – please see the guidelines. BT2 appears to have ignored the ‘in general’, possibly because it is not clear from the Farsi where it should go and what it should qualify, and perhaps the translator has taken the ‘safest’ route in order to avoid any misinterpretation. As we say in the guidelines, if there is any doubt, please just drop the ‘in general’ (which is what BT2 has done) – this would not be a problem and would probably be the best and simplest solution.  AM-13-Sept-2011. The word ‘برخورد’ was changed to ‘واکنش’ for a better translation of OE. ‘In general’ was also removed from the FT-rec .  RHUL (AW+RP) 6-Oct-11: Thank you. Could you please add below a literal back translation of the new wording. |
| **FT rev1** | واکنش مردم نسبت به من .............. |
| **BT rev1** |  |
| **Comments** |  |

| Item | (13a) responses |
| --- | --- |
| **OE** | very much better – much better – a little better – the same – worse |
| **FT-rec** | خیلی بهتر می شد، بهتر می شد، کمی بهتر می شد، فرقی نمی کرد، بدتر می شد |
| **BT1** | Improved so much, improved, almost improved, did not differ, worsened  Became so much better, became better, became a little better, did not change, became worse  Became so much better, became better, became a little better, was not different, became worse |
| **BT2** | Much better, Better, Somewhat better, The same as before, Worse |
| **Comments** | AM-19-Oct-2010. Both BT1 and BT2 are wrong back translations. FT-rec is correct.  RHUL (AW+RP) 13-Dec-10: Please see global issue #2: also global issue #3 about ‘very much’.  AM-13-Sept-2011. Done.  RHUL (AW+RP) 6-Oct-11:🗸 |
| **FT rev1** | خیلی خیلی بهتر می شد، خیلی بهتر می شد، کمی بهتر می شد، فرقی نمی کرد، بدتر می شد |
| **BT rev1** |  |
| **Comments** |  |

| Item | (13b) statement |
| --- | --- |
| **OE** | The way people in general react to me is |
| **FT-rec** | چگونگی برخورد مردم نسبت به من برایم ....... . |
| **BT1** | For me, the way people treat me….. |
| **BT2** | For me, the way people treat me is… |
| **Comments** | AM-19-Oct-2010. Both BT1 and BT2 used the word **‘treat’** instead of **‘react’**. BT2 wrongly included word **‘is’** in his/her translation. However, FT-rec was revised and **‘برخورد’** was changed to **‘واکنش‘**. In addition **‘in general’** is missing in FT-rec. This also was added to the statement.  RHUL (AW+RP) 13-Dec-10: Please explain the English meaning of the word replaced and the meaning of the new word. Are you saying that because both BTs used ‘treat’, you have corrected the FT-rec to ‘react’? What does FT-rec say now?  Please see our comment in 13a above about dropping ‘in general’ and advise if you agree with this or prefer to try to change the sentence so that it clearly qualifies ‘people’.  AM-13-Sept-2011. Both words have similar meaning but ‘واکنش’ is a more closer meaning for 'react'. 'In general' was removed from the statement as suggested.  RHUL (AW+RP) 6-Oct-11: Please could you give the English version of your revision. Many thanks. |
| **FT rev1** | عموما چگونگی واکنش مردم نسبت به من برایم ........... .  چگونگی واکنش مردم نسبت به من برایم ........... . |
| **BT rev1** |  |
| **Comments** |  |

| Item | (13b) responses |
| --- | --- |
| **OE** | very important – important – somewhat important – not at all important |
| **FT-rec** | خیلی مهم است، مهم است، تا حدی مهم است، اصلا مهم نیست |
| **BT1** | Are very important, are important, are almost important, are not important at all.  Are very important, are important, are almost important, are unimportant. |
| **BT2** | Very important, Important, Somewhat important, Not important at all |
| **Comments** | AM-19-Oct-2010. BT2 is wrong. In BT1 **‘are’** should be **‘is’**. However, FT-rec is acceptable.  RHUL (AW+RP) 13-Dec-10: Please see our comment for item 1b responses concerning *almost*.  AM-13-Sept-2011. Ft-rec satisfies your concern.  RHUL (AW+RP) 6-Oct-11:🗸 |
| **FT rev1** |  |
| **BT rev1** |  |
| **Comments** |  |

| Item | (14a) statement |
| --- | --- |
| **OE** | … my feelings about the future (e.g. worries, hopes) would be |
| **FT-rec** | احساساتم درباره آینده مثل احساس نگرانی یا امید به آینده ....... . |
| **BT1** | My feelings about future such as concern or hope about future ……  My feelings about future including being concern or hopeful about future ….. |
| **BT2** | my feelings about the future such as my worries or hopes would be… |
| **Comments** | AM-19-Oct-2010. BT2 is wrong back translation since it includes the verb. BT1 is a better back translation of FT-rec.  RHUL (AW+RP) 13-Dec-10: Please see global issue #1 – this is OK. Can you just tell us for the record: do you not use an abbreviation like ‘e.g.’ (standing for ‘for example’)? Do you always put ‘such as’? It’s always helpful for us to learn these small details about other languages we’re working with.  AM-13-Sept-2011. Yes, we do not have abbreviation like ‘e.g.’ and always put ‘such as’ instead.  RHUL (AW+RP) 6-Oct-11: Thanks for explaining. 🗸 |
| **FT rev1** |  |
| **BT rev1** |  |
| **Comments** |  |

| Item | (14a) responses |
| --- | --- |
| **OE** | very much better – much better – a little better – the same – worse |
| **FT-rec** | خیلی بهتر می شد، بهتر می شد، کمی بهتر می شد، فرقی نمی کرد، بدتر می شد |
| **BT1** | Improved so much, improved, almost improved, did not differ, worsened  Became so much better, became better, became a little better, did not change, became worse  Became so much better, became better, became a little better, was not different, became worse |
| **BT2** | Much better, Better, Somewhat better, The same as before, Worse |
| **Comments** | AM-19-Oct-2010. Both BT1 and BT2 are wrong back translations. FT-rec is correct.  RHUL (AW+RP) 13-Dec-10: Please see global issue #2: also global issue #3 about ‘very much’.  AM-13-Sept-2011. Done  RHUL (AW+RP) 6-Oct-11: Same global comments (of today’s date) as mentioned in previous items. Meantime thanks for changing to *very very...* which we think you have done below. |
| **FT rev1** | خیلی خیلی بهتر می شد، خیلی بهتر می شد، کمی بهتر می شد، فرقی نمی کرد، بدتر می شد |
| **BT rev1** |  |
| **Comments** |  |

| Item | (14b) statement |
| --- | --- |
| **OE** | My feelings about the future are |
| **FT-rec** | احساسی که نسبت به آینده دارم، برایم ....... . |
| **BT1** | For me, my feeling about future …… |
| **BT2** | For me, my feelings about the future are… |
| **Comments** | AM-19-Oct-2010. In general both BT1 and BT2 are correct back translations except for existence of verb in BT2! However, it was felt that for a better reflection of OE, FT-rec should be revised.  RHUL (AW+RP) 13-Dec-10: The BTs both look fine. Could you therefore please explain exactly what you felt was wrong with the existing FT-rec? Please insert a BT of your new wording below (you will see that we provide a place for “BT rev1”). We need to know the meaning of the “FT rev1” and how it differs from the FT‑rec.  AM-13-Sept-2011. The problem was in translation of feelings. It, in previous FT-rec, was translated to feeling (singular) while it should have been plural. |
| **FT rev1** | احساساتم نسبت به آینده برایم........ . |
| **BT rev1** | For me, my feelings about future ………. |
| **Comments** | RHUL (AW+RP) 6-Oct-11: Thanks for including the BT of rev1 here. |

| Item | (14b) responses |
| --- | --- |
| **OE** | very important – important – somewhat important – not at all important |
| **FT-rec** | خیلی مهم است، مهم است، تا حدی مهم است، اصلا مهم نیست |
| **BT1** | Are very important, are important, are almost important, are not important at all.  Are very important, are important, are almost important, are unimportant. |
| **BT2** | Very important, Important, Somewhat important, Not important at all |
| **Comments** | AM-19-Oct-2010. BT1 is O.K. but 'are' in BT1 should be 'is'.  RHUL (AW+RP) 13-Dec-10: Please see our comment for item 1b responses concerning *almost*.  AM-13-Sept-2011. Ft-rec satisfies your concern.  RHUL (AW+RP) 6-Oct-11:🗸 |
| **FT rev1** |  |
| **BT rev1** |  |
| **Comments** |  |

| Item | (15a) statement |
| --- | --- |
| **OE** | … my financial situation would be |
| **FT-rec** | وضع مالی من ........ |
| **BT1** | My financial situation ……. |
| **BT2** | my financial situation would be… |
| **Comments** | AM-19-Oct-2010. BT2 is wrong back translation since it includes the verb. BT1 is acceptable.  RHUL (AW+RP) 13-Dec-10: Please see global issue #1 – otherwise this is OK. 🗸 |
| **FT rev1** |  |
| **BT rev1** |  |
| **Comments** |  |

| Item | (15a) responses |
| --- | --- |
| **OE** | very much better – much better – a little better – the same – worse |
| **FT-rec** | خیلی بهتر می شد، بهتر می شد، کمی بهتر می شد، فرقی نمی کرد، بدتر می شد |
| **BT1** | Improved so much, improved, almost improved, did not differ, worsened  Became so much better, became better, became a little better, did not change, became worse  Became so much better, became better, became a little better, was not different, became worse |
| **BT2** | Much better, Better, Somewhat better, The same as before, Worse |
| **Comments** | AM-19-Oct-2010. Both BT1 and BT2 are wrong back translations. FT-rec is correct.  RHUL (AW+RP) 13-Dec-10: Please see global issue #2: also global issue #3 about ‘very much’.  AM-13-Sept-2011. Done  RHUL (AW+RP) 6-Oct-11:🗸 |
| **FT rev1** | خیلی خیلی بهتر می شد، خیلی بهتر می شد، کمی بهتر می شد، فرقی نمی کرد، بدتر می شد |
| **BT rev1** |  |
| **Comments** |  |

| Item | (15b) statement |
| --- | --- |
| **OE** | My financial situation is |
| **FT-rec** | وضعیت مالی برایم ....... . |
| **BT1** | For me, the financial situation …… |
| **BT2** | For me, my financial situation is… |
| **Comments** | AM-19-Oct-2010. BT2 is wrong back translation since it includes the verb. BT1 is O.K.  RHUL (AW+RP) 13-Dec-10: If you wish to include ‘For me’, that is fine, as long as you also include ‘my’. The ‘For me’ isn’t essential, but the ‘my’ is. Is it in the FT-rec? BT2 suggests it is; BT1 suggests it isn’t., in which case it needs to be added. Please advise which BT is correct (ignoring the matter of the verb, which we have explained at the top).  AM-13-Sept-2011. FT-rec was revised as suggested.  RHUL (AW+RP) 6-Oct-11: Please could you give us the English for this in BTrev1 below. Many thanks |
| **FT rev1** | وضعیت مالی ام برای من............ |
| **BT rev1** |  |
| **Comments** |  |

| Item | (15b) responses |
| --- | --- |
| **OE** | very important – important – somewhat important – not at all important |
| **FT-rec** | خیلی مهم است، مهم است، تا حدی مهم است، اصلا مهم نیست |
| **BT1** | Are very important, are important, are almost important, are not important at all.  Are very important, are important, are almost important, are unimportant. |
| **BT2** | Very important, Important, Somewhat important, Not important at all |
| **Comments** | AM-19-Oct-2010. BT2 is wrong. In BT1 **‘are’** should be **‘is’**. However, FT-rec is acceptable.  RHUL (AW+RP) 13-Dec-10: Are you saying that the verb is in the singular (i.e. to fit ‘financial situation’)? Meantime please also see our comment for item 1b responses concerning *almost*.  AM-13-Sept-2011. Yes.  Regarding the word 'almost', Ft-rec satisfies your concern.  RHUL (AW+RP) 6-Oct-11:🗸 |
| **FT rev1** |  |
| **BT rev1** |  |
| **Comments** |  |

| Item | (16a) statement |
| --- | --- |
| **OE** | … my living conditions would be |
| **FT-rec** | شرایط زندگی من ....... . |
| **BT1** | My life condition …… |
| **BT2** | my living conditions would be… |
| **Comments** | AM-19-Oct-2010. BT2 is wrong back translation since it includes the verb. However, BT1 is a better back translation, although 'conditions' was back translated as condition.  RHUL (AW+RP) 13-Dec-10: Please see global issue #1 – otherwise this is OK. 🗸 |
| **FT rev1** |  |
| **BT rev1** |  |
| **Comments** |  |

| Item | (16a) responses |
| --- | --- |
| **OE** | very much better – much better – a little better – the same – worse |
| **FT-rec** | خیلی بهتر می شد، بهتر می شد، کمی بهتر می شد، فرقی نمی کرد، بدتر می شد |
| **BT1** | Improved so much, improved, almost improved, did not differ, worsened  Became so much better, became better, became a little better, did not change, became worse  Became so much better, became better, became a little better, was not different, became worse |
| **BT2** | Much better, Better, Somewhat better, The same as before, Worse |
| **Comments** | AM-19-Oct-2010. Both BT1 and BT2 are wrong back translations. FT-rec is correct.  RHUL (AW+RP) 13-Dec-10: Please see global issue #2: also global issue #3 about ‘very much’?  AM-13-Sept-2011. Done  RHUL (AW+RP) 6-Oct-11:🗸 |
| **FT rev1** | خیلی خیلی بهتر می شد، خیلی بهتر می شد، کمی بهتر می شد، فرقی نمی کرد، بدتر می شد |
| **BT rev1** |  |
| **Comments** |  |

| Item | (16b) statement |
| --- | --- |
| **OE** | My living conditions are |
| **FT-rec** | شرایط زندگی برایم ....... . |
| **BT1** | For me, life condition …… |
| **BT2** | For my, my living conditions are… |
| **Comments** | AM-19-Oct-2010. BT2 is wrong back translation since it includes the verb. BT1 is O.K., although conditions was back translated as condition.  RHUL (AW+RP) 13-Dec-10: BT2’s ‘living conditions’ is a correct translation. Please also confirm that BT2 is correct in putting not just ‘For me’ (ignoring the typo, ‘my’), but also ‘my living conditions’. As we mentioned above, the ‘For me’ is optional; the ‘my’ before the ‘living conditions’ is essential. Please clarify which is right on this point, BT1 or BT2, i.e. whether ‘my’ is in the FT-rec.  AM-13-Sept-2011. BT2 includes ‘are’ while it does not exist in FT-rec. but in according to ‘my’ it is right. This was revised as suggested.  RHUL (AW+RP) 6-Oct-11: Thank you 🗸 |
| **FT rev1** | شرایط زندگیم برای من ................ |
| **BT rev1** |  |
| **Comments** |  |

| Item | (16b) responses |
| --- | --- |
| **OE** | very important – important – somewhat important – not at all important |
| **FT-rec** | خیلی مهم است، مهم است، تا حدی مهم است، اصلا مهم نیست |
| **BT1** | Are very important, are important, are almost important, are not important at all.  Are very important, are important, are almost important, are unimportant. |
| **BT2** | Very important, Important, Somewhat important, Not important at all |
| **Comments** | AM-19-Oct-2010. BT2 is wrong. In BT1 **‘are’** should be **‘is’**. However, FT-rec is acceptable.  RHUL (AW+RP) 13-Dec-10: Please see our comment for item 1b responses concerning *almost*.  AM-13-Sept-2011. Ft-rec satisfies your concern.  RHUL (AW+RP) 6-Oct-11:🗸 |
| **FT rev1** |  |
| **BT rev1** |  |
| **Comments** |  |

| Item | (17a) statement |
| --- | --- |
| **OE** | … I would have to depend on others when I do not want to |
| **FT-rec** | وابستگی به دیگران زمانی که تمایل ندارم، ....... . |
| **BT1** | Depending on others when I dislike it, …. |
| **BT2** | my dependence on others when I do not want to be dependent would…. |
| **Comments** | AM-19-Oct-2010. BT1 is O.K.  RHUL (AW+RP) 13-Dec-10: This looks good – and it can be a difficult one to translate. Thanks. 🗸 |
| **FT rev1** |  |
| **BT rev1** |  |
| **Comments** |  |

| Item | (17a) responses |
| --- | --- |
| **OE** | very much less – much less – a little less – the same – more |
| **FT-rec** | خیلی کمتر می شد، کمتر می شد، کمی کمتر می شد، فرقی نمی کرد، بیشتر می شد |
| **BT1** | Was much less, was less, was a bit less, was more |
| **BT2** | Decrease a lot, Decrease, Decrease somewhat, Be the same as before, Increase |
| **Comments** | AM-19-Oct-2010. Both BT1 and BT2 are wrong back translation. FT-rec. is O.K.  RHUL (AW+RP) 13-Dec-10: You don’t suggest how this might be improved. This should be the same as several earlier items. Please see global issue #2: also global issue #3 about ‘very much’.  AM-13-Sept-2011. Done  RHUL (AW+RP) 6-Oct-11: Please could you show the BT of this in BTrev1 below. |
| **FT rev1** | خیلی خیلی کمتر می شد، خیلی کمتر می شد، کمی کمتر می شد، فرقی نمی کرد، بیشتر می شد |
| **BT rev1** |  |
| **Comments** |  |

| Item | (17b) statement |
| --- | --- |
| **OE** | For me, not having to depend on others is |
| **FT-rec** | مستقل بودن برای من ....... . |
| **BT1** | For me, being independent ….. |
| **BT2** | For me, being independent is… |
| **Comments** | AM-19-Oct-2010. BT2 includes **‘is’** while it does not exist in FT-rec! BT1 is acceptable.  RHUL (AW+RP) 13-Dec-10: Unfortunately both BTs show that the FT-rec is incorrect – it’s the wrong way round. The translation talks about being “independent” when it should be about “not having to be dependent”. The current Farsi clearly doesn’t have it this way round and doesn’t include the ‘having to’. There is a difference.  This aspect of the statement, making it clear that this is forced dependence is key to what the patients have told us. We recognise that this may be a difficult construction but it does need to be changed, otherwise the question is different from other languages. Patients have said to us that sometimes they don’t mind depending on other people for some things; it is the fact that they have to depend on others that impacts on their QoL, e.g. night-time hypos.  Please re-word this the correct way round. If the *having to* is difficult, you could if necessary say …*not being forced to depend on others…* etc.  AM-13-Sept-2011. This was revised to: for me not being forced to depend on others  RHUL (AW+RP) 6-Oct-11: Thank you 🗸 |
| **FT rev1** | مجبور نبودن برای وابستگی به دیگران برای من............ |
| **BT rev1** |  |
| **Comments** |  |

| Item | (17b) responses |
| --- | --- |
| **OE** | very important – important – somewhat important – not at all important |
| **FT-rec** | خیلی مهم است، مهم است، تا حدی مهم است، اصلا مهم نیست |
| **BT1** | Are very important, are important, are almost important, are not important at all.  Are very important, are important, are almost important, are unimportant. |
| **BT2** | Very important, Important, Somewhat important, Not important at all |
| **Comments** | AM-19-Oct-2010. BT2 is wrong. In BT1 **‘are’** should be **‘is’**. However, FT-rec is acceptable.  RHUL (AW+RP) 13-Dec-10: Please see our comment for item 1b responses concerning *almost*.  AM-13-Sept-2011. Ft-rec satisfies your concern.  RHUL (AW+RP) 6-Oct-11:🗸 |
| **FT rev1** |  |
| **BT rev1** |  |
| **Comments** |  |

| Item | (18a) statement |
| --- | --- |
| **OE** | … my freedom to eat as I wish would be |
| **FT-rec** | آزاد بودم هر آنچه را که می خواهم، بخورم. |
| **BT1** | I was free to eat whatever I wanted |
| **BT2** | If I did not have diabetes and were free to eat whatever I wanted, I would… |
| **Comments** | AM-20-Oct-2010. BT2 is very interesting! He or she uses **‘diabetes’** and **‘would’** while FT-rec does not uses these words at all. BT1 is O.K. and correctly reflects FT-rec. However considering the FT-rec and response categories [(18a) responses-below] it seems that the format of statement should be revised and the word **‘free’** or **‘freedom’** should be removed since **‘freedom’** is included in response categories as agreed.  RHUL (AW+RP) 13-Dec-10: For some reason here BT2 has just included the beginning of the statement (it’s perfectly correct, just not necessary in this report). Please also see global issue #1 concerning the verb. Please also see  Both BTs only cover ‘whatever’ and do not mention ‘whenever’, both of which are encompassed in ‘as I wish’. The FT report states -*As wish was translated exactly and included in the translation.* It looks as if this was not quite correct. Please see the guidelines: if you can’t say ‘as I wish’, then you need to say ‘whatever and whenever’ otherwise an important semantic element if the ‘as I wish’ is omitted.  Please add ‘and whenever’. The basic structure and wording of this item should be the same as for the ‘eat as I wish’ item.  Unfortunately you don’t say how the statement now reads since you have removed *free / freedom*. If you could stay closer to the original OE wording, could you use *freedom* as a noun, and then use the conditional of the verb ‘to be’ with *greater* etc in the responses, so the responses would read, *would be very much greater…* etc – or is that not possible in Farsi? Again, we appreciate that the different structures of the two languages may mean you have to approach this differently. You have changed it below, but we don’t know what this now says. Could you please give us a BT of rev1 below.  AM-13-Sept-2011. This was revised as: My freedom would be very much greater to eat whatever and whenever I wanted. : Here since the responses are at the beginning of the statement, it is impossible in Farsi to start the sentence with would be very much greater.  RHUL (AW+RP) 6-Oct-11: Please could you show the English for the full statement below, including response options. Many thanks. |
| **FT rev1** | .......... هر آنچه را که می خواهم، بخورم.  .......... هر آنچه را، هر وقت که می خواهم بخورم. |
| **BT rev1** | ………….. to eat whatever and whenever I want. |
| **Comments** |  |

| Item | (18a) responses |
| --- | --- |
| **OE** | very much greater – much greater – a little greater – the same – less |
| **FT-rec** | خیلی آزاد بودم، آزاد بودم، کمی آزاد بودم، فرقی نمی کرد، آزاد نبودم |
| **BT1** | I was very free, I was free, I was almost free, it didn’t differ, I was not free |
| **BT2** | Be very free, Be free, Be Somewhat free, Be the same as before, Would not be free |
| **Comments** | AM-20-Oct-2010. O.K.  RHUL (AW+RP) 13-Dec-10: Please see global issue #2: also global issue #3 about ‘very much’. And when you have reviewed the wording above for the 18a statement, you may need to change these response options anyway. If so, please provide not just the new FT rev1, but the BT of rev1 as well.  AM-13-Sept-2011. Just the intensity was considered as suggested.  RHUL (AW+RP) 6-Oct-11: Thanks 🗸 |
| **FT rev1** | خیلی خیلی آزاد بودم، خیلی آزاد بودم، کمی آزاد بودم، فرقی نمی کرد، آزاد نبودم |
| **BT rev1** | I was very very free, I was very free, I was a little free, the same, I was not free |
| **Comments** |  |

| Item | (18b) statement |
| --- | --- |
| **OE** | My freedom to eat as I wish is |
| **FT-rec** | آزاد بودن در خوردن چیزهائی که دوست دارم، برای من ..... |
| **BT1** | For me, being free in eating what I liked …. |
| **BT2** | For me, having the freedom to eat whatever I like is… |
| **Comments** | AM-20-Oct-2010. The tense in BT1 is wrong. The verb **‘is’** in BT2 does not exist in FT-rec.  RHUL (AW+RP) 13-Dec-10: We can see that with this item, ‘my’ might be difficult to include as you have changed ‘freedom’ to ‘being free/having the freedom…’. It needs to be clear that this is about the respondent’s freedom to eat… etc and not the respondent expressing an opinion about the principle of people being free to eat as they wish. Would your ‘For me’ be adequate to make this sufficiently clear? Otherwise we are likely to get different scoring. Please review and advise. Please could you show the additional aspect of ‘whenever’ in the FT rev1, plus the BT of rev1?  AM-13-Sept-2011. Whenever was added.  RHUL (AW+RP) 6-Oct-11: thanks 🗸 |
| **FT rev1** | آزاد بودن در خوردن هر چیز و هر زمانی که می خواهم، برای من ............. |
| **BT rev1** | For me, freedom to eat whatever and whenever I want………….. |
| **Comments** |  |

| Item | (18b) responses |
| --- | --- |
| **OE** | very important – important – somewhat important – not at all important |
| **FT-rec** | خیلی مهم است، مهم است، تا حدی مهم است، اصلا مهم نیست |
| **BT1** | Are very important, are important, are almost important, are not important at all.  Are very important, are important, are almost important, are unimportant. |
| **BT2** | Very important, Important, Somewhat important, Not important at all |
| **Comments** | AM-20-Oct-2010. BT2 is wrong. In BT1 **‘are’** should be **‘is’**. However, FT-rec is acceptable.  RHUL (AW+RP) 13-Dec-10: Please see our comment for item 1b responses concerning *almost*.  AM-13-Sept-2011. Ft-rec satisfies your concern.  RHUL (AW+RP) 6-Oct-11:🗸 |
| **FT rev1** |  |
| **BT rev1** |  |
| **Comments** |  |

| Item | (19a) statement |
| --- | --- |
| **OE** | … my freedom to drink as I wish (e.g. fruit juice, alcohol, sweetened hot and cold drinks) would be |
| **FT-rec** | آزاد بودم هر آنچه را که می خواهم، بنوشم (مثل آب میوه و نوشیدنی های شیرین سرد و گرم) |
| **BT1** | I was free to drink whatever I wanted (such as fruit juice and sweet warm and cold drinks) |
| **BT2** | If I did not have diabetes and were free to drink whatever I wanted (such as fruit juice and sweetened cold and hot beverages), I would… |
| **Comments** | AM-20-Oct-2010. BT2 is very interesting! He or she uses **‘diabetes’** and **‘would’** while FT-rec does not uses these words at all. BT1 is O.K. and correctly reflects FT-rec. However considering the FT-rec and response categories [(19a) responses-below] it seems that the format of statement should be revised and the word **‘free’** or **‘freedom’** should be removed since **‘freedom’** is included in response categories as agreed.  RHUL (AW+RP) 13-Dec-10: Again, for some reason here BT2 has just included the beginning of the statement (it’s perfectly correct, just not necessary in this report).  Also again, as with 18a, both BTs only cover ‘whatever’ and do not mention ‘whenever’, both of which are encompassed in ‘as I wish’. The FT report states -*As wish was translated exactly and included in the translation.* Please add ‘and whenever’. The basic structure and wording of this item should be the same as for the ‘eat as I wish’ item;  You have changed it below, but we don’t know what this now says. Could you please give us a BT of rev1 below.  AM-13-Sept-2011. This was revised as: My freedom would be very much greater to drink whatever and whenever I wanted. : Here since the responses are at the beginning of the statement, it is impossible in Farsi to start the sentence with would be very much greater.  RHUL (AW+RP) 6-Oct-11:Please could you show the full English statement with response options. Thank you |
| **FT rev1** | ......... هر آنچه را که می خواهم، بنوشم (مثل آب میوه و نوشیدنی های شیرین سرد و گرم)  .......... هر آنچه را هر وقت که می خواهم، بنوشم (مثل آب میوه و نوشیدنی های شیرین سرد و گرم) |
| **BT rev1** | ………. to drink whatever and whenever I want (such as fruit juice and sweet warm and cold drinks). |
| **Comments** |  |

| Item | (19a) responses |
| --- | --- |
| **OE** | very much greater – much greater – a little greater – the same – less |
| **FT-rec** | خیلی آزاد بودم، آزاد بودم، کمی آزاد بودم، فرقی نمی کرد، آزاد نبودم |
| **BT1** | I was very free, I was free, I was almost free, it didn’t differ, I was not free |
| **BT2** | Be very free, Be free, Be Somewhat free, Be the same as before, Would not be free |
| **Comments** | AM-20-Oct-2010. O.K.  RHUL (AW+RP) 13-Dec-10: Please see global issue #2: also global issue #3 about ‘very much’. And when you have reviewed the wording above (as for the 18a statement), you may need to change these response options anyway. If so, please provide not just the new FT rev1, but the BT of rev1 as well.  AM-13-Sept-2011. Done.  RHUL (AW+RP) 6-Oct-11: thanks. 🗸 |
| **FT rev1** | خیلی خیلی آزاد بودم، خیلی آزاد بودم، کمی آزاد بودم، فرقی نمی کرد، آزاد نبودم |
| **BT rev1** | I was very very free, I was very free, I was a little free, the same, I was not free |
| **Comments** |  |

| Item | (19b) statement |
| --- | --- |
| **OE** | My freedom to drink as I wish is |
| **FT-rec** | آزاد بودن در نوشیدن هر چیز و هر زمانی که دوست دارم، برای من ....... . |
| **BT1** | For me, being free in drinking what I liked ….. |
| **BT2** | For me, having the freedom to drink whatever I like is… |
| **Comments** | AM-20-Oct-2010. The tense in BT1 is wrong. The verb **‘is’** in BT2 does not exist in FT-rec.  RHUL (AW+RP) 13-Dec-10: As for 18b, we can see that with this item, ‘my’ might be difficult to include as you have changed ‘freedom’ to ‘being free/having the freedom…’. It needs to be clear that this is about the respondent’s freedom to eat… etc and not the respondent expressing an opinion about the principle of people being free to eat as they wish. Would your ‘For me’ be adequate to make this sufficiently clear? Otherwise we are likely to get different scoring. Please review and advise. Please could you show the additional aspect of ‘whenever’ in the FT rev1, plus the BT of rev1? could you show the aspect of ‘whenever’ or ‘as I wish’ in the FT rev1, plus the BT rev1?  AM-13-Sept-2011. Whenever was added  RHUL (AW+RP) 6-Oct-11: Thank you🗸 |
| **FT rev1** | آزاد بودن در نوشیدن هر چیز و هر زمانی که می خواهم، برای من ............. |
| **BT rev1** | For me, freedom to drink whatever and whenever I want…………. . |
| **Comments** |  |

| Item | (19b) responses |
| --- | --- |
| **OE** | very important – important – somewhat important – not at all important |
| **FT-rec** | خیلی مهم است، مهم است، تا حدی مهم است، اصلا مهم نیست |
| **BT1** | Are very important, are important, are almost important, are not important at all.  Are very important, are important, are almost important, are unimportant. |
| **BT2** | Very important, Important, Somewhat important, Not important at all |
| **Comments** | AM-20-Oct-2010. BT2 is wrong. In BT1 **‘are’** should be **‘is’**. However, FT-rec is acceptable.  RHUL (AW+RP) 13-Dec-10: Please see our comment for item 1b responses concerning *almost*.  AM-13-Sept-2011. Ft-rec satisfies your concern.  RHUL (AW+RP) 6-Oct-11:🗸 |
| **FT rev1** |  |
| **BT rev1** |  |
| **Comments** |  |

| Item | Closing instruction |
| --- | --- |
| **OE** | If there are any other ways in which diabetes, its management and any complications affect your quality of life, please say what they are below: |
| **FT-rec** | اگر در خصوص دیابت، درمان و عوارض آن موضوعات دیگری وجود دارد که بر کیفیت زندگی شما اثر می گذارد، لطفا آنها را در قسمت زیر بنویسید. |
| **BT1** | If there are other matters about diabetes, its therapy and complications which influence your quality of life, please write them in the following section (or part) |
| **BT2** | If there are other issues relating to diabetes, its treatment and symptoms that affect your quality of life, please write them in the space below. |
| **Comments** | AM-20-Oct-2010. O.K.  RHUL (AW+RP) 13-Dec-10: Does the FT-rec show ‘complications’? BT2 has ‘symptoms’ and this is not quite correct. Please see our comments for the similar wording on p.1.  AM-13-Sept-2011.The word ‘عوارض’ is translation of ‘complications’ and this exists in FT-rec and BT1. I think FT-rec is O.K.  RHUL (AW+RP) 6-Oct-11: Thank you. 🗸 |
| **FT rev1** |  |
| **BT rev1** |  |
| **Comments** |  |

| Item | Final “thank-you” |
| --- | --- |
| **OE** | Thank you for completing this questionnaire |
| **FT-rec** | از همکاری شما در تکمیل این پرسشنامه متشکریم |
| **BT1** | Thanks for your cooperation in completing the questionnaire. |
| **BT2** | Thank you for your cooperation in completing this questionnaire |
| **Comments** | AM-20-Oct-2010. O.K.  RHUL (AW+RP) 13-Dec-10:🗸 |
| **FT rev1** |  |
| **BT rev1** |  |
| **Comments** |  |

| Item | Page numbering |
| --- | --- |
| **OE** | Page x of y |
| **FT-rec** | صفحه x از y |
| **BT1** | Page x of y |
| **BT2** | Page X of Y |
| **Comments** | AM-20-Oct-2010. O.K.  RHUL (AW+RP) 13-Dec-10:🗸 |
| **FT rev1** |  |
| **BT rev1** |  |
| **Comments** |  |
